# Tailor‐Made Vessels for Enhancing the Mechanochemical Energy Transfer and Milling Efficiency

**DOI:** 10.1002/cssc.202502452

**Published:** 2025-12-02

**Authors:** Tim Jansen, Lisa Cassandra Thomas, Carsten Bolm

**Affiliations:** ^1^ Institute of Organic Chemistry RWTH Aachen University Aachen Germany

**Keywords:** 3D‐printing, ball mill, jar geometry, mechanochemistry, milling vessel

## Abstract

Mechanochemistry has gained momentum in organic synthesis. Often, shaker ball mills are used. The geometry of their vessels leads to different action modes, some with high and some with low energy input. These motions interchange chaotically, and new vessels with alternative geometries revealing defined action modes shall lead to a deeper understanding of mechanochemical systems and allow improvements. We designed twenty new jar geometries and 3D printed such vessels using an UV‐resin‐based printer and a translucent polymer. For analyzing the energy‐rich impacts in these new geometries, a setup with triboluminescence in connection with high‐speed footage was introduced. In this manner, the number of hits with enough energy to emit a light reflex was determined. To further investigate the efficiency of the new geometries, time measurements of light decays were conducted. Several vessels showed great improvements in hit counts as well as in milling time reduction until total light decay, confirming the importance of newly designed mechanochemical vessels with tailor‐made geometries enhancing the milling efficiency of mechanochemical systems.

## Graphical abstract

In ball mills, the vessel geometry has a major impact on the milling efficiency. By 3D‐printing, 18 new vessels were introduced, and their ability to direct the milling balls for high‐level energy transfers was investigated by a novel experimental set‐up following the triboluminescent behavior of a copper complex by high‐speed photography.

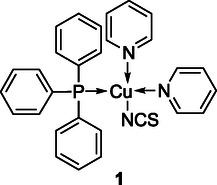

## 1 Introduction

Mechanochemistry has become a widely used synthetic technology with many researchers around the world benefiting from performing highly energy effective reactions under solvent‐free conditions. Most processes utilize mechanical energy generated by machines like ball mills and extruders [1, 2, 3, 4, 5, 6, 7, 8, 9, 10, 11, 12, 13, 14, 15, 16, 17, 18, 19, 20, 21, 22, 23, 24, 25, 26, 27, 28, 29, 30, 31, 32, 33]. The mechanical forces depend on the individual system and mainly arise from impacts, pressure, shear, and friction of two or more objects. Because the reactions are not mediated by solvents, blending and mixing are particularly important. If that is done well, mechanochemistry can enable reactions that are uncommon or even unprecedented in classical solvent‐based chemistry. Overall, mechanochemistry is recognized as an opportunity to perform advanced synthesis in a greener and more benign manner [34, 35, 36, 37, 38].

With new reactions and synthetic protocols discovered daily, mechanochemistry has become a highly dynamic field. The equipment, however, appears rather static. Mostly, a small set of planetary or mixer mills from a limited number of suppliers is employed. For reactions performed in continuous mode, standard commercially available twin‐screw extruders or resonant acoustic mixers are applied [39, 40]. Commonly, the milling jars have very similar shapes, and the balls only vary in size, mass, and material [41]. That is surprising because both jars and milling bodies have a tremendous effect on the milling. Their shapes determine the dynamics and the motion pattern in the machine with consequences for impact, pressure, shear, friction, etc. [42, 43, 44]. Studying such movements inside the milling jar is challenging. Budroni and coworkers, for example, investigated the dynamics of the milling bodies inside milling jars with either a flat or a curved base [45]. While in the former, impacts occurred at a few points of the reactor wall, the latter favored a sliding along the reactor borders. Practically, this meant that in the curved‐base reactor, wider regions of chaotic dynamics and more collisions resulted, which translated to a more efficient mixing with a higher probability of inducing a transformation compared to the flat reactor. Cao and coworkers simulated the spheres motion in a mixer mill using a 3D dynamic model [46]. They found a disordered behavior for the trajectories of the spheres and observed that most impacts occurred on the cylindrical surface of the vial along the tangential direction [47, 48]. The movement of the milling bodies in the jar will also affect the impact forces [49, 50] and the kinetics of a mechanochemical reaction [51, 52].

Imaging technologies proved very instructive in analyzing the grinding ball motion pattern [47, 53] and following the reaction progress [54]. In this context, Borchardt and coworkers just reported a tracking of the ball movement in an MM500 mixer mill by high‐speed video recording [53]. Their study also included two variations of the milling bodies and the vessel, and the authors concluded that “even seemingly minor geometric changes can impact the movement behavior and, consequently, reaction efficiency”. Our investigation goes beyond that. We 3D‐printed a series of (18) vessels with very different geometries for a mixer mill and analyzed the ball motion in those vessels by detecting the triboluminescence of a copper complex upon milling by high‐speed photography. The results and observations are summarized here.

## 2 Results and Discussion

### 2.1 Experimental Design and Set‐up

The use of transparent vessels is well established in mechano‐chemistry [47, 53, 54, 55], and they have found numerous applications in in situ monitoring [56, 57] and solid‐state photocatalysis [58, 59, 60, 61, 62]. In our study, we used polymer vessels made out of a translucent UV resin, which were applied in an Insolido IST 636 mixer mill. Tracking of a single milling ball in a transparent milling jar by high‐speed footage showed that there were two main modes of action: First, a “hitting mode”, where the ball oscillates between the two ends of the jar, and, second, a “rolling motion”, where the milling ball moved in the middle of the cylinder and rolled alongside the circumference of it. These two modes are frequently interchanged with each other [63].

**FIGURE 1:**
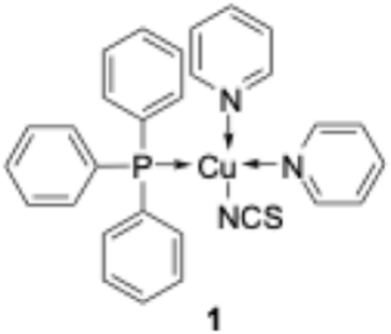
Copper(I) complex **1** is used as a triboluminescent indicator.

While the path of the ball could be followed by a single frame analysis of the high‐speed video, the method did not allow an estimate of the kinetic energy associated with that move. To assess if the ball had enough power to potentially induce a chemical transformation, we envisaged the use of triboluminescent crystals as indicators. Among several options, copper(I) complex **1** (Figure 1), which is easily accessible in large quantities [64, 65, 66, 67], appeared most suitable. When broken, crystals of **1** emit ice blue light with a wavelength of about 426 nm.

**FIGURE 2:**
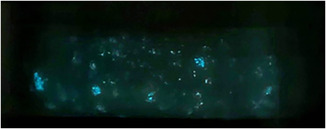
Long exposure photograph of the triboluminescence within a mixer mill environment. The picture was taken with ISO50 and 2 sec exposure. The contrast and brightness were altered posterior. The mill had a frequency of 25 Hz and was loaded with 100 mg of triboluminescent crystals **1**.

We hypothesized that every hit by a ball with enough energy to result in a crystal breakage should be trackable by the triboluminescence. Determining the position of the induced light would then allow a mapping of the energy‐sufficient hits. Because the triboluminescence intensity depends on the crystal size, the method could also be used for endpoint determinations, as indicated by a light decay and fading. The first photograph of such an experiment is shown in Figure 2. It is a long exposure image, which reveals areas with denser light reflexes. However, since the mill was moving while the picture was taken, these light reflexes did not accurately match their actual position in the ball mill vessel.

**FIGURE 3:**
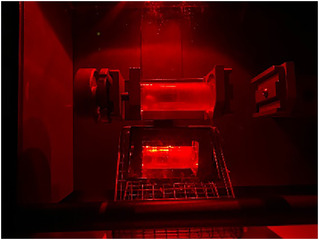
Experimental setup to record high‐speed footage of the breakage of triboluminescent crystals of **1** in an Insolido IST 636 mixer mill. A mirror was placed underneath the milling jar and a flashlight was placed on top of the lid of the mill.

To improve the approach of using the light flashes as an indicator for energy‐sufficient hits, the milling of the triboluminescent crystals was followed by using a high‐speed camera. To do so, a flashlight was installed, allowing the camera to focus on the jar and to accentuate all moving parts during the milling. Red light was chosen to avoid an overglow of the blue flashes of the triboluminescent crystals. As a result, individual light flashes could be localized, leading to a significantly higher resolution over time. Furthermore, the light reflexes were mapped in conjunction with the milling ball movement. Placing a mirror underneath the milling jar gave a second perspective of the motion. The experimental setup is shown in Figure 3.

With this setup, it was possible to quantify the number of hits by analyzing the high‐speed footage and counting the light reflexes. The method visualized those contacts of the milling ball and the wall that possessed enough force to break the crystals. By the absence of flashes, the videos revealed that the rolling mode did not lead to crystal breakings, whereas the hitting mode provided many wall contacts with great enough force to break crystals and emit light. For each measurement, 100 mg of the triboluminescent copper complex crystals were milled for 3 sec, and six high‐speed videos with half a second timeframe and 960 fps each were taken. The triboluminescent crystals were renewed after every second video. In this manner, we tried to minimize the effect of particle size reduction, resulting in less light reflexes [68].

**FIGURE 4:**
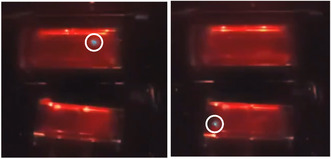
Exemplary pictures of hits with light reflexes (marked by white circles). The pictures were obtained by a single frame of a high‐speed video. The left picture shows a hit from the front view of the upper half. The right picture shows a hit from the bottom view of the lower half.

Exemplary pictures of hits with light reflexes in front and bottom views are shown in Figure 4.

**FIGURE 5:**
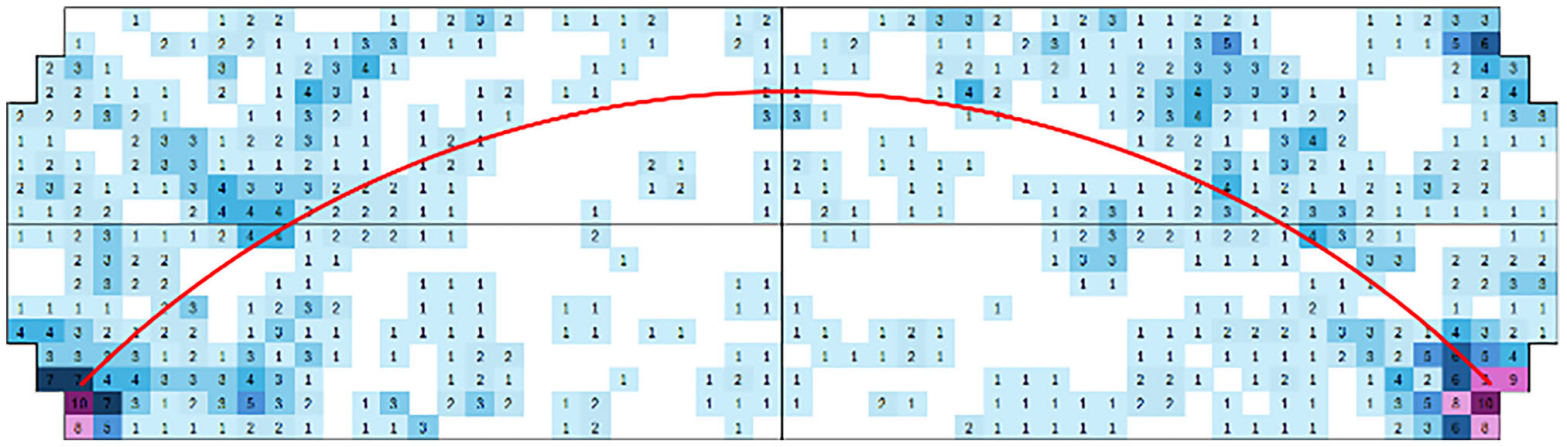
Hit map of the front view of a cylindrical milling vessel, at 25 Hz (as *reference*). The numbers represent the number of hits occurring in a time span of 3 sec. Each number of hits was assigned a color, the red line shows the preferred path of the milling ball within the system.

By quantifying these modes of action, it became apparent that the milling ball followed preferred pathways in the hitting mode. These pathways are visually compared to standing waves, which were also found in the simulations by Budroni and coworkers [45]. Figure 5 shows a “hit map” for the movement of a single milling ball in a vessel with cylindrical shape (further described as *reference*). It was prepared by counting all (1′378) individual hits and mapping them in a schematic drawing of the milling vessel. Each color corresponds to a defined number of hits listed in the field at the exact spot. A red arch was drawn, highlighting the preferred path of the ball, indicating the hit density at these points.

Although the milling ball pathway could clearly be assigned, the individual hit numbers varied from run to run due to the overall chaotic nature of the system. Thus, every hit and contact between the ball and the wall drastically influences the motion. In addition, the motion of the jar itself played a role by not being translational, but radial on a circle defined by the length of the milling arm. Lastly, gravity was important due to the downward component of every movement of the ball. Overall, the movement of the ball inside the milling jar was too chaotic to allow predictions. In order to obtain statistically relevant data, repetitions of video recordings were crucial. Adding all hit numbers stemming from hundreds of individual wall contacts of the ball collected in six videos and treating them as one big data point finally provided statistically relevant pathways of the milling ball.

### 2.2 Variations of the Vessel Geometries

Realizing the value of the hit maps in indicating the motion and power of the milling balls, we wondered about the effects induced by vessels with other geometries. The *reference* system could serve for comparison.

**FIGURE 6:**
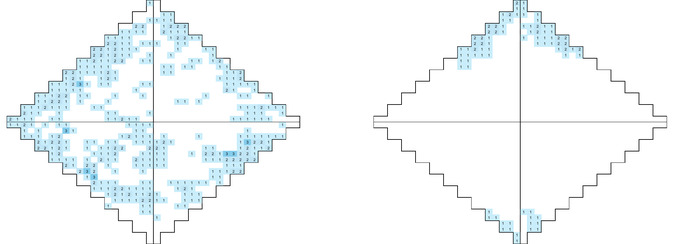
Hit maps of the *hollow sphere vessel* at 25 Hz. Left: Front view; right: Bottom view.

As mentioned before, the previous observations suggested that most of the crystal breakings occurred in the hitting mode. Thus, promoting a rolling of the ball in a vessel with altered geometry was expected to reduce the system's efficiency as indicated by a lower number of light flashes of the triboluminescent crystals during the milling. This hypothesis was confirmed by applying a *hollow sphere vessel* lacking flat areas and obstacles, therefore allowing the milling ball to show a free rolling motion without hitting the vessel walls. The respective hit map is shown in Figure 6.

The hit map of the experiment with the *hollow sphere vessel* (Figure 6) was characterized by 494 flashes from the side and 100 from the bottom. Compared to the 1′378 hits in the run with the *reference* system, the hit amount was less than half. Thus, the assumption that the here promoted rolling mode led to a lower energy transfer as compared to the hitting mode was supported.

**FIGURE 7:**
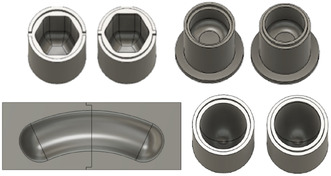
Vessels with newly designed geometries. From top left: *Hexagon* with rounded edges; top right: *Short and thick* vessel; bottom left: *Banana* vessel; bottom right: *Ellipsoid* vessel. These pictures were obtained by the 3D‐Modeling Software Fusion 360.

By the next set of experiments, we aimed at minimizing the rolling and promoting the hitting mode. As shown in Figure 7, several vessels with altered geometries were designed and 3D‐printed.

The first designed vessel was a rounded *hexagon*, prohibiting the rolling motion by edges and flat surfaces, which suppressed an undisturbed circular motion within this hexagonal shape. In the second one, called *short & thick*, the length of the vessel was changed so that the amplitude of the ball mill was greater than the inner length of the jar. Thus, if the ball entered the rolling mode, it collided at the end of the jar and was pushed back into the hitting mode. With the idea of more closely relating to the angular motion of the milling arms than to the amplitude of the machine, a *banana* (rounded cylinder with curvature) was designed as the third vessel. This shape prohibited the circular motion in two ways: First, the curvature disturbed the path of the milling ball due to different way lengths within the circular motions’ radius, and second, the curvature, which resembled the radial motion of the milling arm, transferred the force of the machine differently to the milling ball. Since this shape was not symmetrical it could be fixed in the machine in various directions. Here, two alignments with respect to the convex site were selected which either faced the camera (*banana* orientation 1) or, alternatively, the machine (*banana* orientation 2). Because only the first one was aligned with the angular movement of the machine, the results of the *banana* orientation 2 will be discussed later. Vessel number 4 had an *ellipsoid* shape where the hollow parts rejuvenated toward the end. As such, it was a round body without edges, and in principle, the ball could freely reach each spot. The rejuvenations, however, hampered a steady rolling motion of the ball because it became faster in these areas. To sustain this increased rolling motion, more energy was required than for moving in a wider cylinder, leading to an overall impediment of this motion mode. The results of the hit counts for these four new vessels, also in relation to the *reference* system, are shown in Table 1.

**TABLE 1:** Comparison of the hit counts in vessels with different geometries.

	Number of hits		
	Front view	Bottom view	Overall
*Ellipsoid*	1′646	1′449	3′095
*Banana* orientation 1	1′354	737	2′091
*Reference*	867	511	1′378
*Hexagon*	667	323	990
*Short & thick*	450	167	617

Analyzing the hit maps of all new vessels showed that in none of the geometries did a rolling motion occurr. To our surprise, however, the number of hits varied significantly. The top with 3095 overall hits was observed for the *ellipsoid*‐shaped vessel, followed by the *banana* orientation 1 with 2′091 overall hits. Both vessels were remarkably better than the *reference* system with 1′378 overall hits. This sequence was in line with the numbers of the front and bottom views. In contrast, the *hexagon* and the *short & thick* vessel performed differently, and all hit count numbers (front and bottom views as well as overall) were lower. Probably, the additional edges that could not be hit as frequently reduced the number for the *hexagon*. In addition, there were more hits in the middle of the flat surfaces than on the edges, which led to less prominent pathways as compared to the *reference* jar. The moderate number for the *short & thick* vessel could be due to an inherent lower energy of the ball resulting from the shorter moving path with a reduced velocity. In addition, for this vessel type, the measuring itself was challenging. Because the hit observation was only possible in the vessel parts framed by the camera, the difference in surface area of the walls and the ends, as well as the larger diameter of the termini, became relevant, and both effects might have led to an underestimation of the hit amount.

**FIGURE 8:**
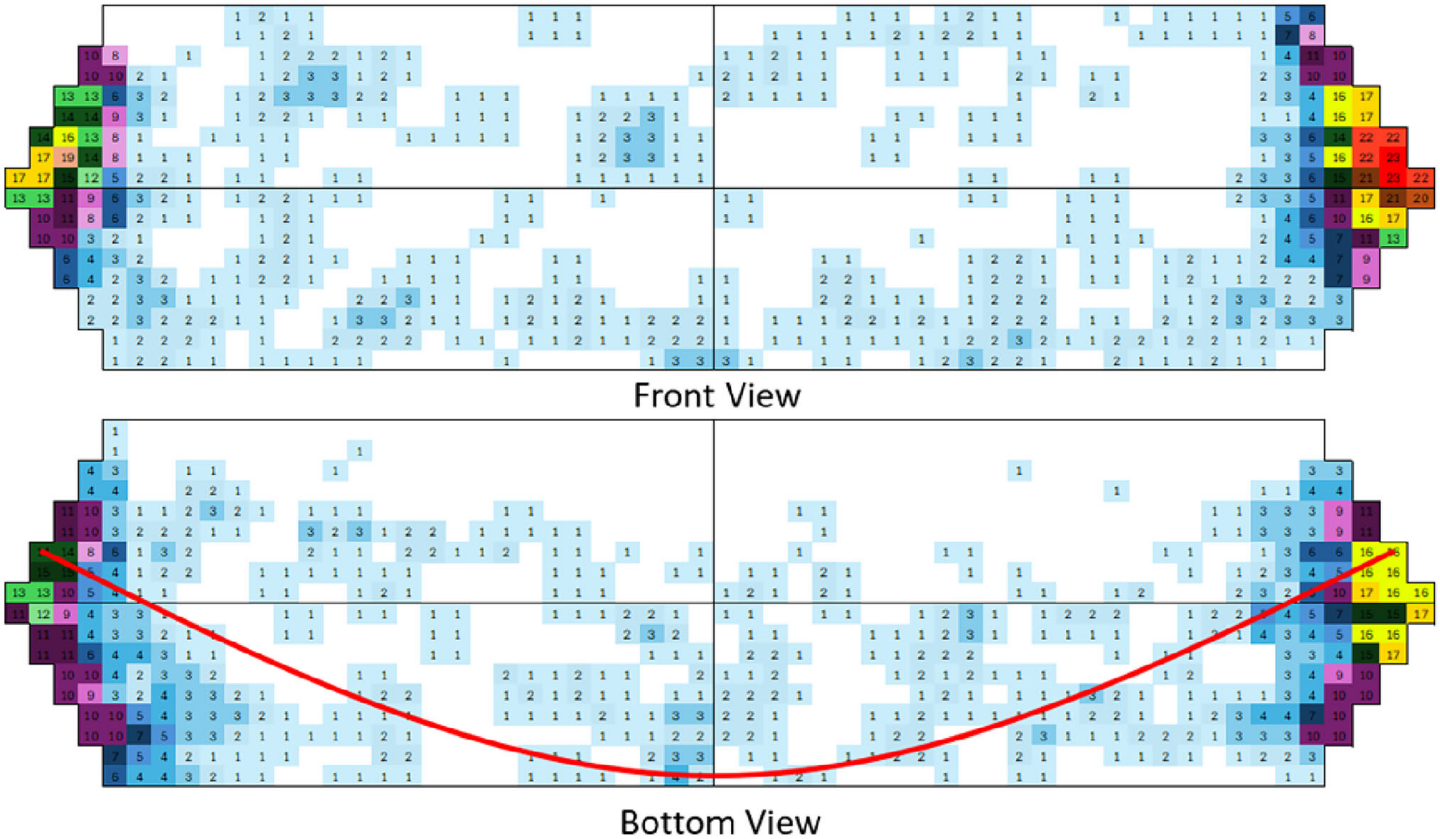
Hit maps of the *ellipsoid* milling vessel at 25 Hz. Top: Front view; Bottom: Bottom view. The numbers shown are the hit count within 3 sec of milling and footage time. Each number is equally represented by its own color. In the bottom view, the red line shows the preferred path of the milling ball.

The remarkable result for the *ellipsoid*‐shaped vessel with the large number of hits could be used for visualizing the major pathway of the ball inside the vessel. The related hit map is shown in Figure 8.

In the *ellipsoid*‐shaped vessel, the pathway of the milling ball is defined by the centrifugal forces induced by the radial motion of the milling arms. Due to this force, the hits concentrate on the front half of the milling jar. In contrast to the path seen at the bottom, the front view does not show a defined ball motion. In addition, the number of hits at the ends of the vessel was significantly higher than those in the middle of the jar. Because in this manner, the ball can build up more momentum on its way, a higher energy transfer can be achieved. Noteworthy is also the observation that within the analyzed timespan, the ball did not change the mode of action once it started to hit the ends of the vessel.

**FIGURE 9:**
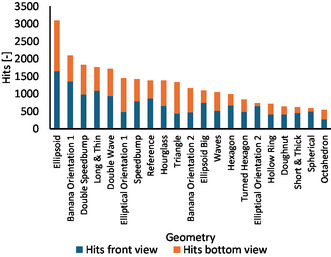
Hit numbers of various vessel geometries. (Details of the shapes can be found in the Supporting Information). The overall hit number is split into the one that occurred in the front view (blue) and in the bottom view (orange). The geometries were sorted with decreasing hit count.

With the intention to gain more insight into the milling behavior of vessels with varying geometries, the investigation was extended, and more jars were designed and 3D printed. These geometries were selected after looking at the modes of action and the preferred pathways in the *reference* geometry. Some were designed by using obstacles like speed bumps, others had curved wave patterns, which shaped new ways for the milling balls, and some had flat surfaces to change the pattern of the deflected hits of the milling balls. (A full list with geometries and assigned names can be found in the Supporting Information). Figure 9 summarizes and compares the number of counted hits for each tested geometry.

The results shown in Figure 9 support the initial assumption that geometries, which promote hits over rolling motion, increase the number of visible crystal breakages. In addition, the larger the visible surface area, the more identifiable the hits. Even with the uncertainty that not all hits could be detected due to geometric constraints of the experimental setup, the trends were clear. These issues shall be addressed in future work by redesigning the method of filming and by adding mirrors for additional observing angles. Another interesting point was that the angular motion of the ball mill arm had a great impact on the system. Considering this aspect was relevant, as confirmed by the geometry with the second most hits, the *banana* with a rounded‐off cylinder extruded over a curved path.

### 2.3 Variations of the Milling Frequency

**FIGURE 10:**
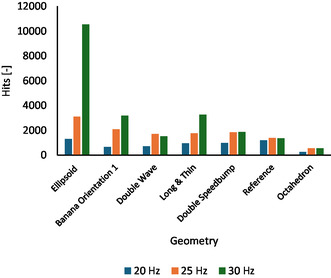
Overall hit numbers of selected geometries for milling frequencies of 20, 25, and 30 Hz. The geometries were sorted by the number of hits at 25 Hz.

In the next step, the influence of the milling frequency on the hit number was investigated. The study involved the vessels with the five most efficient geometries, the *reference* system, and the worst‐performing geometry (as deduced from Figure 9). The measurements were conducted at 20 Hz, 25 Hz, and 30 Hz. The results are shown in Figure 10.

With the exception of the *reference* jar, which showed almost identical hit numbers over the entire frequency range, all other vessels were strongly affected by the milling frequency, generally showing an increasing number of hits from the low to the higher frequency values. The effects were most significant for the *ellipsoid*, the *banana* orientation 1, and the *long & thin* geometries. For the *ellipsoid* vessel, the hit count even reached the remarkable value of 10′529 when milled at a frequency of 30 Hz. Otherwise, the closest numbers to this maximum were around 3′000 hits. Also at 20 Hz, the *ellipsoid* vessel was best, with 1′301 hits being the highest hit count of all investigated jars at that frequency. Apparently, each vessel had “its own character”, which was determined by its geometry and implied level of symmetry. The jars, which guided the ball from one side to the other, increased the number of hits when changing the frequency from low to high. The *double speedbump* and the *double wave* being characterized by internal obstacles for the ball, increased the hits when going from 20 to 25 Hz. At 30 Hz, however, the motion became unpredictable and chaotic, which might be beneficial for mixing, but here, led to a decrease of the finally detected force‐impact.

### 2.4 Time of the Triboluminescence Decay

**FIGURE 11:**
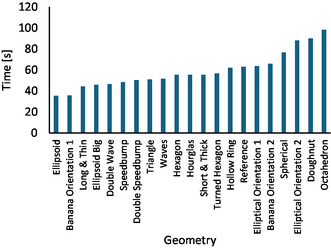
Milling time until the decay of the triboluminescence. The milling vessel was fixed in an MM400 mixer mill and charged with 50 mg of [Cu(NCS)(py)_2_(PPh_3_)] and a 10 mm PTFE ball. At 25 Hz, the milling was continued until the light emission of the triboluminescent copper complex ended.

As mentioned above, the triboluminescence of the copper crystals is size‐dependent [64, 65, 66, 67]. Grinding them to a fine powder decreases the light intensity until it disappears. Thus, measuring the time span until the lighting stopped offered an additional tool for analyzing the milling force in each vessel. For this measurement, the room was fully darkened, and an MM400 mixer mill (RETSCH) was used. Each vessel was fixed in the apparatus, and under standard milling conditions, the time until the flashing of the triboluminescence ended was measured. The results of these experiments are shown in Figure 11.

The time measurements confirmed the overall picture provided by the data of the hit counts. The *ellipsoid* was the most efficient vessel shape, followed by the *banana* orientation 1, which led to a light fading after 35 and 36 s, respectively. At the other end was the *octahedron* again, where the light emission stopped after 98 s of milling. Between these two extremes, all other vessel geometries could be found with the *reference* jar being in the lower third of the field, with a milling time of 63 s until complete light extinguishing. Compared to the *reference* jar and considering the earlier data (Figure 9), the result for the *short and thick* vessel with 55 s was surprising. Either the crushing of the big crystals was really more effective (although the hit number was lower, see Figure 9) or, for this vessel type, the light detection was error‐prone due to an observation problem in the middle of the flat surfaces at the vessel ends. Unfortunately, neither of the two options can unequivocally be excluded at this stage.

## 3 Conclusion

This study focused on two aspects: First, on analyzing the movements and impact forces of the milling balls by introducing an experimental set‐up utilizing a triboluminescent copper complex, and second, on enhancing the force transmission in mechanochemical systems by vessel design and geometry optimization.

Well‐investigated [Cu(NCS)(py)_2_(PPh_3_)] was applied for visualizing ball impacts with enough force to break the crystals, resulting in light emission. With high‐speed imaging, the flashes were mapped, and the ball pathways were identified. In the vessels of the mixer mill, two major action modes of the balls were relevant. A rolling mode, which did not significantly contribute to the internal force transfer, and a hitting mode leading to a high energy transmission. By 3D printing, 18 new vessels with altered geometries were introduced. Testing, comparing, and categorizing them in 20 orientations allowed for to disfavoring of the rolling mode, resulting in a drastic increase in the number of light flashes, indicating more powerful hits, leading to crystal breakage. An *ellipsoid*‐formed vessel outperformed the *reference* cylindrical vessel by orders of magnitude. For most vessel types, the milling frequency had a major impact. Measuring the time until the total decay of the light reflexes, which correlates with the crystal size, supported the first results. Again, the *ellipsoid* performed best as indicated by the short time span until triboluminescence termination.

Overall, our data show that the vessel geometry drastically impacts the milling effectiveness. Adjusting it allows for fine‐tuning the ball movement, and, if desired, to increase the number of energy‐sufficient hits. 3D‐printing opens the field for tailor‐made jar geometries, providing multiple opportunities for less energy‐consuming, more effective, and greener approaches in academia and the chemical industry.

## Supporting Information

Additional supporting information can be found online in the Supporting Information section. **Supporting Fig. S1:** Hit maps of the Geometry *Banana* Orientation 1 at 25 Hz. Top: Front view; Bottom: Bottom view. The numbers shown are the hit counts within 3 s of milling and footage time. Each number is equally represented by its own color. **Supporting Fig. S2:** Hit maps of the geometry *Banana* Orientation 2 at 25 Hz. Top: Front view; Bottom: Bottom view. The numbers shown are the hit counts within 3 s of milling and footage time. Each number is equally represented by its own color. **Supporting Fig. S3:** Hit maps of the geometry *Double Speedbump* at 25 Hz. Top: Front view; Bottom: Bottom view. The numbers shown are the hit counts within 3 s of milling and footage time. Each number is equally represented by its own color. **Supporting Fig. S4:** Hit maps of the geometry *Double Wave* at 25 Hz. Top: Front view; Bottom: Bottom view. The numbers shown are the hit counts within 3 s of milling and footage time. Each number is equally represented by its own color. **Supporting Fig. S5:** Hit maps of the geometry *Donut* at 25 Hz. Top: Front view; Bottom: Bottom view. The numbers shown are the hit counts within 3 s of milling and footage time. Each number is equally represented by its own color. **Supporting Fig. S6:** Hit maps of the geometry *Ellipsoid* at 25 Hz. Top: Front view; Bottom: Bottom view. The numbers shown are the hit counts within 3 s of milling and footage time. Each number is equally represented by its own color. **Supporting Fig. S7:** Hit maps of the geometry *Ellipsoid* big at 25 Hz. Top: Front view; Bottom: Bottom view. The numbers shown are the hit counts within 3 s of milling and footage time. Each number is equally represented by its own color. **Supporting Fig. S8:** Hit maps of the geometry *Elliptical* Orientation 1 at 25 Hz. Top: Front view; Bottom: Bottom view. The numbers shown are the hit counts within 3 s of milling and footage time. Each number is equally represented by its own color. **Supporting Fig. S9:** Hit maps of the geometry *Elliptical* Orientation 2 at 25 Hz. Top: Front view; Bottom: Bottom view. The numbers shown are the hit counts within 3 s of milling and footage time. Each number is equally represented by its own color. **Supporting Fig. S10:** Hit maps of the geometry *Hexagon* at 25 Hz. Top: Front view; Bottom: Bottom view. The numbers shown are the hit counts within 3 s of milling and footage time. Each number is equally represented by its own color. **Supporting Fig. S11:** Hit maps of the geometry *Hollow Ring* at 25 Hz. Top: Front view; Bottom: Bottom view. The numbers shown are the hit counts within 3 s of milling and footage time. Each number is equally represented by its own color. **Supporting Fig. S12:** Hit maps of the geometry *Hourglass* at 25 Hz. Top: Front view; Bottom: Bottom view. The numbers shown are the hit counts within 3 s of milling and footage time. Each number is equally represented by its own color. **Supporting Fig. S13:** Hit maps of the geometry *Long & Thin* at 25 Hz. Top: Front view; Bottom: Bottom view. The numbers shown are the hit counts within 3 s of milling and footage time. Each number is equally represented by its own color. **Supporting Fig. S14:** Hit maps of the geometry *Octahedron* at 25 Hz. Top: Front view; Bottom: Bottom view. The numbers shown are the hit counts within 3 s of milling and footage time. Each number is equally represented by its own color. **Supporting Fig. S15:** Hit maps of the geometry *Reference* at 25 Hz. Top: Front view; Bottom: Bottom view. The numbers shown are the hit counts within 3 s of milling and footage time. Each number is equally represented by its own color. **Supporting Fig. S16:** Hit maps of the geometry *Short & Thick* at 25 Hz. Top: Front view; Bottom: Bottom view. The numbers shown are the hit counts within 3 s of milling and footage time. Each number is equally represented by its own color. **Supporting Fig. S17:** Hit maps of the geometry *Speedbump* at 25 Hz. Top: Front view; Bottom: Bottom view. The numbers shown are the hit counts within 3 s of milling and footage time. Each number is equally represented by its own color. **Supporting Fig. S18:** Hit maps of the geometry *Spherical* at 25 Hz. Top: Front view; Bottom: Bottom view. The numbers shown are the hit counts within 3 s of milling and footage time. Each number is equally represented by its own color. **Supporting Fig. S19:** Hit maps of the geometry *Triangle* at 25 Hz. Top: Front view; Bottom: Bottom view. The numbers shown are the hit counts within 3 s of milling and footage time. Each number is equally represented by its own color. **Supporting Fig. S20:** Hit maps of the geometry *Turned Hexagon* at 25 Hz. Top: Front view; Bottom: Bottom view. The numbers shown are the hit counts within 3 s of milling and footage time. Each number is equally represented by its own color. **Supporting Fig. S21:** Hit maps of the geometry *Waves* at 25 Hz. Top: Front view; Bottom: Bottom view. The numbers shown are the hit counts within 3 s of milling and footage time. Each number is equally represented by its own color. **Supporting Fig. S22:** Hit maps of the geometry *Banana* Orientation 1 at 20 Hz. Top: Front view; Bottom: Bottom view. The numbers shown are the hit counts within 3 s of milling and footage time. Each number is equally represented by its own color. **Supporting Fig. S23:** Hit maps of the geometry *Banana* Orientation 1 at 30 Hz. Top: Front view; Bottom: Bottom view. The numbers shown are the hit counts within 3 s of milling and footage time. Each number is equally represented by its own color. **Supporting Fig. S24:** Hit maps of the geometry *Double Speedbump* at 20 Hz. Top: Front view; Bottom: Bottom view. The numbers shown are the hit counts within 3 s of milling and footage time. Each number is equally represented by its own color. **Supporting Fig. S25:** Hit maps of the geometry *Double Speedbump* at 30 Hz. Top: Front view; Bottom: Bottom view. The numbers shown are the hit counts within 3 s of milling and footage time. Each number is equally represented by its own color. **Supporting Fig. S26:** Hit maps of the geometry *Double Wave* at 20 Hz. Top: Front view; Bottom: Bottom view. The numbers shown are the hit counts within 3 s of milling and footage time. Each number is equally represented by its own color. **Supporting Fig. S27:** Hit maps of the geometry *Double Wave* at 30 Hz. Top: Front view; Bottom: Bottom view. The numbers shown are the hit counts within 3 s of milling and footage time. Each number is equally represented by its own color. **Supporting Fig. S28:** Hit maps of the geometry *Ellipsoid* at 20 Hz. Top: Front view; Bottom: Bottom view. The numbers shown are the hit counts within 3 s of milling and footage time. Each number is equally represented by its own color. **Supporting Fig. S29:** Hit maps of the geometry *Ellipsoid* at 30 Hz. Top: Front view; Bottom: Bottom view. The numbers shown are the hit counts within 3 s of milling and footage time. Each number is equally represented by its own color. **Supporting Fig. S30:** Hit maps of the geometry *Long and Thin* at 20 Hz. Top: Front view; Bottom: Bottom view. The numbers shown are the hit counts within 3 s of milling and footage time. Each number is equally represented by its own color. **Supporting Fig. S31:** Hit maps of the geometry *Long and Thin* at 30 Hz. Top: Front view; Bottom: Bottom view. The numbers shown are the hit counts within 3 s of milling and footage time. Each number is equally represented by its own color. **Supporting Fig. S32:** Hit maps of the geometry *Octahedron* at 20 Hz. Top: Front view; Bottom: Bottom view. The numbers shown are the hit counts within 3 s of milling and footage time. Each number is equally represented by its own color. **Supporting Fig. S33:** Hit maps of the geometry *Octahedron* at 30 Hz. Top: Front view; Bottom: Bottom view. The numbers shown are the hit counts within 3 s of milling and footage time. Each number is equally represented by its own color. **Supporting Fig. S34:** Hit maps of the geometry *Reference* at 20 Hz. Top: Front view; Bottom: Bottom view. The numbers shown are the hit counts within 3 s of milling and footage time. Each number is equally represented by its own color. **Supporting Fig. S35:** Hit maps of the geometry *Reference* at 30 Hz. Top: Front view; Bottom: Bottom view. The numbers shown are the hit counts within 3 s of milling and footage time. Each number is equally represented by its own color. **Supporting Table S1:** Used printing settings for the High Clear Resin as used in Chitubox Basic software. **Supporting Table S2:** Hit counts of the geometries at 25 Hz ordered alphabetically. The *reference* geometry is marked in light gray. **Supporting Table S3:** Hit counts of the geometries at frequencies of 20 and 30 Hz. The *reference* geometry is marked in gray. **Supporting Table S4:** Time measurements until complete light decay of 50 mg of triboluminescent crystals.

## Funding

This work was supported by Deutsche Forschungsgemeinschaft (390919832).

## Conflicts of Interest

The authors declare no conflicts of interest.

## Supporting information

Supplementary Material

## Data Availability

The data that support the findings of this study are available from the corresponding author upon reasonable request.
