# Supplementary material for: Tailor‐Made Vessels for Enhancing the Mechanochemical Energy Transfer and Milling Efficiency
Source: ChemSusChem. 2025 Dec 2;19(1):e202502452. doi: 10.1002/cssc.202502452 (PMC12767565; doi:10.1002/cssc.202502452)
Supplement: Supplementary file 1 — Supplementary Material [file CSSC-19-e202502452-s001.pdf]

Supporting Information
©Wiley-VCH 2021
69451 Weinheim, Germany

Table of Contents

1.	Technical drawings of the different geometries	S3
2.	3D printing of the geometries	S22
3.	Synthesis of copper(I) thiocyanate bipyridine triphenylphosphine	S23
4.	Experiments to obtain the hit maps	S24
5.	Hit maps and hit counts of every geometry	S24
6.	Frequency variation and effect on hit maps and hit counts	S40
7.	Time measurements of the light decay	S50
8.	References	S51

1. Technical drawings of the different geometries

In the following, the technical drawings of the different geometries are shown. They were done using the Fusion 360 software. All technical drawings are scaled 1:1 when the whole drawing is printed on an A3 sheet of paper. Due to technical reasons, the following drawings are scaled 50% to an A4 format. In this format, the scale is 1:2. For further information regarding the PDF format of the technical drawing and 3D files, contact the authors.

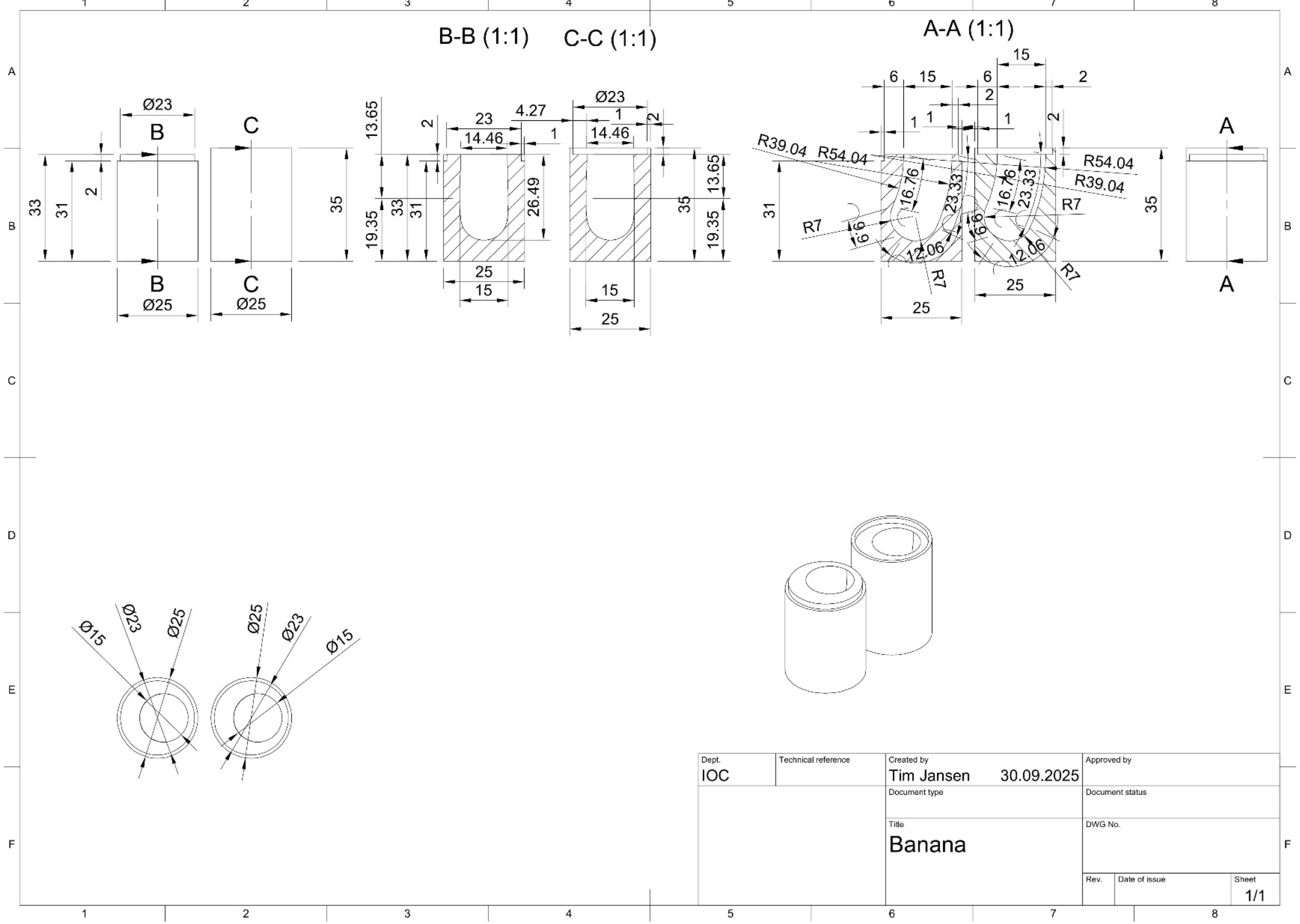

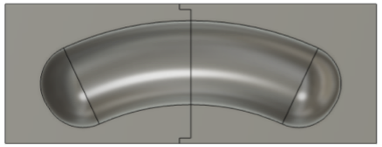

The 3D-model was prepared by Lisa Thomas.

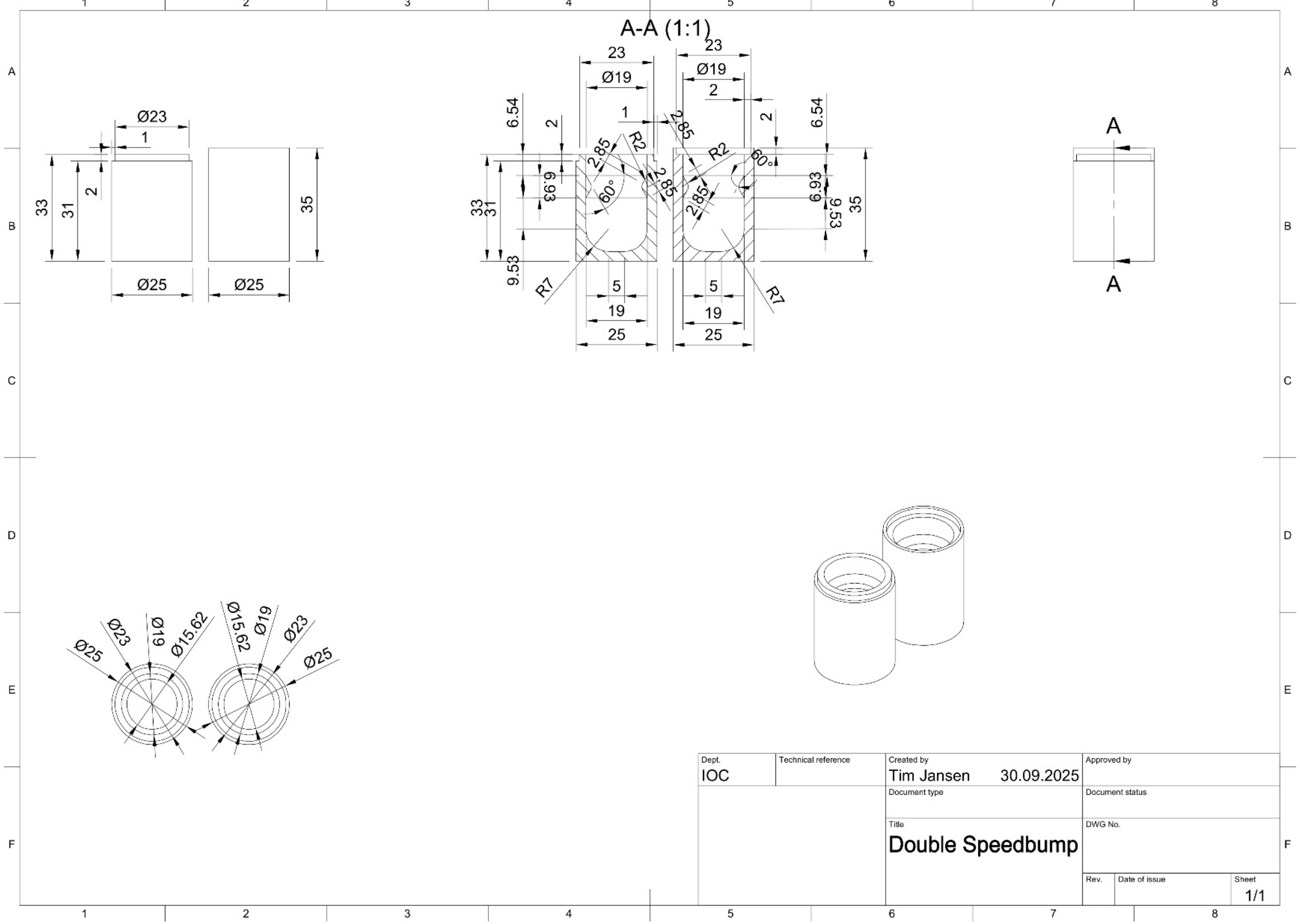

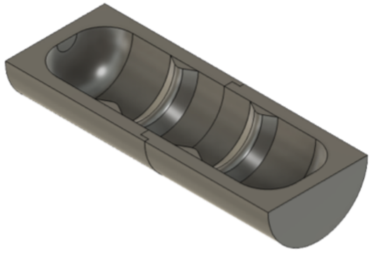

The 3D-model was prepared by Lisa Thomas.

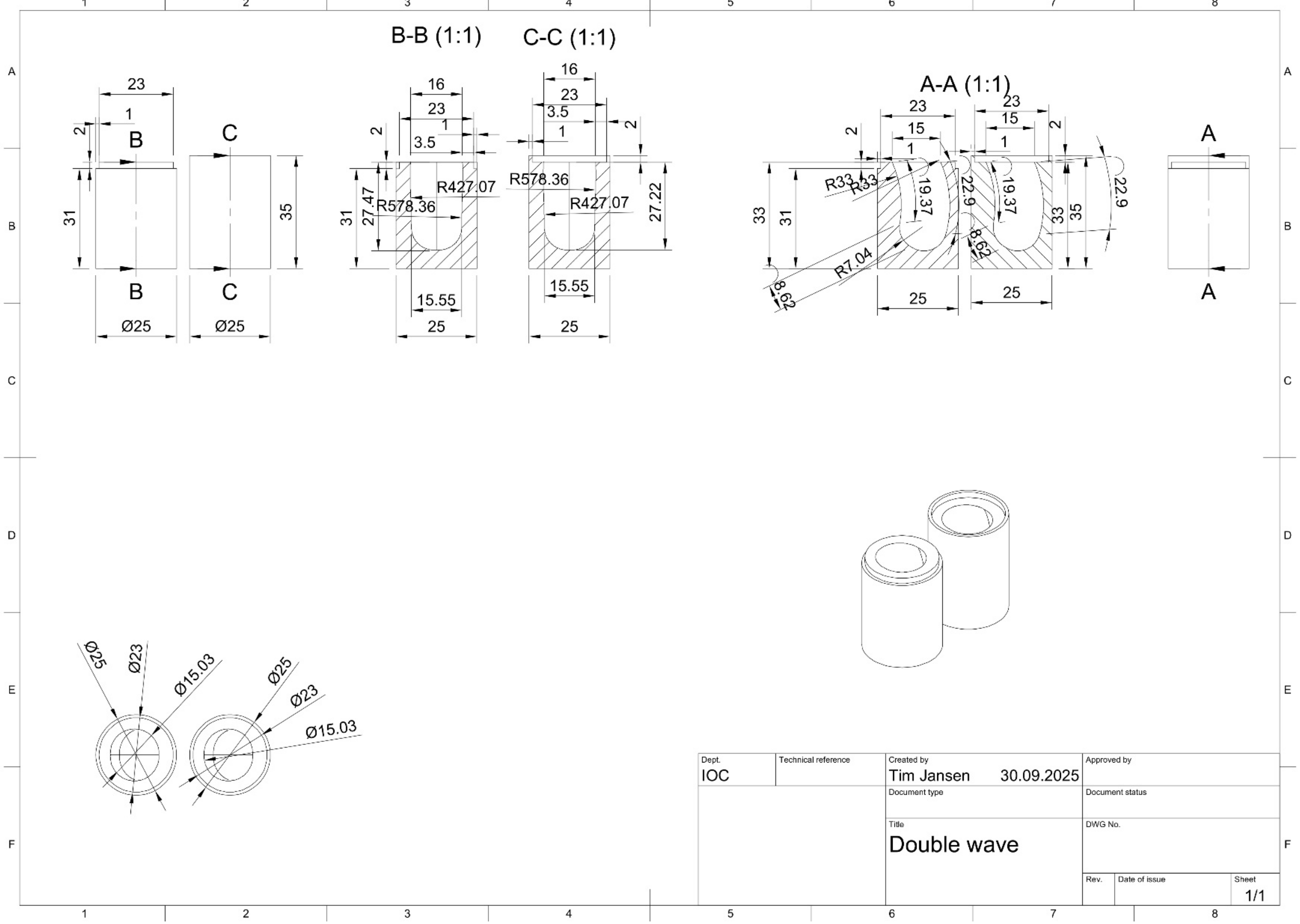

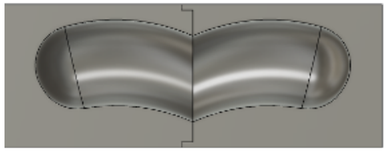

The 3D-model was prepared by Lisa Thomas.

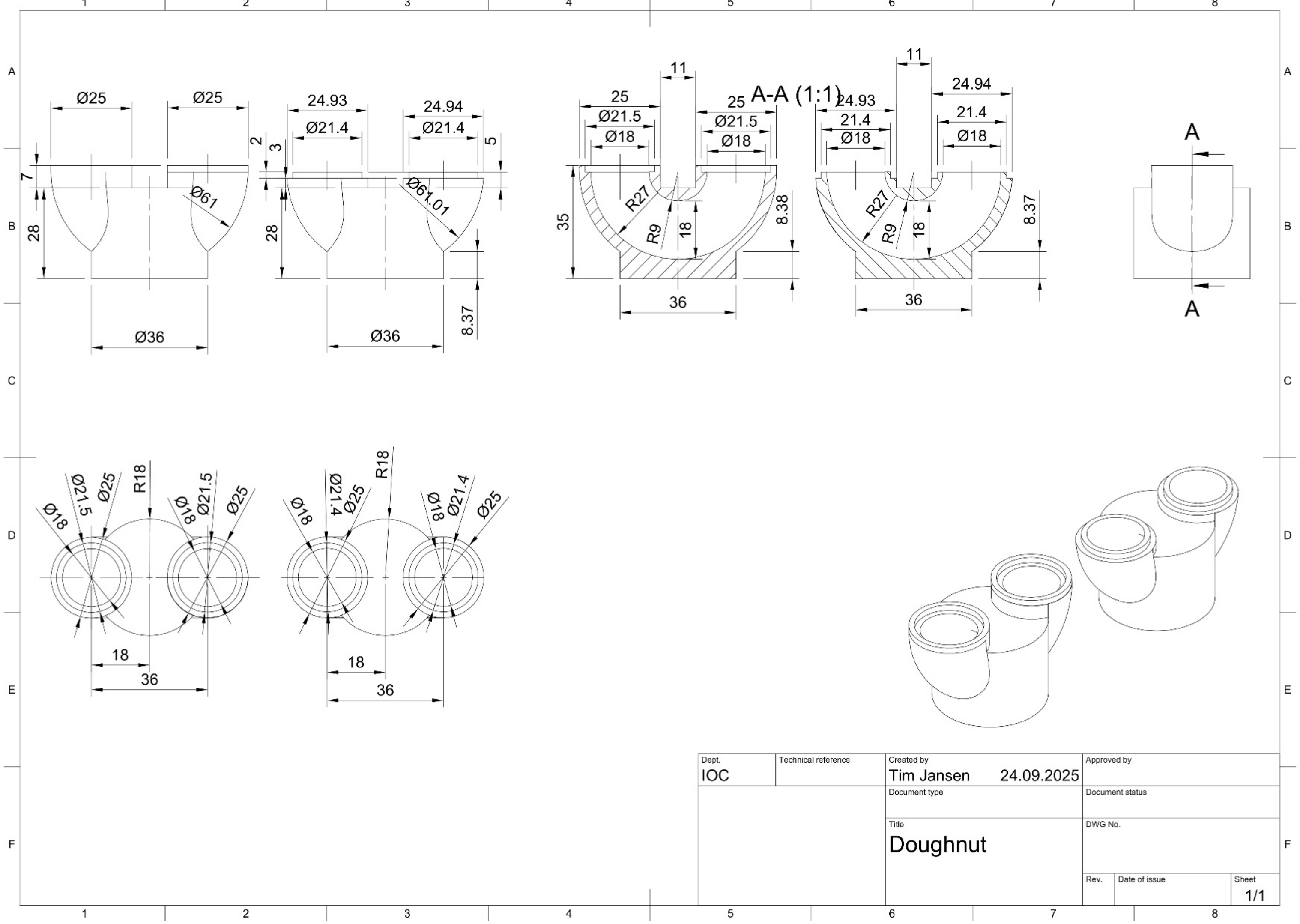

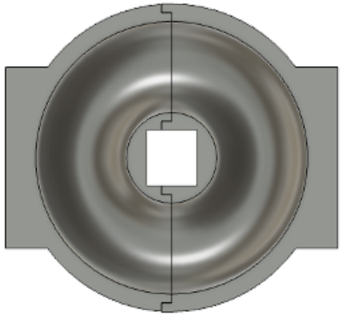

The 3D-model was prepared by Tim Jansen.

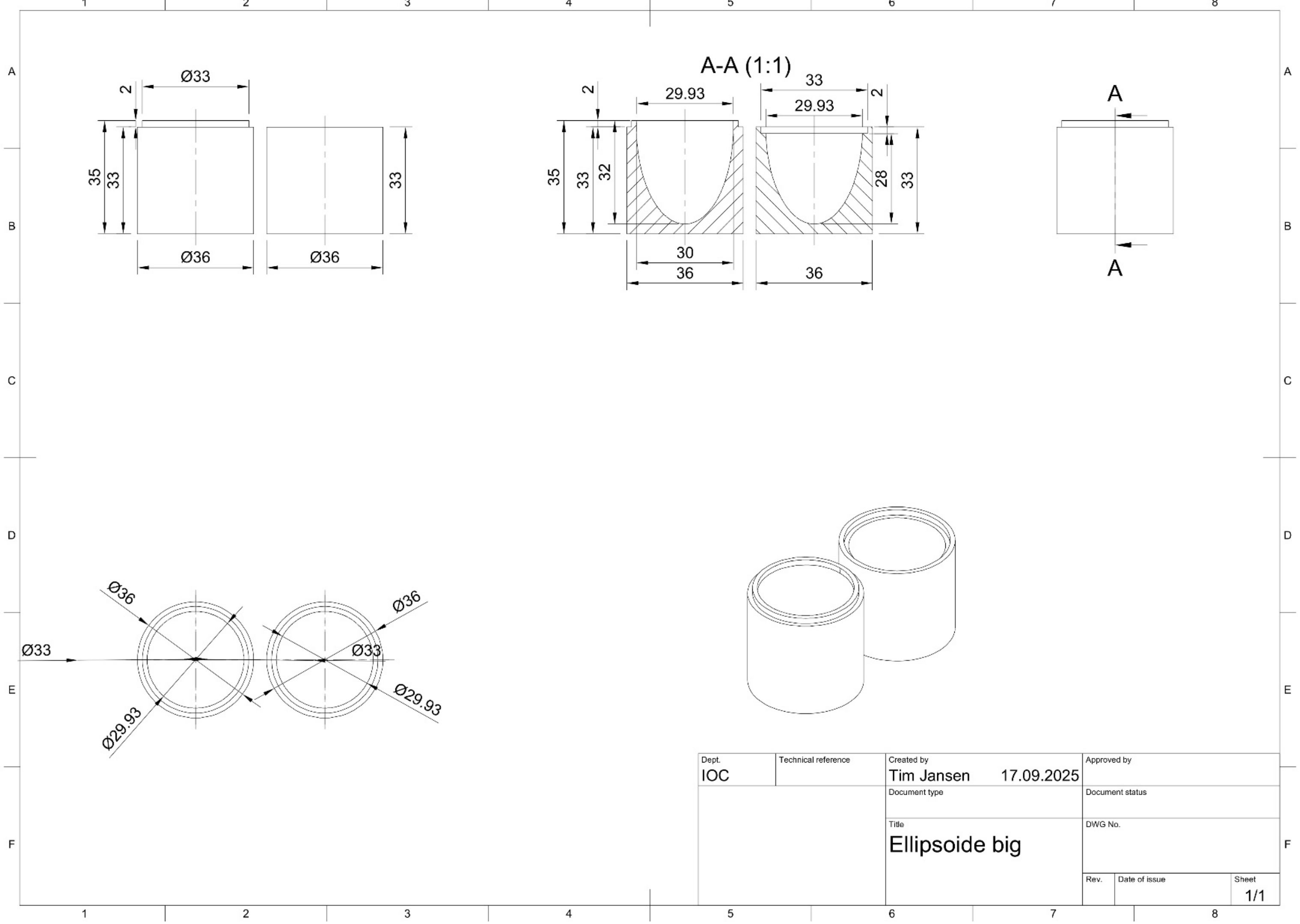

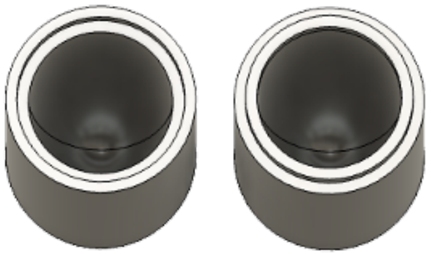

The 3D-model was prepared by Tim Jansen.

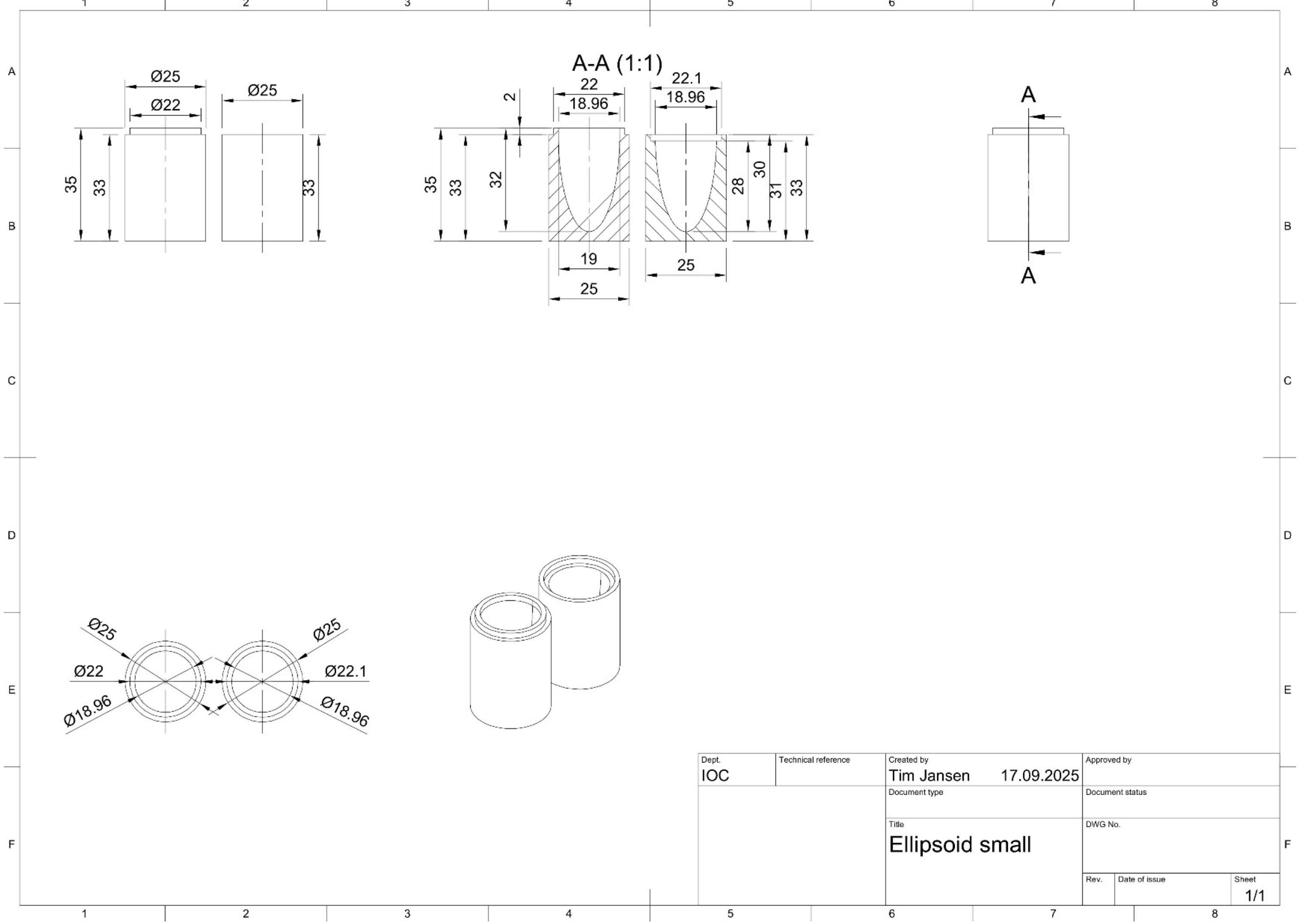

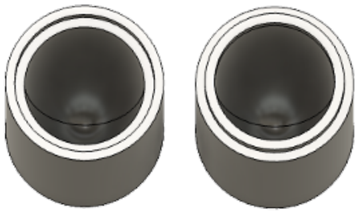

The 3D-model was prepared by Tim Jansen.

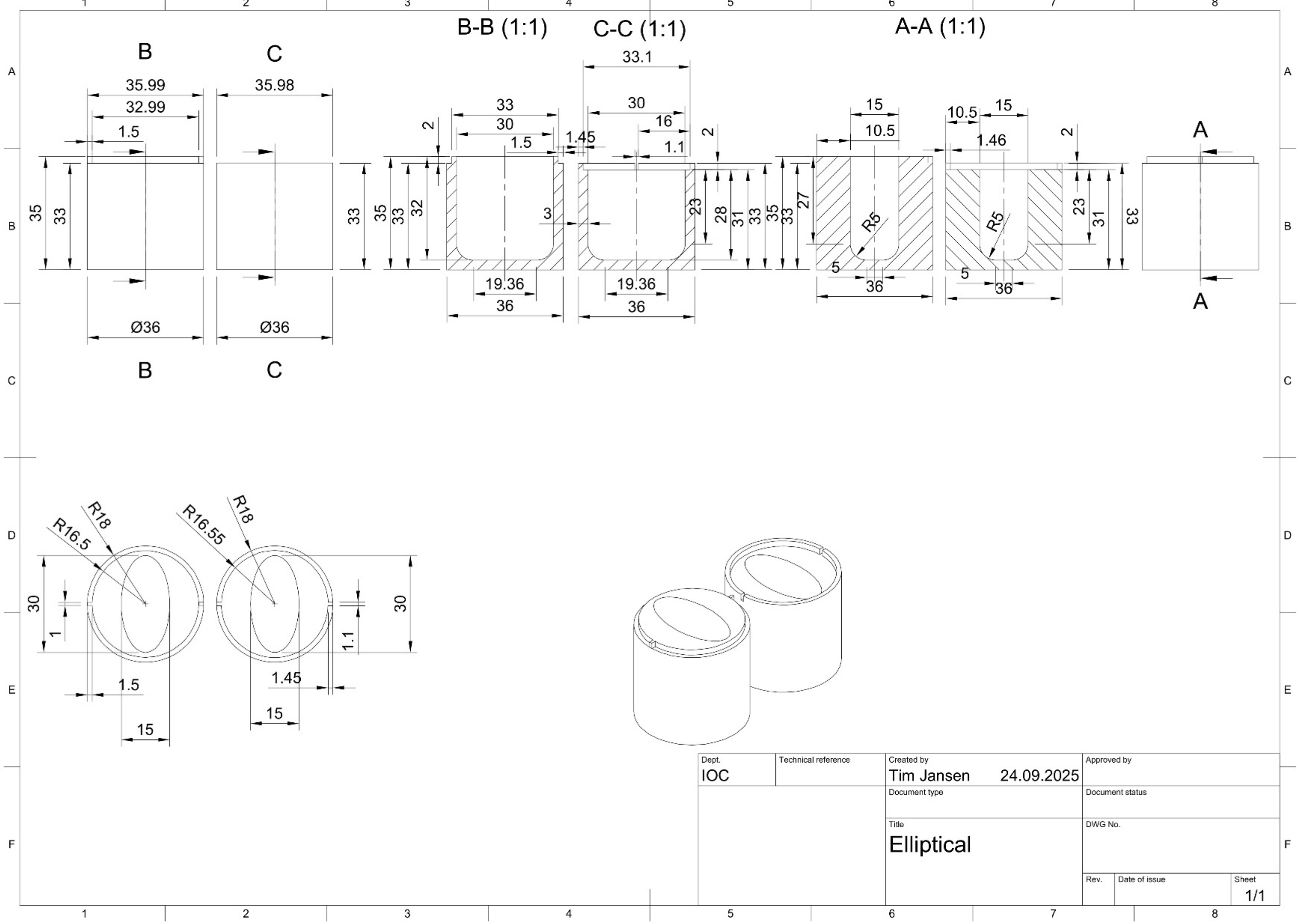

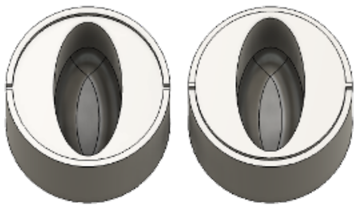

The 3D-model was prepared by Tim Jansen.

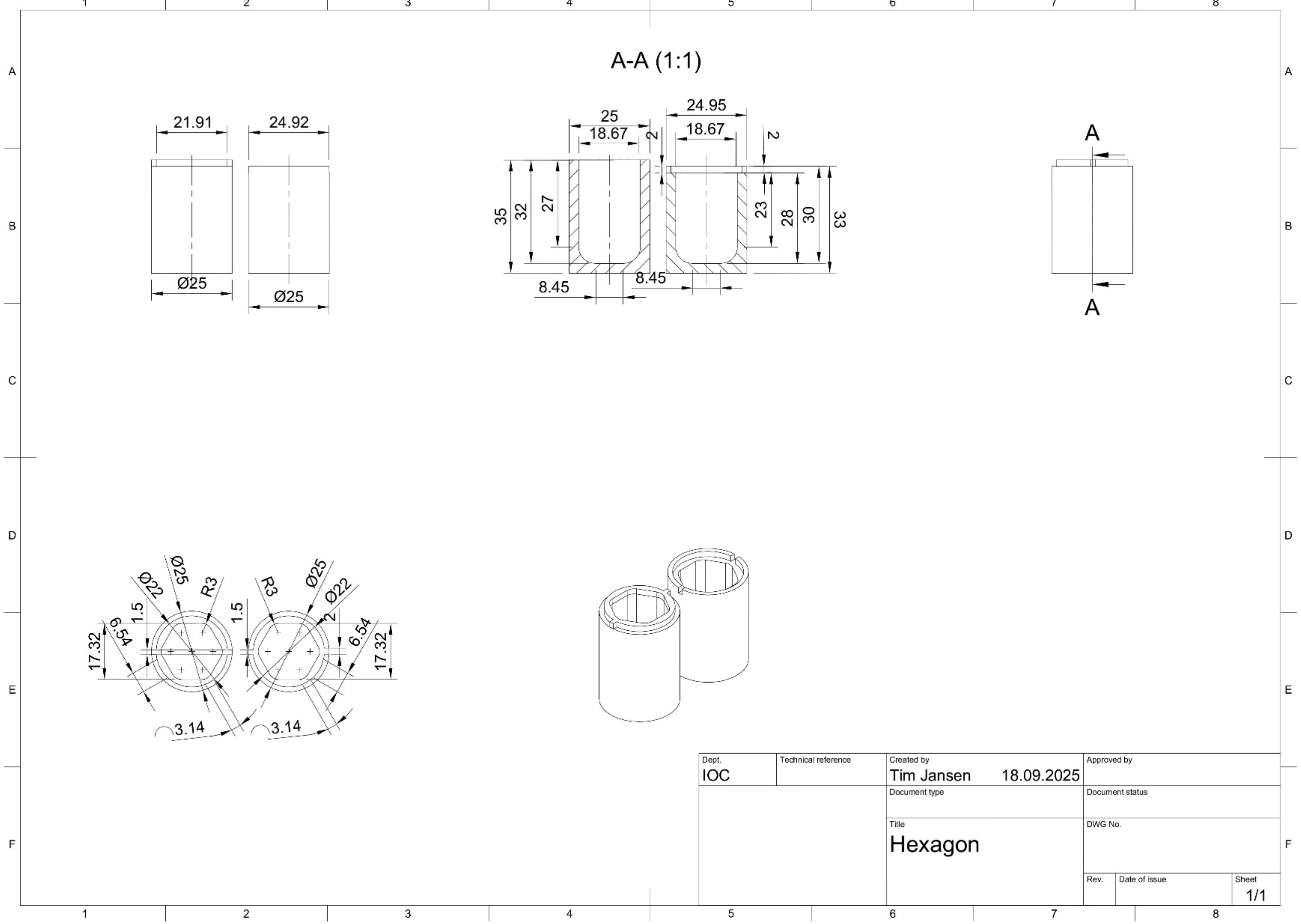

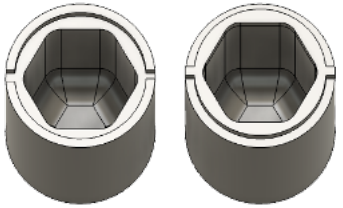

The 3D-model was prepared by Tim Jansen.

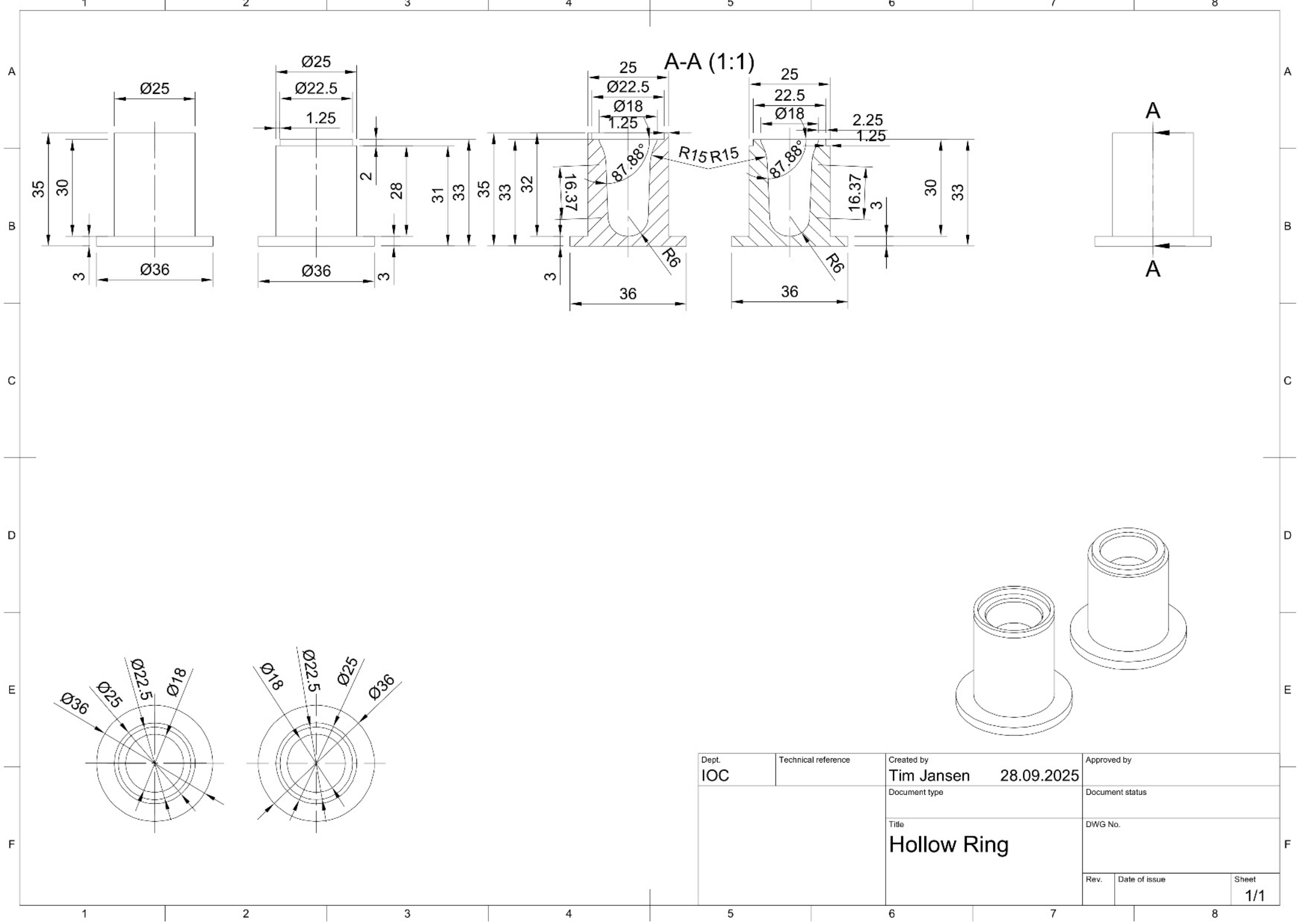

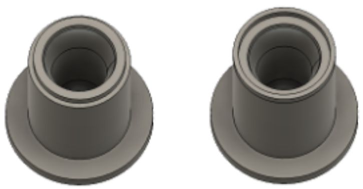

The 3D-model was prepared by Tim Jansen.

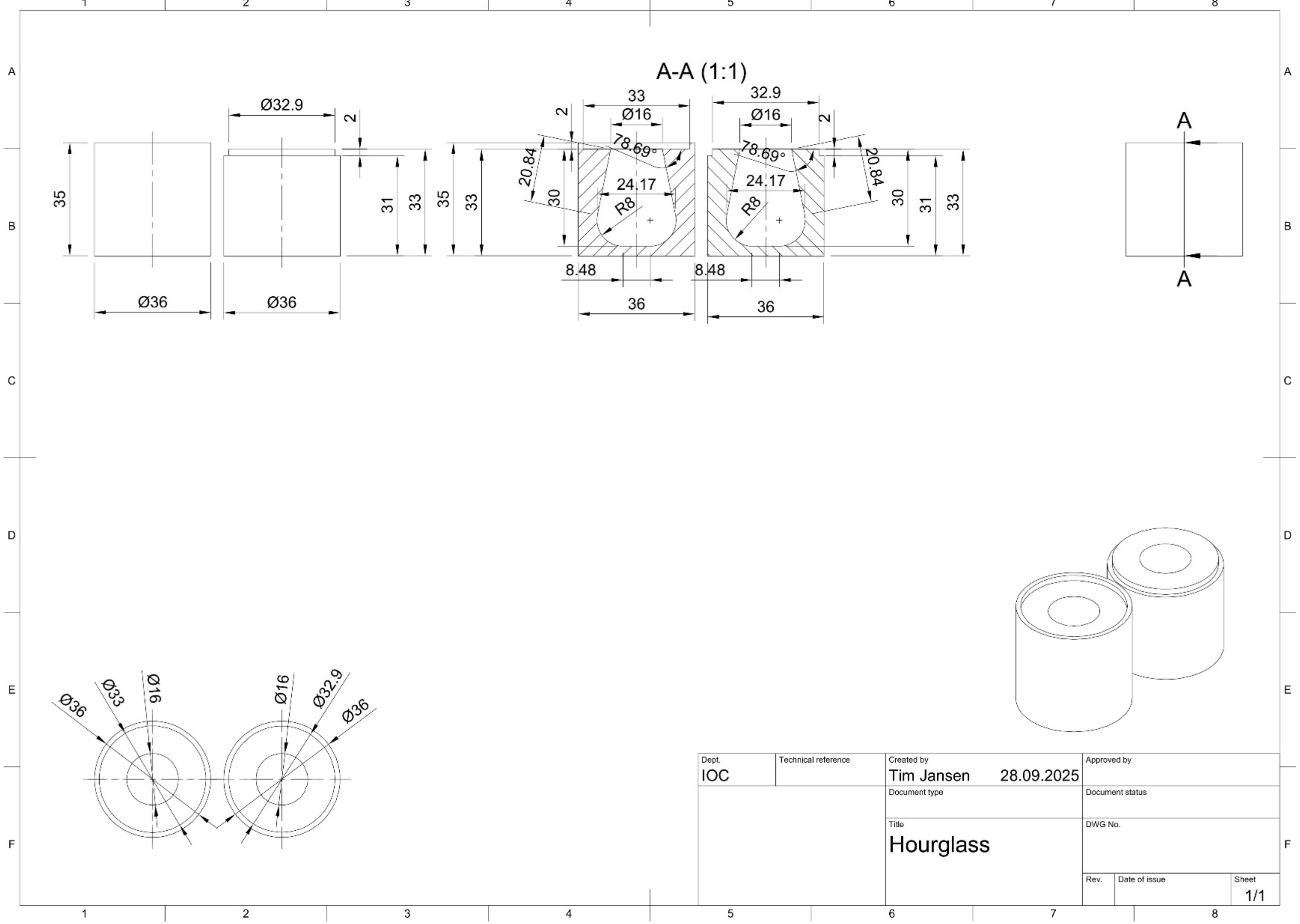

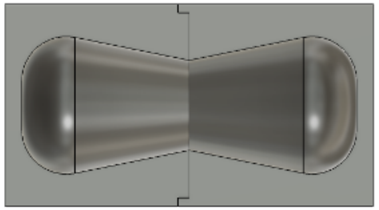

The 3D-model was prepared by Tim Jansen.

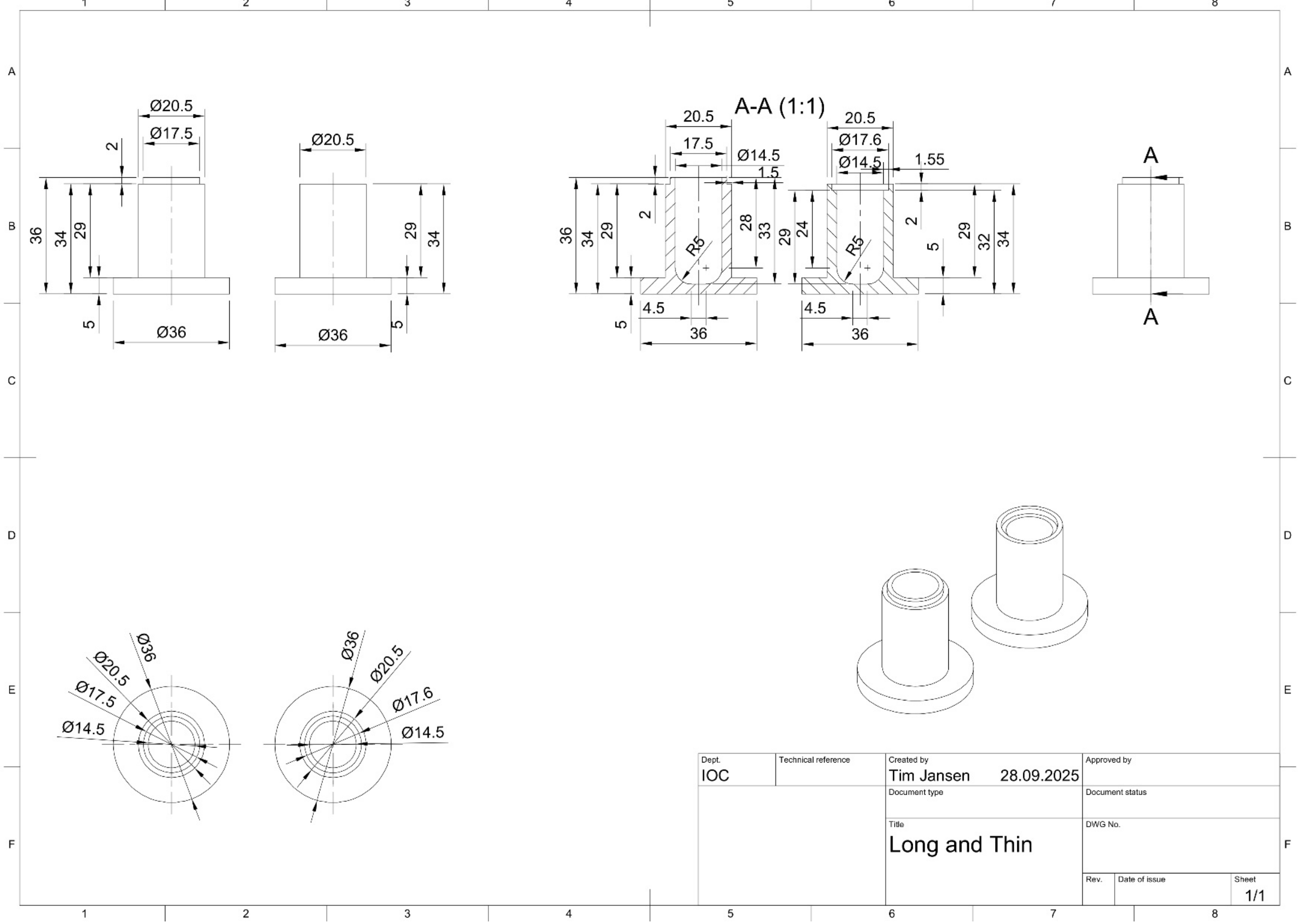

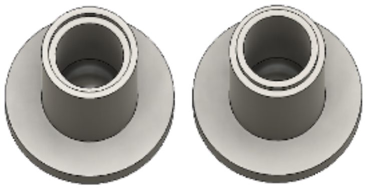

The 3D-model was prepared by Tim Jansen.

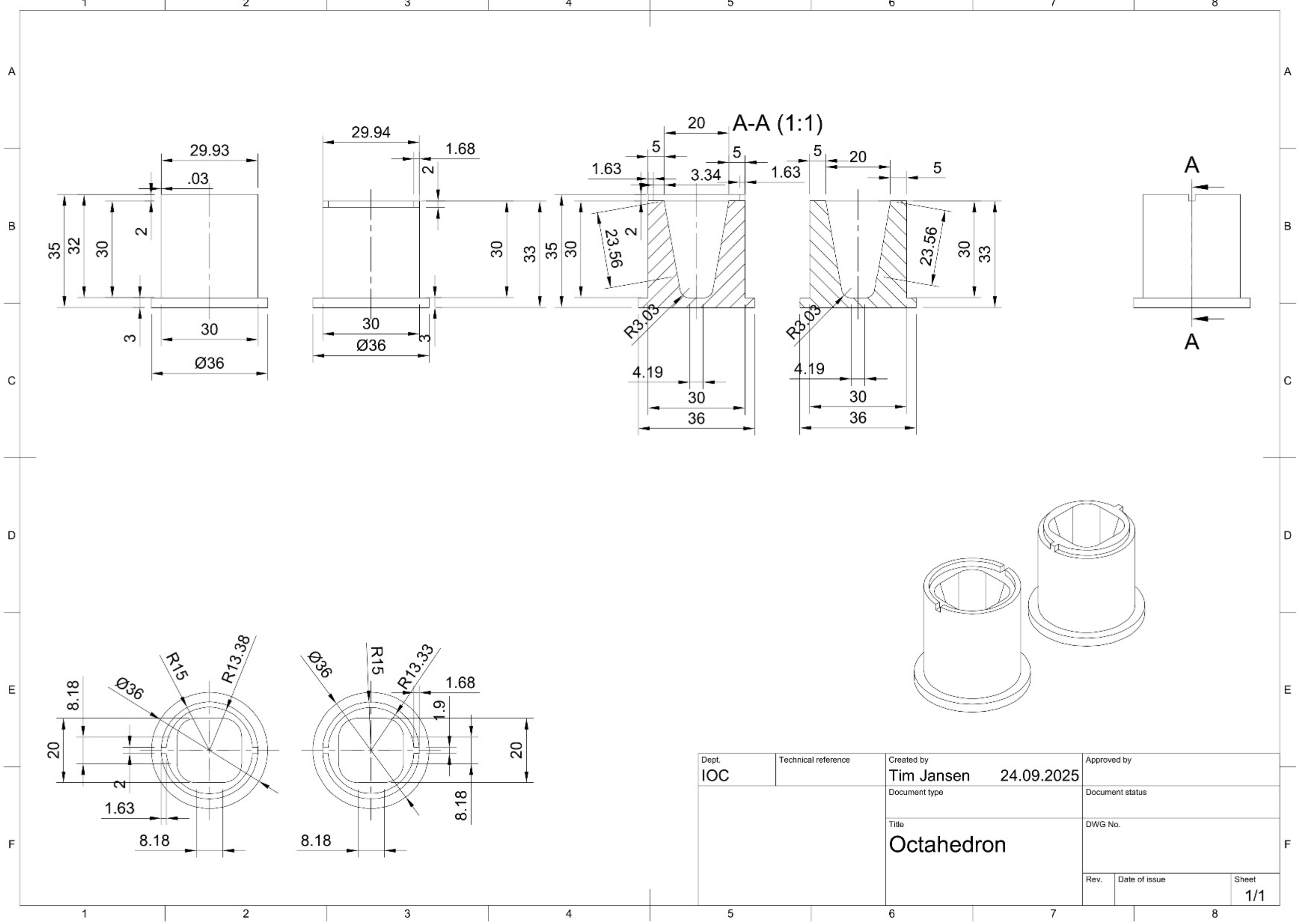

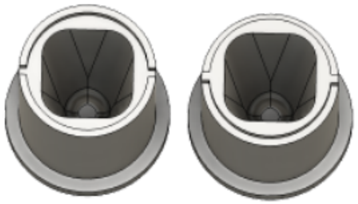

The 3D-model was prepared by Tim Jansen.

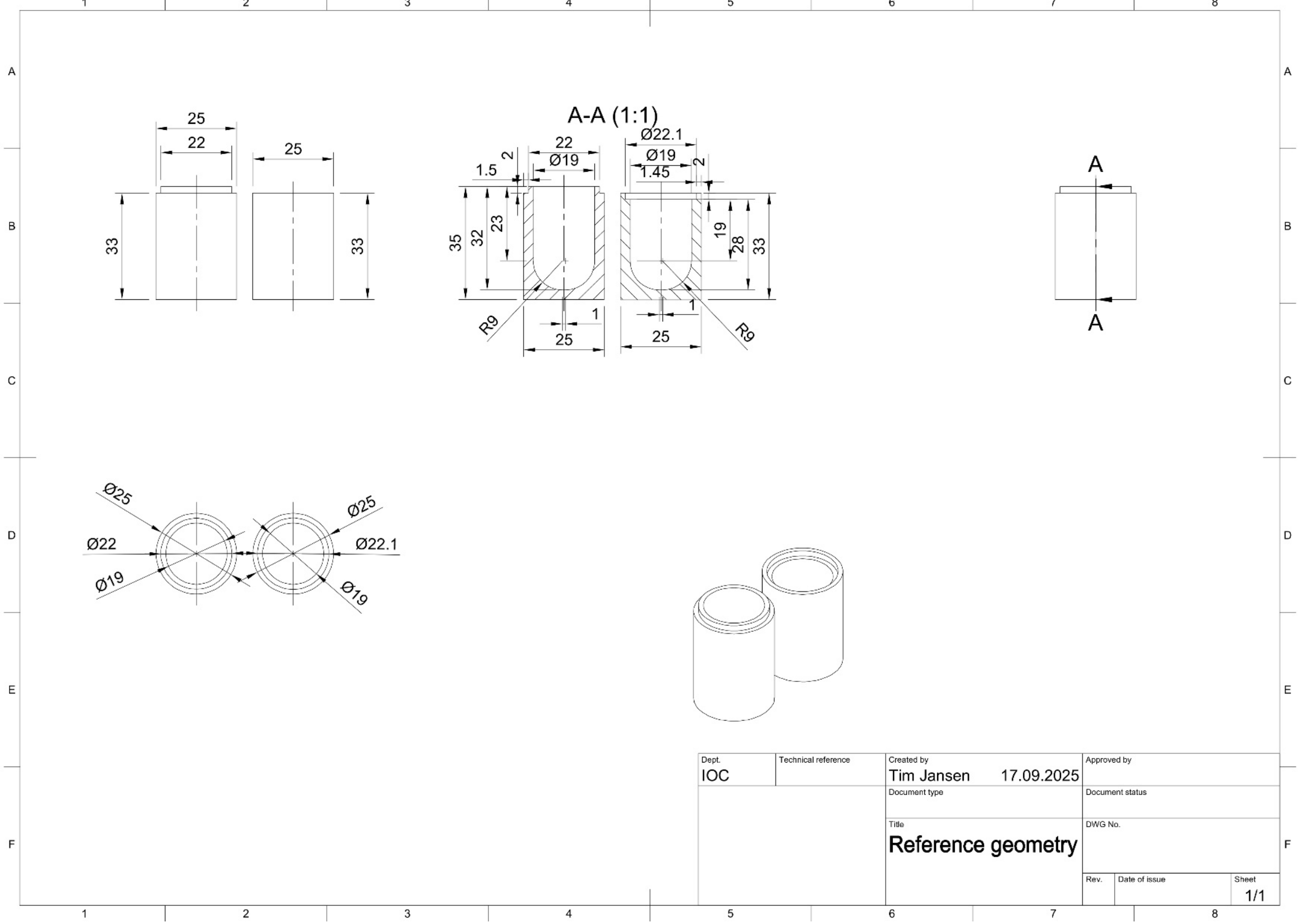

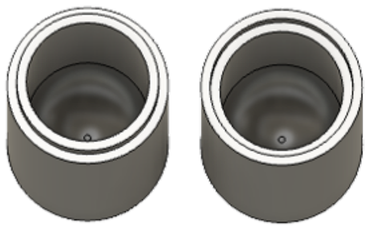

The 3D-model was prepared by Tim Jansen.

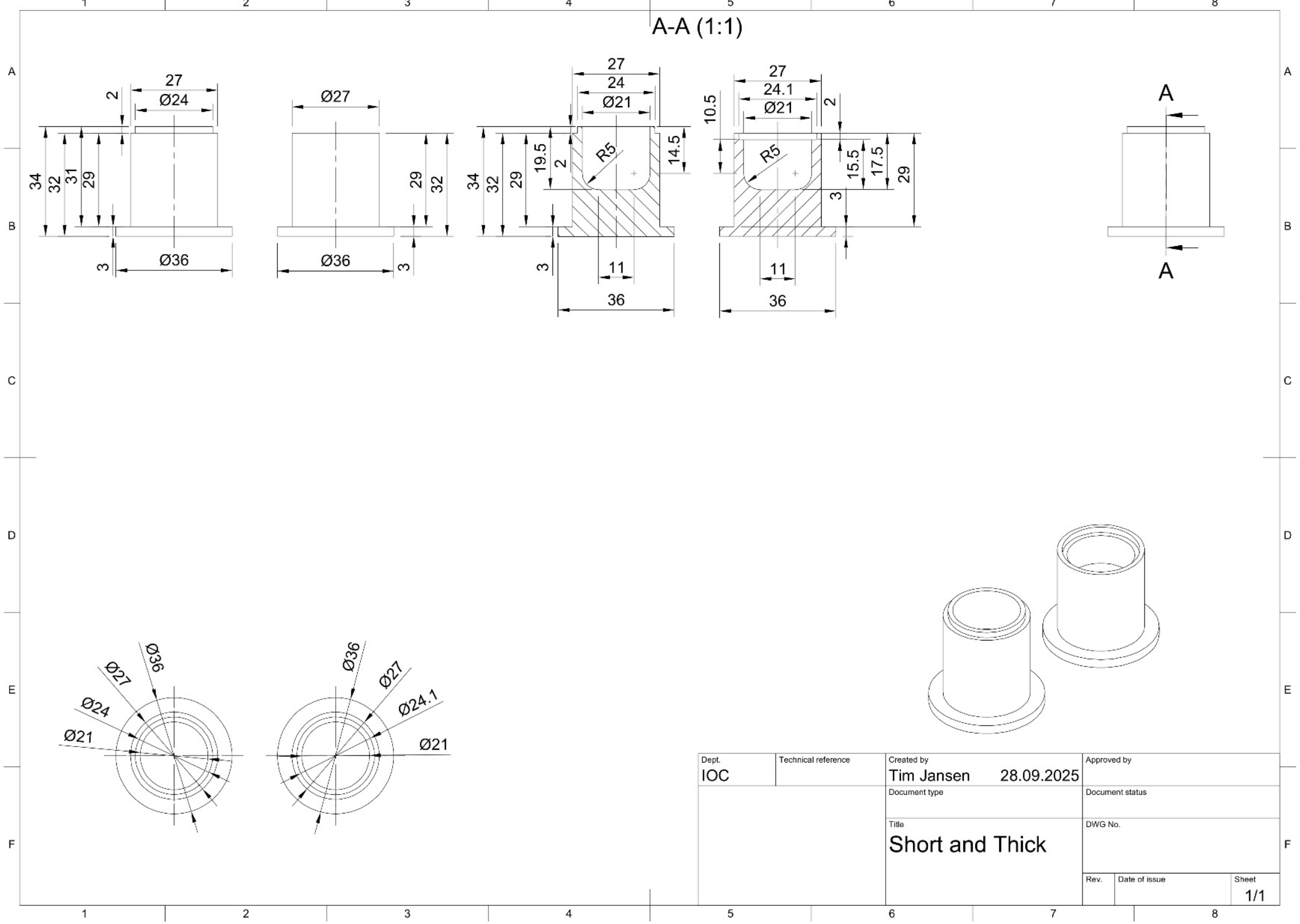

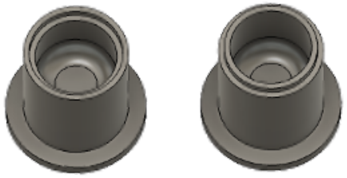

The 3D-model was prepared by Tim Jansen.

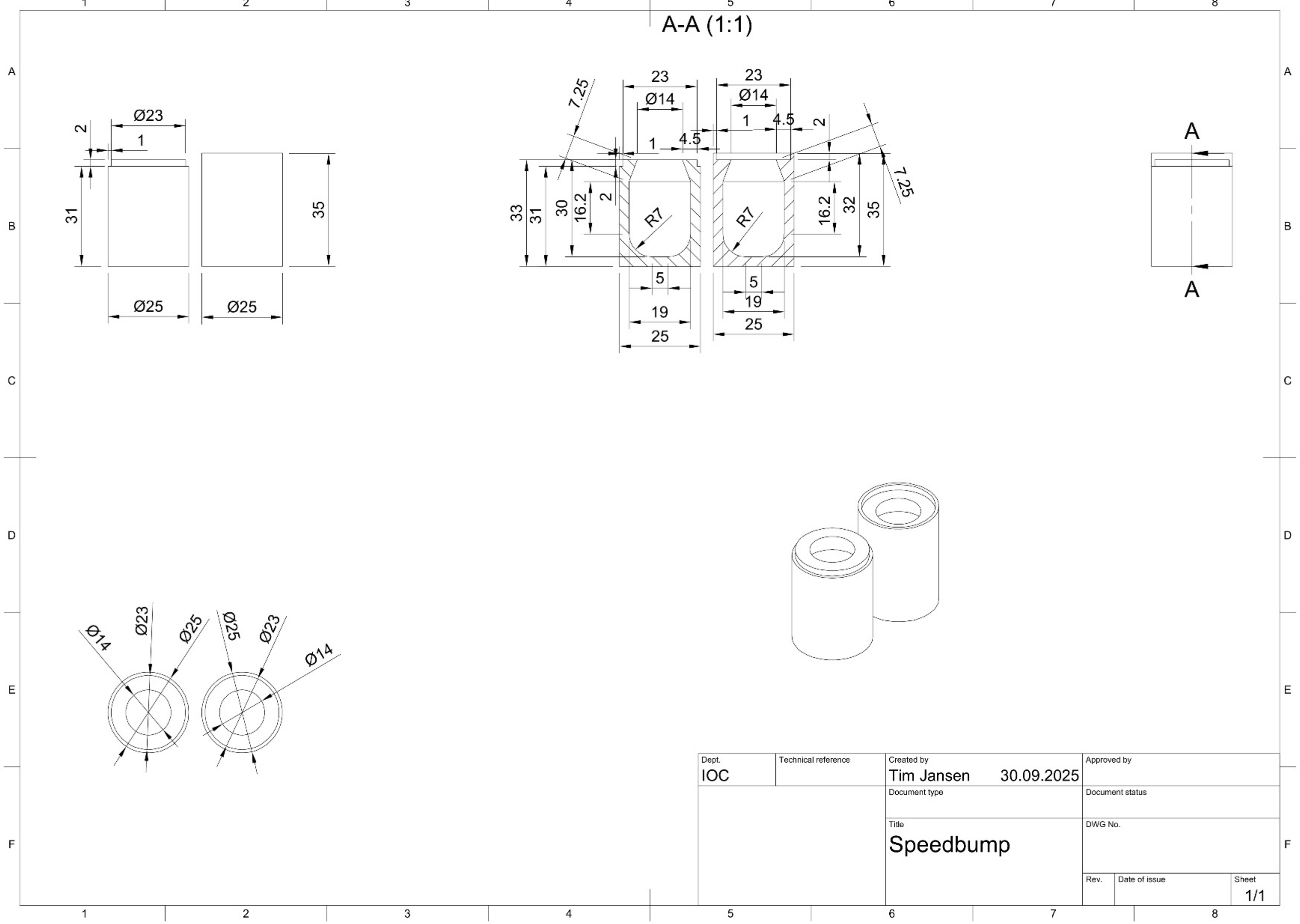

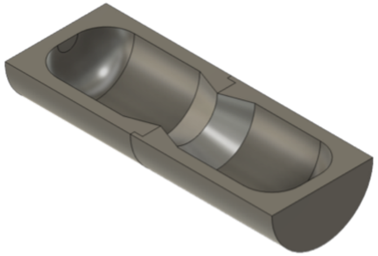

The 3D-model was prepared by Lisa Thomas.

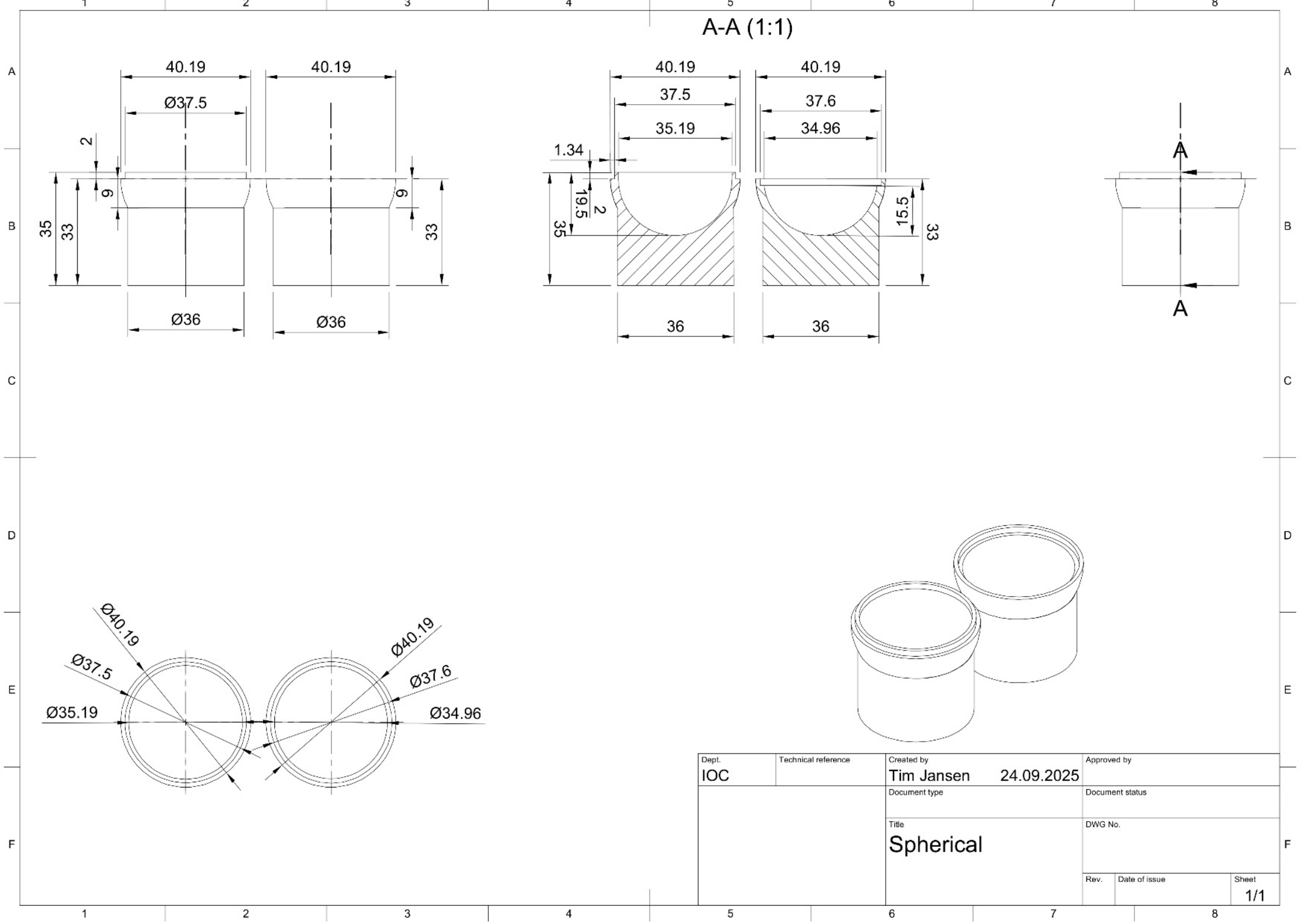

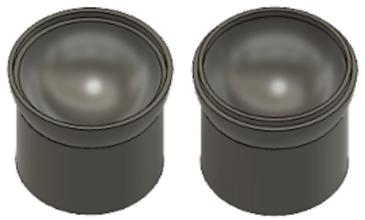

The 3D-model was prepared by Tim Jansen.

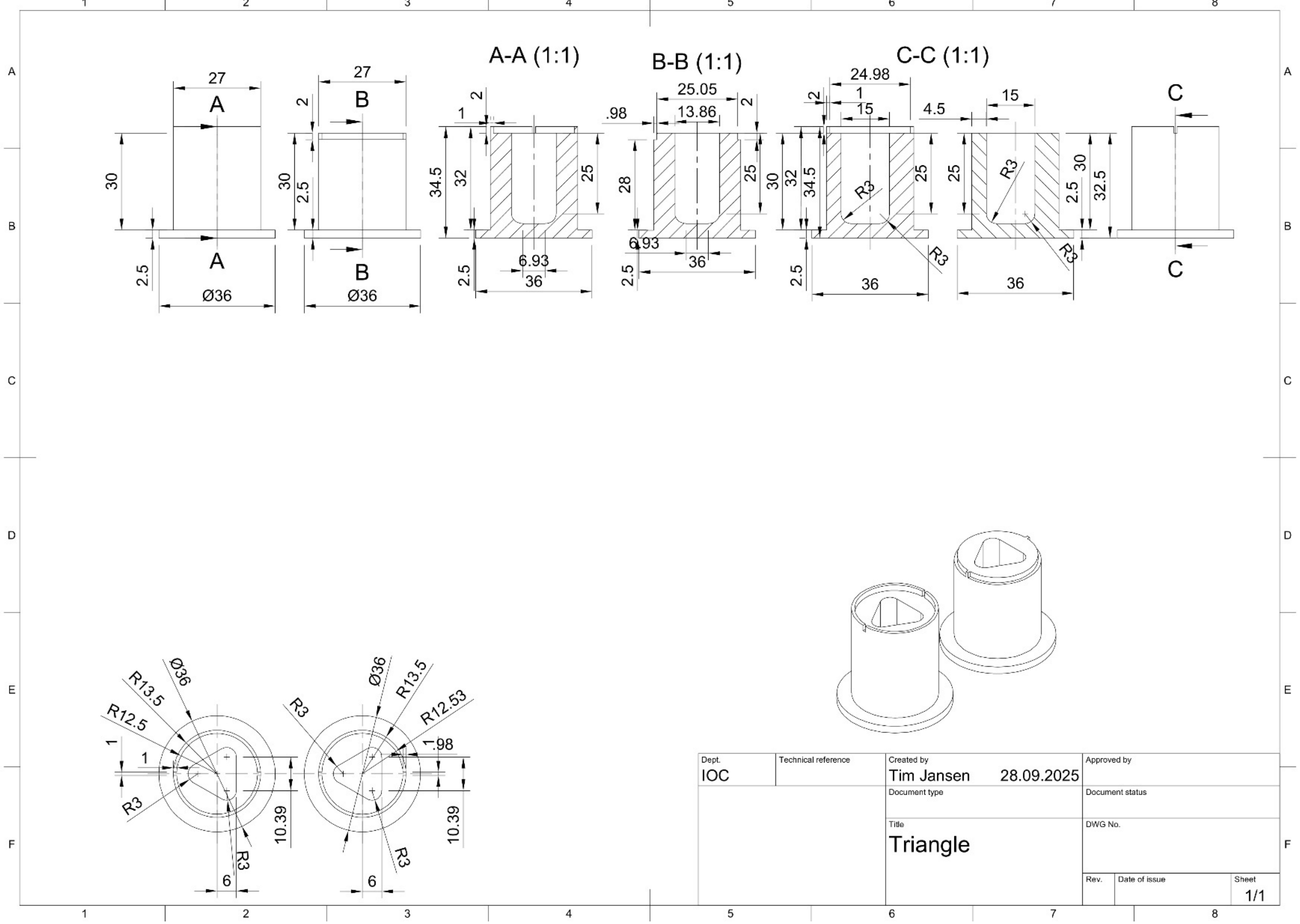

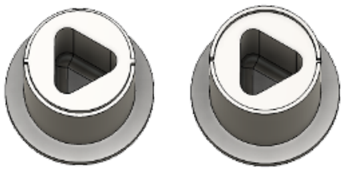

The 3D-model was prepared by Tim Jansen.

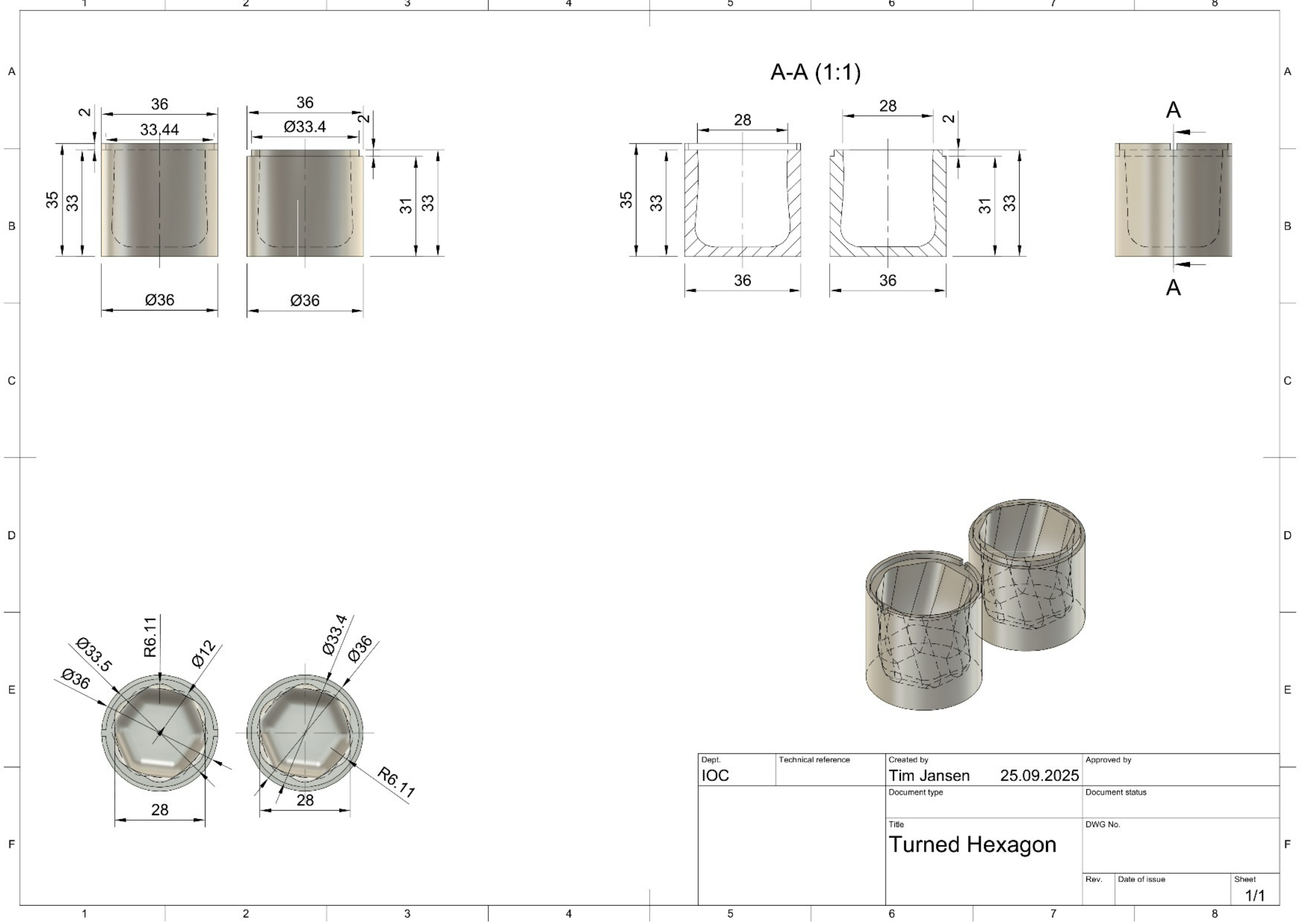

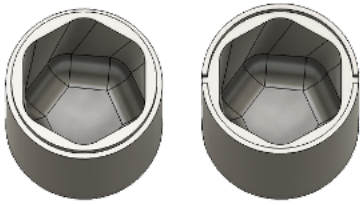

The 3D-model was prepared by Tim Jansen.

The Turned Hexagon is quite a unique shape. Thus, the technical drawing differs from the other ones. The hexagon is turned by 60° for each half so that for the entire jar an angular turning of 120° is reached which corresponds to one over six of the turning. The direction of turning is a left-handed helical structure.

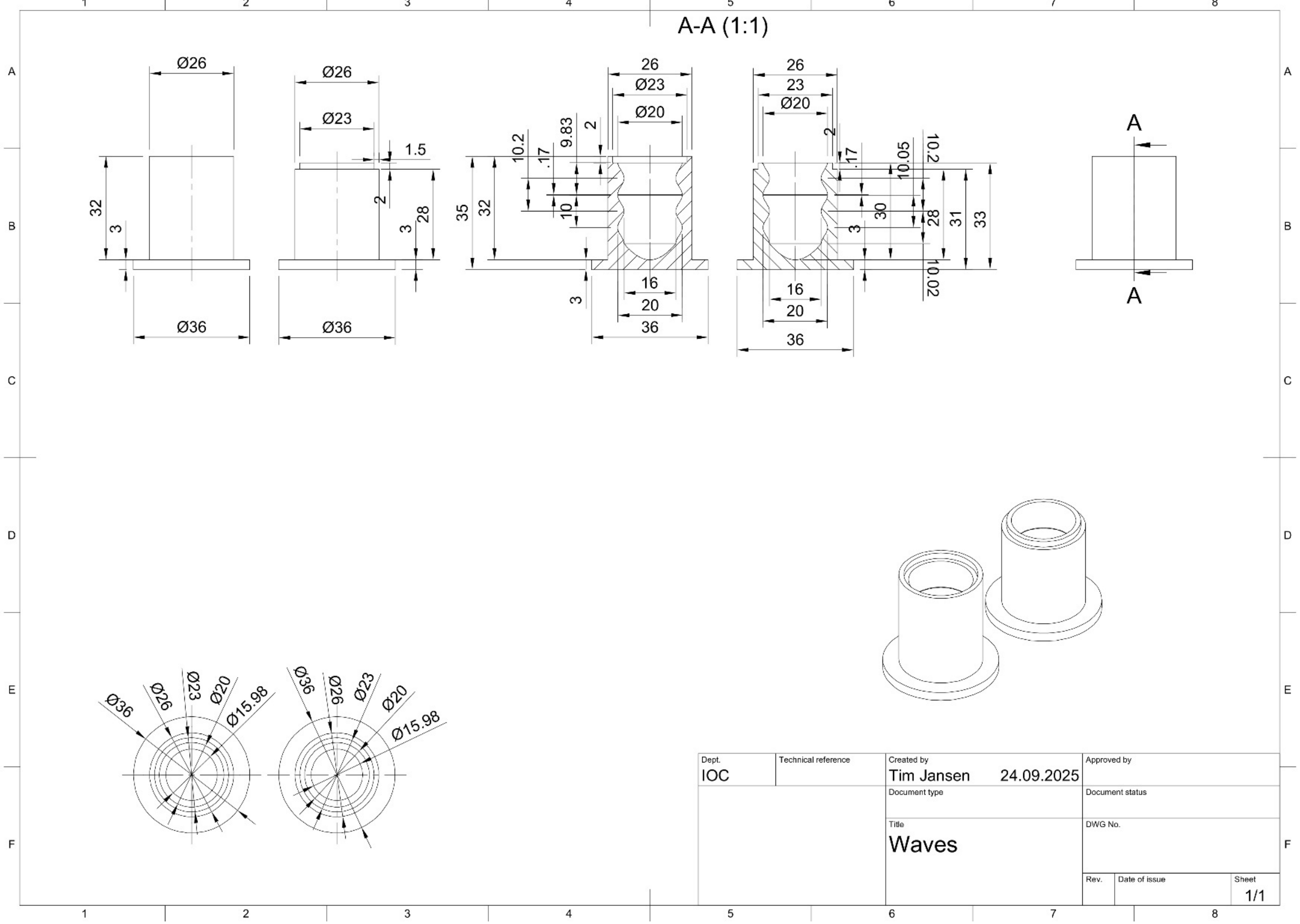

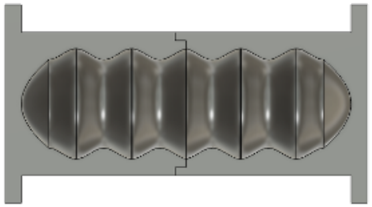

The 3D-model was prepared by Tim Jansen.

2 3D printing of the geometries

All geometries were printed on an SLA-3D printer (model: Photon Mono M5s Pro by Anycubic) using a "High Clear Resin" from Anycubic {with the following ingredients as listed in the respective safety data sheet provided by the company: oxybis(methyl-2,1-ethanediyl) diacrylate, poly(oxy-1,2-ethanediyl), α,α'-[(1-methylethylidene)di-4,1-phenylene]bis[ω-[(1-oxo-2-propen-1-yl)oxy], (octahydro-4,7-methano-1*H*-indenediyl)bis(methylene) diacrylate and bisphenol A polyethyenglycol diether dimethacrylate}. The printing files were obtained by the software Chitubox basic, where the .stl files of the geometries were transcribed. The printing settings were adjusted from the given settings to better fit the printer and resin. The used settings for the High Clear Resin are shown in Table 1:

Table 1. Used printing settings for the High Clear Resin as used in Chitubox Basic software.

Layer Height	0.05 mm	
Bottom Layer Count	3	
Exposure Time	3.6 s	
Bottom Exposure Time	22 s	
Transition Layer Count	6	
Transition Type	Linear	
Light-off Delay	0.500 s	
Bottom Lifting Distance	4 mm	4 mm
Lifting Distance	4 mm	4 mm
Bottom Lift Speed	360 mm/min	360 mm/min
Lifting Speed	360 mm/min	360 mm/min
Bottom Retract Speed	360 mm/min	360 mm/min
Retract Speed	360 mm/min	360 mm/min

After printing, the obtained geometries were washed with isopropanol and acetone, dried in an airflow, and cured in the Anycubic curing station with UV-light of a wavelength of 405 nm for 3 minutes.

3 Synthesis of copper(I) thiocyanate bipyridine triphenylphosphine

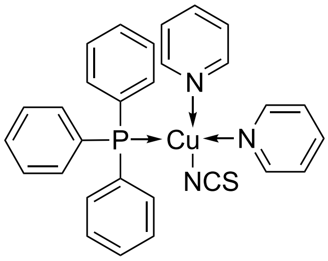

The synthesis of copper(I) thiocyanate bipyridine triphenylphosphine was conducted as described in the literature.[S1] Copper(I)thiocyanate (2.00 g, 16.3 mmol, 1.00 equiv.) and triphenylphosphine (4.31 g, 16.3 mmol, 1.00 equiv.) were dissolved in pyridine (50.0 mL, 618 mmol). The mixture was heated to 70 °C for 3 h. Then, the pyridine was removed by distillation to provide the solid complex. The complex was recrystallized from pyridine (15 mL) and then washed with ice-cold pyridine (10 mL). The crystalline complex was dried in vacuum and stored at room temperature.

NMR: **^{1}H-NMR (400 MHz, $CDCl_3$-*d*):** δ = 8.72 (s, 4H, H_{arom}), 7.69 (t, J = 7,7 Hz, 2H, H_{arom}), 7.42-7.29 (m, 12H, H_{arom}), 7.29-7.22 (m, 7H, H_{arom}). **^{13}C{^{1}H}-NMR (101 MHz, $CDCl_3$-*d*):** δ = 136.1 (SCN), 133.9 (d, J = 15.1 Hz, C_{arom}),133.1 (d, J = 30.7 Hz, C_{arom}), 129.9 C_{arom}), 128.7 (d, J = 9.2 Hz, C_{arom}), 124.3 (C_{arom}).

IR: **ATR:** ν = 2061 (s), 1594 (m), 1478 (m), 1436 (s), 749 (vs) cm^{-1}.

CHN: Calcd. for ($C_{29}H_{25}CuN_3PS$): C: 64.25%, H: 4.65%, N: 7.75%; found: C: 64.32%, H: 4.65%, N: 7.64%.

4 Experiments to obtain the hit maps

The experiments were performed using an Insolido IST636 mixer mill at different frequencies. The photography and videography were done by a Samsung Galaxy S20FE mobile phone. The long exposure pictures were taken with the photography pro mode with ISO 50 and two seconds of exposure time. The high-speed footage was recorded with the super slow-mo feature of the Samsung phone, which is capable of shooting videos with a frame rate of 960 fps and a duration of half a second. The red flashlight was a LingsFire headlamp with red light feature. The red light has a non-defined wavelength. For the videos, the experimental vessel containing 100 mg of the triboluminescent crystals and a 10 mm PTFE ball was placed into the left arm of the ball mill. The red light was placed on top of the lid, and the camera was fixed in front with a tripod. The ball mill was started and, when the mill reached the desired

frequency, the video was started. After half a second of filming, the ball mill was turned off. Subsequently, the filming process was repeated. After every second video the vessel was removed, washed, and new triboluminescent crystals were loaded. With the newly loaded crystals two additional videos were made. The process was repeated a third time to get a total of six videos, leading to three seconds of filmed time. Then, the videos were transferred to a computer where the frame was cut to only show the vessel and the mirrored image. The videos were analyzed frame by frame, and every hit was manually marked on an excel sheet with a distinct pattern which resembled the geometry of the specific vessel. When over time more hits were recorded on a particular spot, the new hit was added to the existing data. Larger light reflexes lead to more parcels counted as hits in comparison to small light reflexes. This results in a better estimation of the overall energy input due to the incorporation of hit size rather than just counting each light reflex as one singular hit.

5 Hit maps and hit counts of every geometry

In the following, the hit maps of every geometry are shown. Additionally, the hit counts are given in Table 2. The geometries are sorted in alphabetical order by the given name.

Banana Orientation 1:

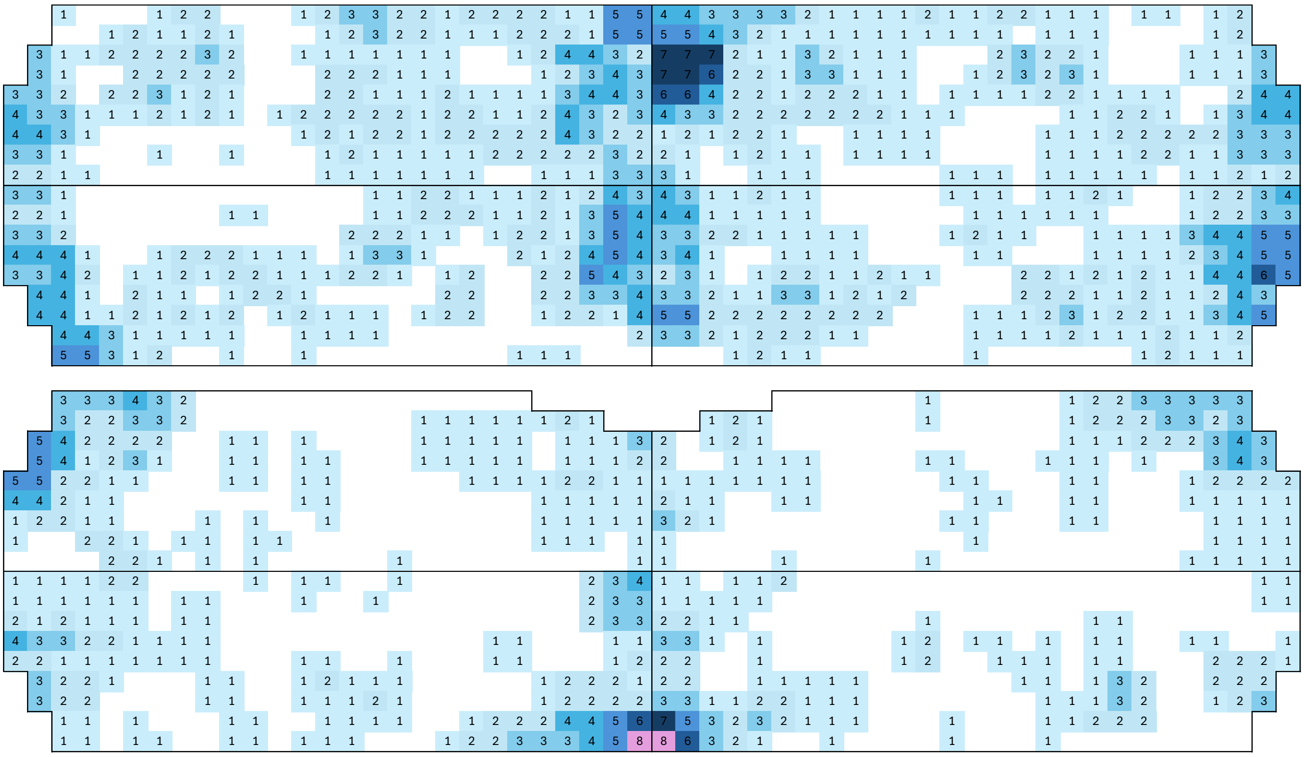

Figure 1. Hit maps of the Geometry Banana Orientation 1 at 25 Hz. Top: Front view; Bottom: Bottom view. The numbers shown are the hit counts within three seconds of milling and footage time. Each number is equally represented by its own color.

Banana Orientation 2:

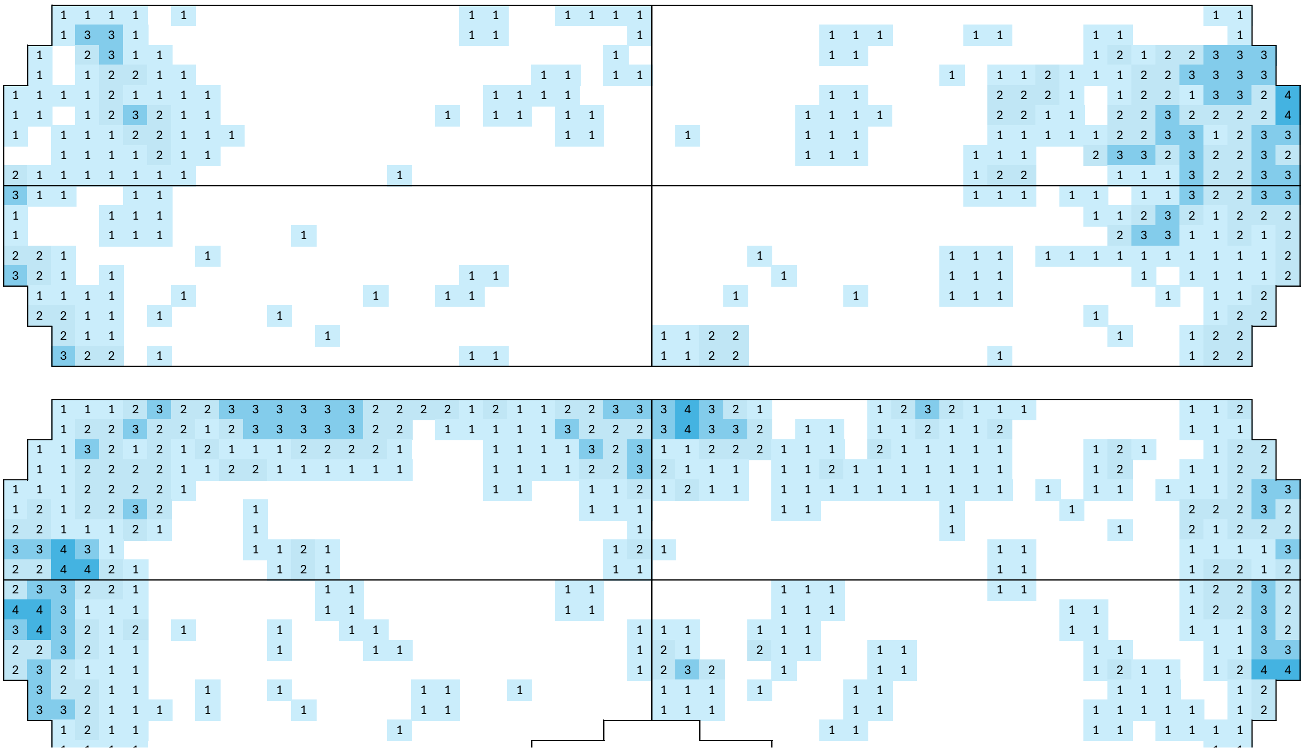

Figure 2. Hit maps of the geometry Banana Orientation 2 at 25 Hz. Top: Front view; Bottom: Bottom view. The numbers shown are the hit counts within three seconds of milling and footage time. Each number is equally represented by its own color.

Double Speedbump

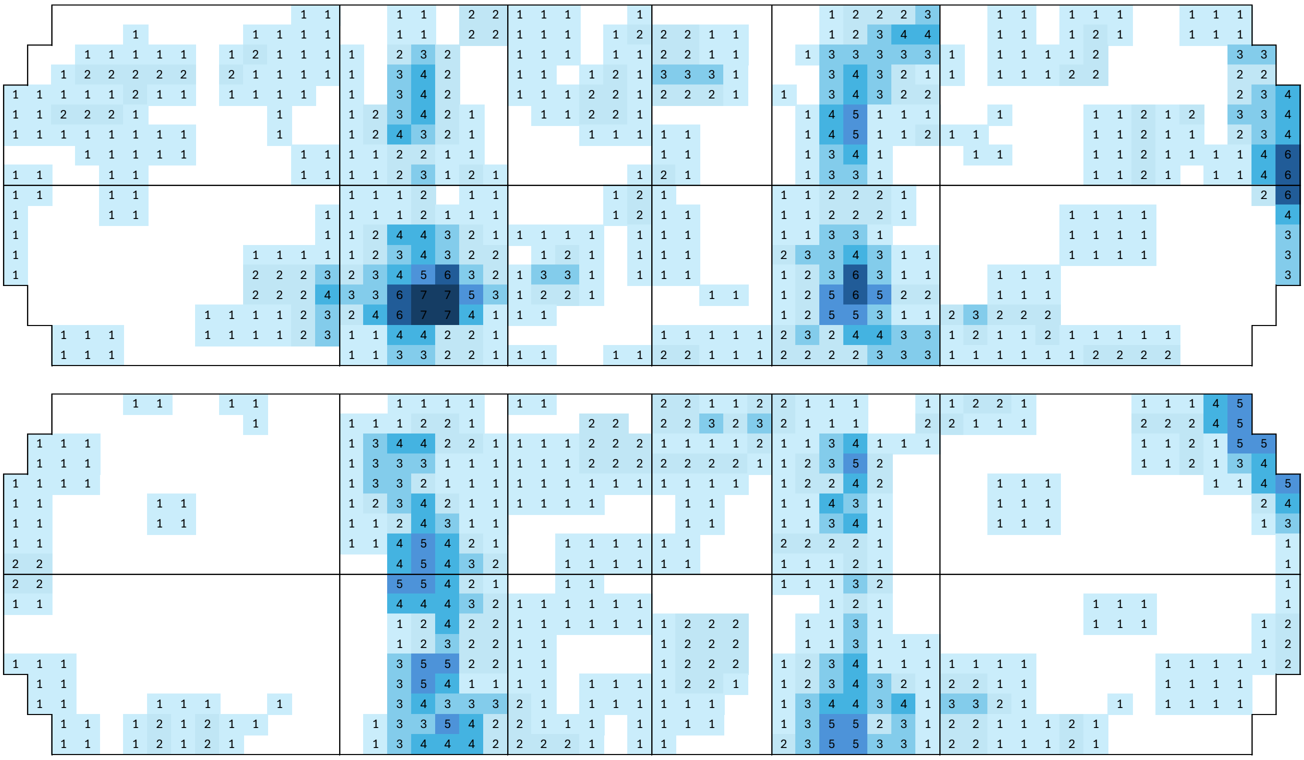

Figure 3. Hit maps of the geometry Double Speedbump at 25 Hz. Top: Front view; Bottom: Bottom view. The numbers shown are the hit counts within three seconds of milling and footage time. Each number is equally represented by its own color.

Double Wave:

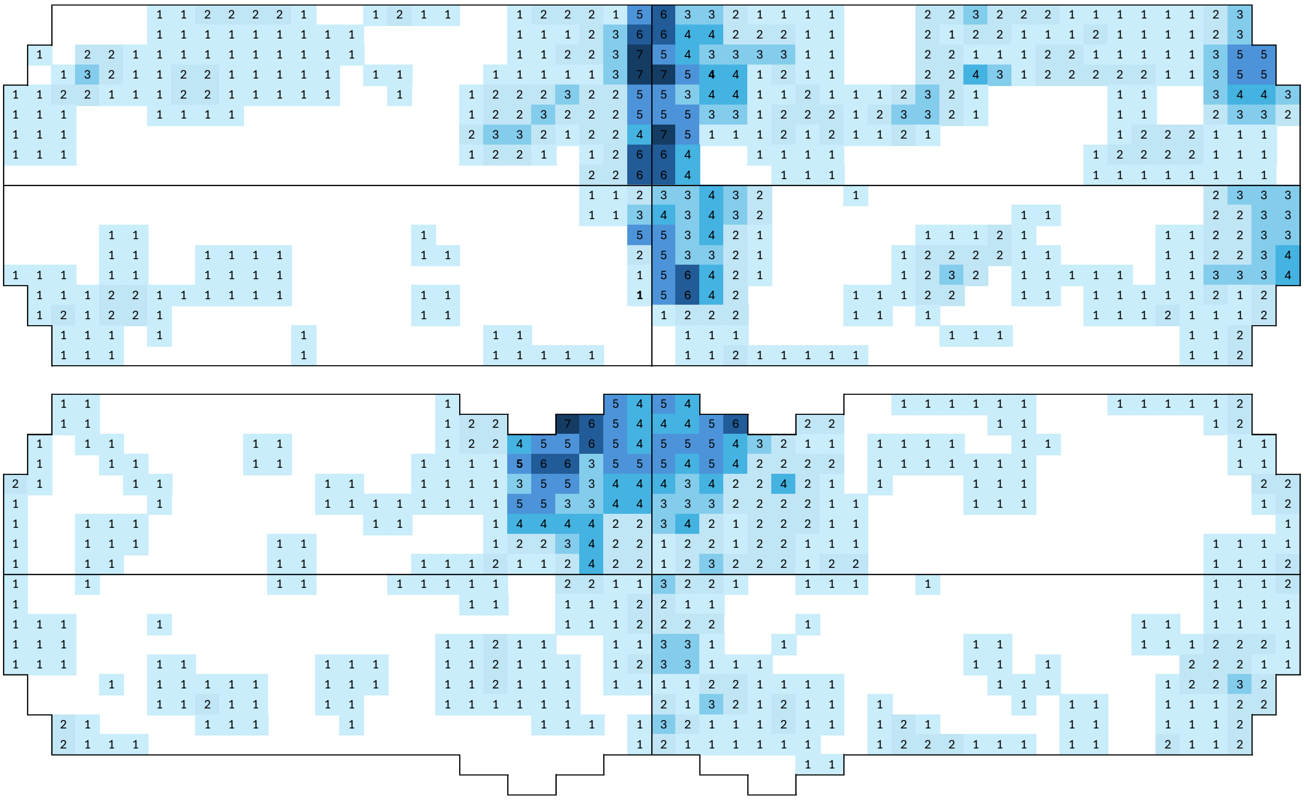

Figure 4. Hit maps of the geometry Double Wave at 25 Hz. Top: Front view; Bottom: Bottom view. The numbers shown are the hit counts within three seconds of milling and footage time. Each number is equally represented by its own color.

Doughnut

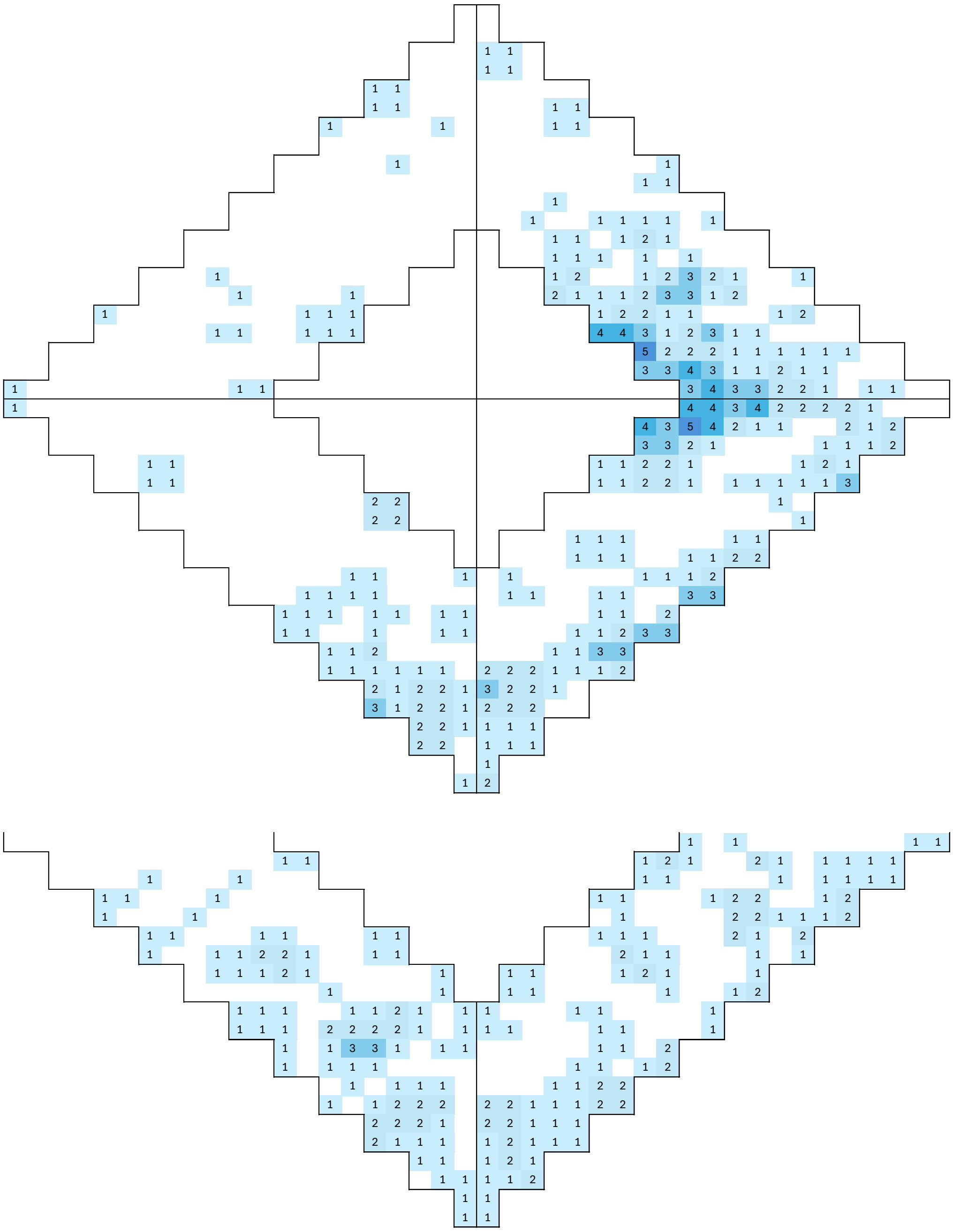

Figure 5. Hit maps of the geometry Doughnut at 25 Hz. Top: Front view; Bottom: Bottom view. The numbers shown are the hit counts within three seconds of milling and footage time. Each number is equally represented by its own color.

Ellipsoid

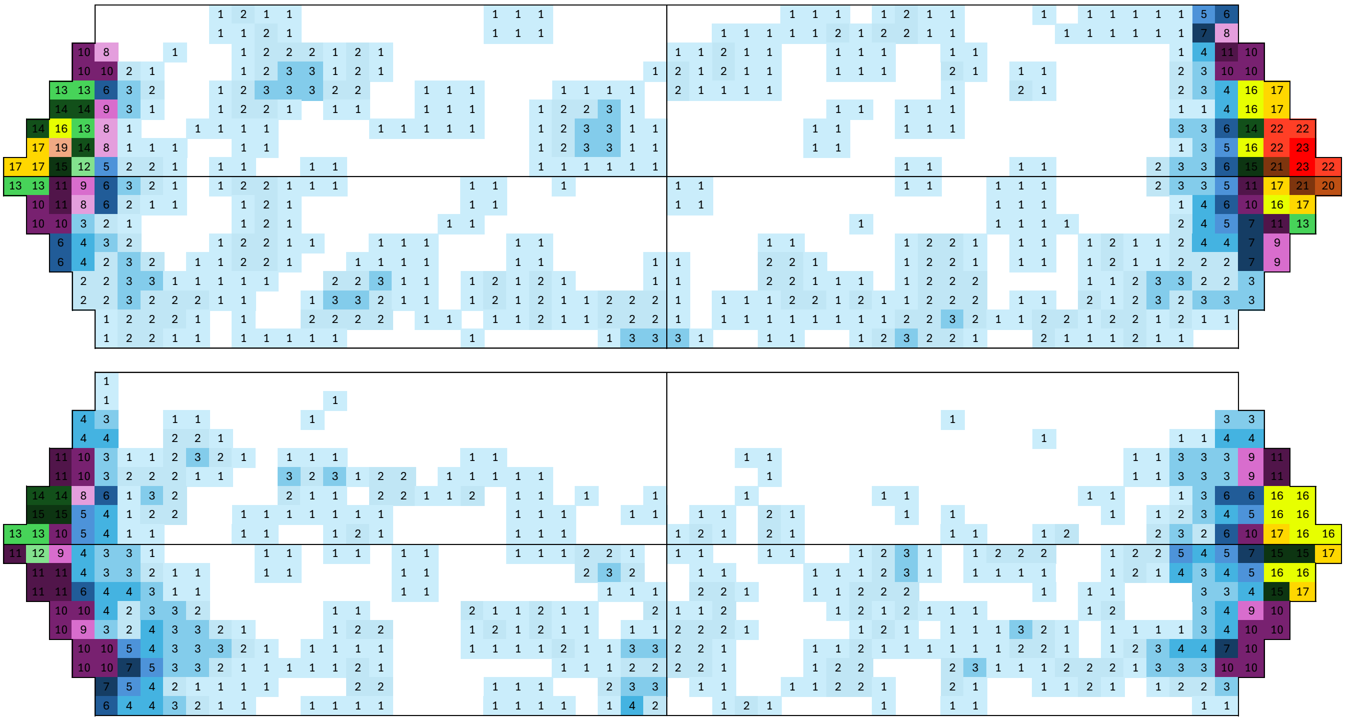

Figure 6. Hit maps of the geometry Ellipsoid at 25 Hz. Top: Front view; Bottom: Bottom view. The numbers shown are the hit counts within three seconds of milling and footage time. Each number is equally represented by its own color.

Ellipsoid big

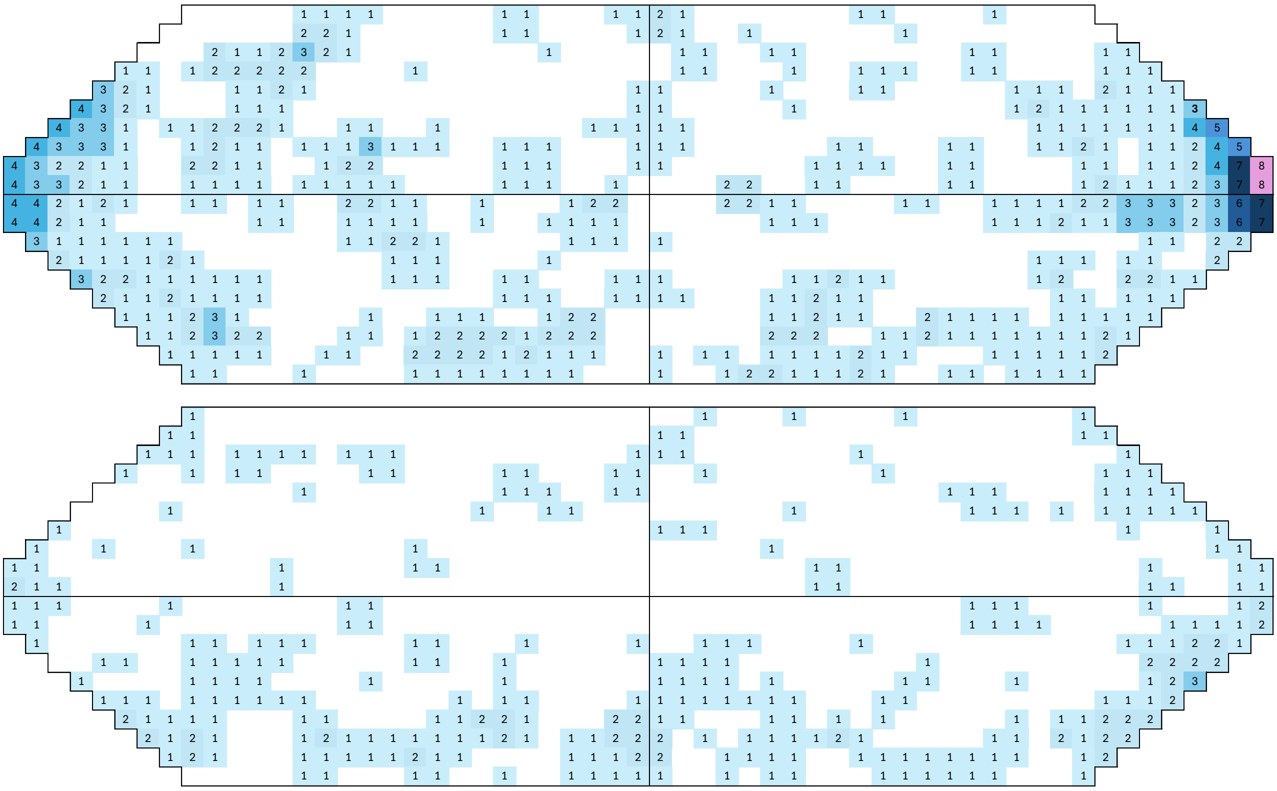

Figure 7. Hit maps of the geometry Ellipsoid big at 25 Hz. Top: Front view; Bottom: Bottom view. The numbers shown are the hit counts within three seconds of milling and footage time. Each number is equally represented by its own color.

Elliptical Orientation 1

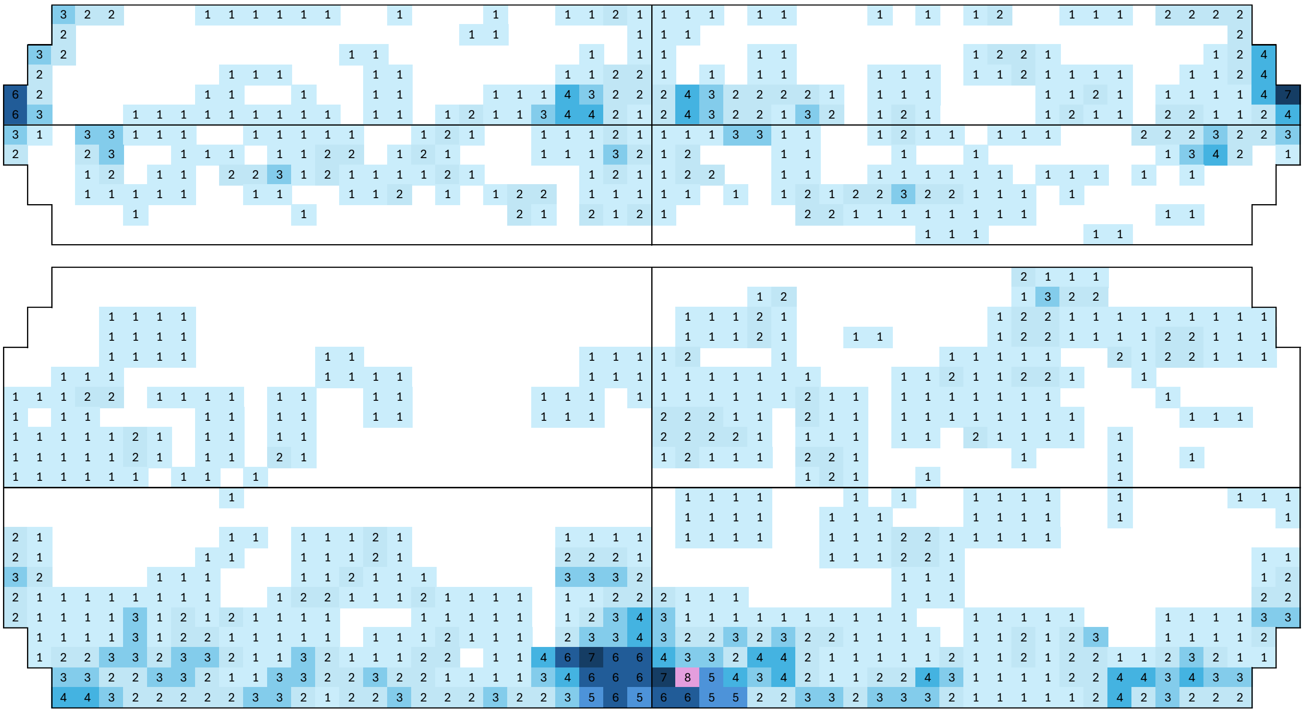

Figure 8. Hit maps of the geometry Elliptical Orientation 1 at 25 Hz. Top: Front view; Bottom: Bottom view. The numbers shown are the hit counts within three seconds of milling and footage time. Each number is equally represented by its own color.

Elliptical Orientation 2

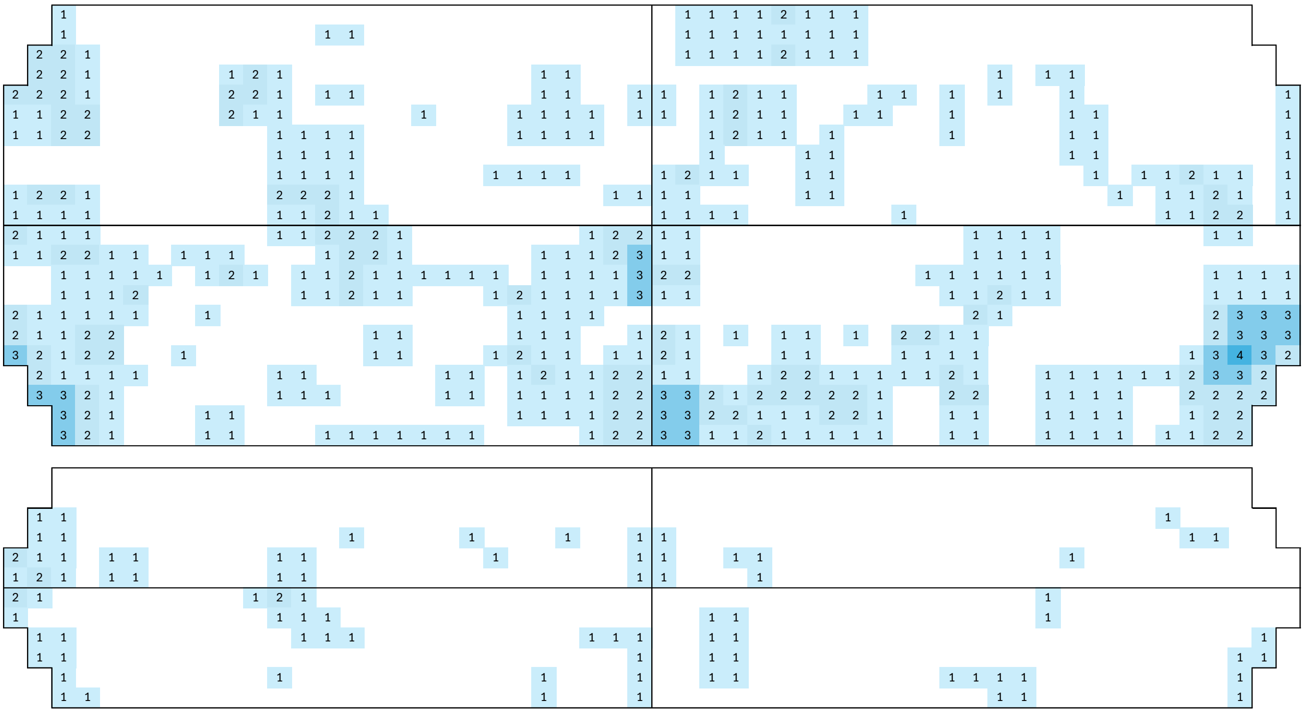

Figure 9. Hit maps of the geometry Elliptical Orientation 2 at 25 Hz. Top: Front view; Bottom: Bottom view. The numbers shown are the hit counts within three seconds of milling and footage time. Each number is equally represented by its own color.

Hexagon

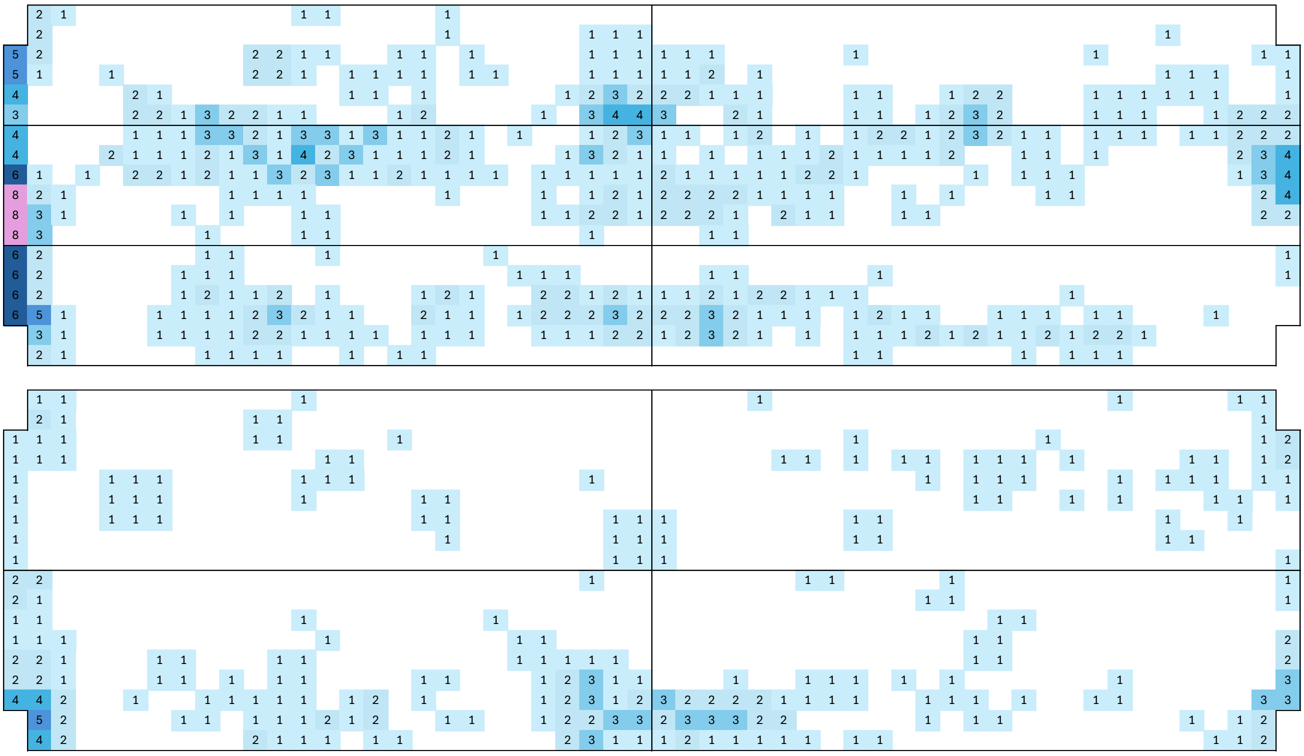

Figure 10. Hit maps of the geometry Hexagon at 25 Hz. Top: Front view; Bottom: Bottom view. The numbers shown are the hit counts within three seconds of milling and footage time. Each number is equally represented by its own color.

Hollow Ring

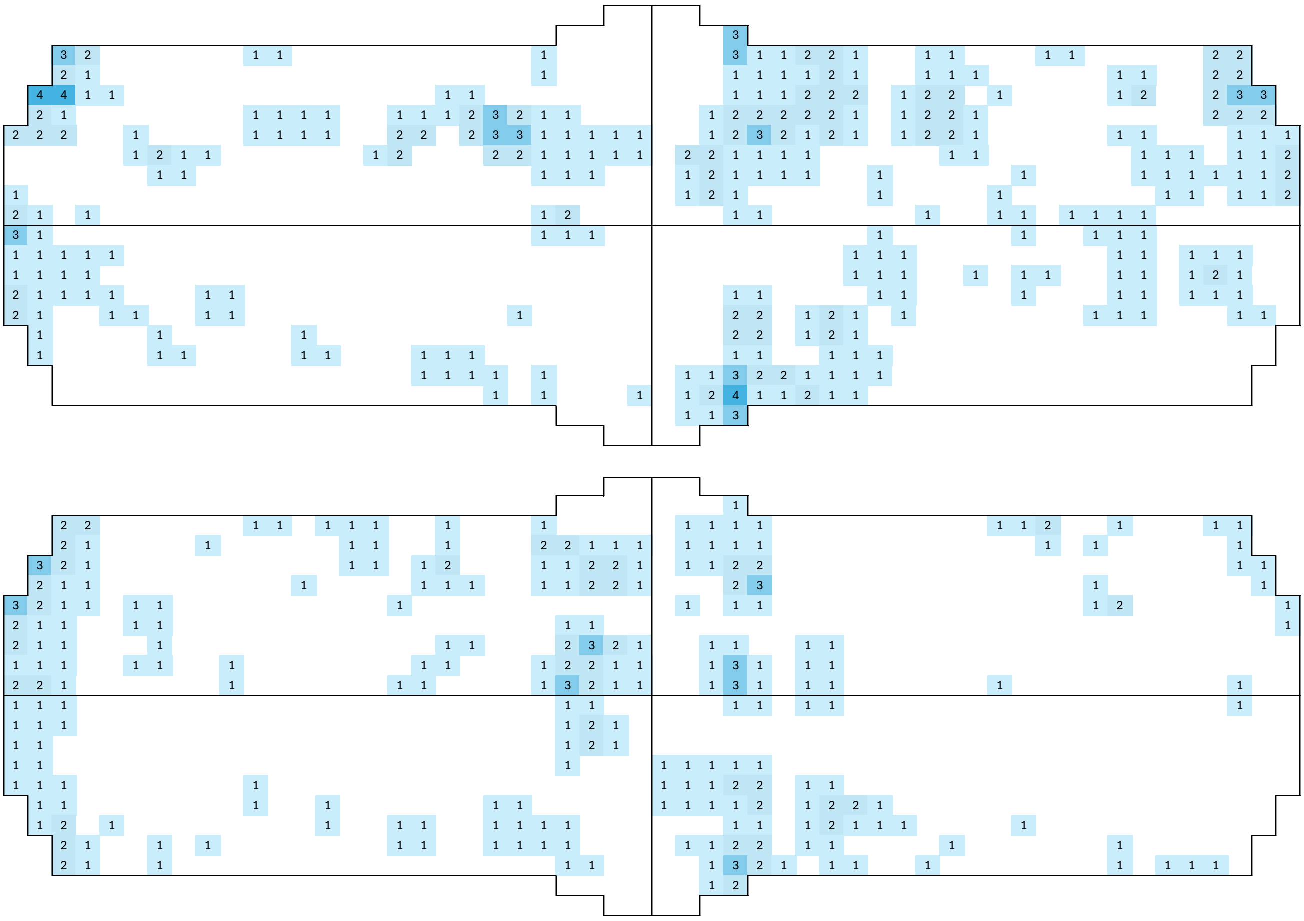

Figure 11. Hit maps of the geometry Hollow Ring at 25 Hz. Top: Front view; Bottom: Bottom view. The numbers shown are the hit counts within three seconds of milling and footage time. Each number is equally represented by its own color.

Hourglass

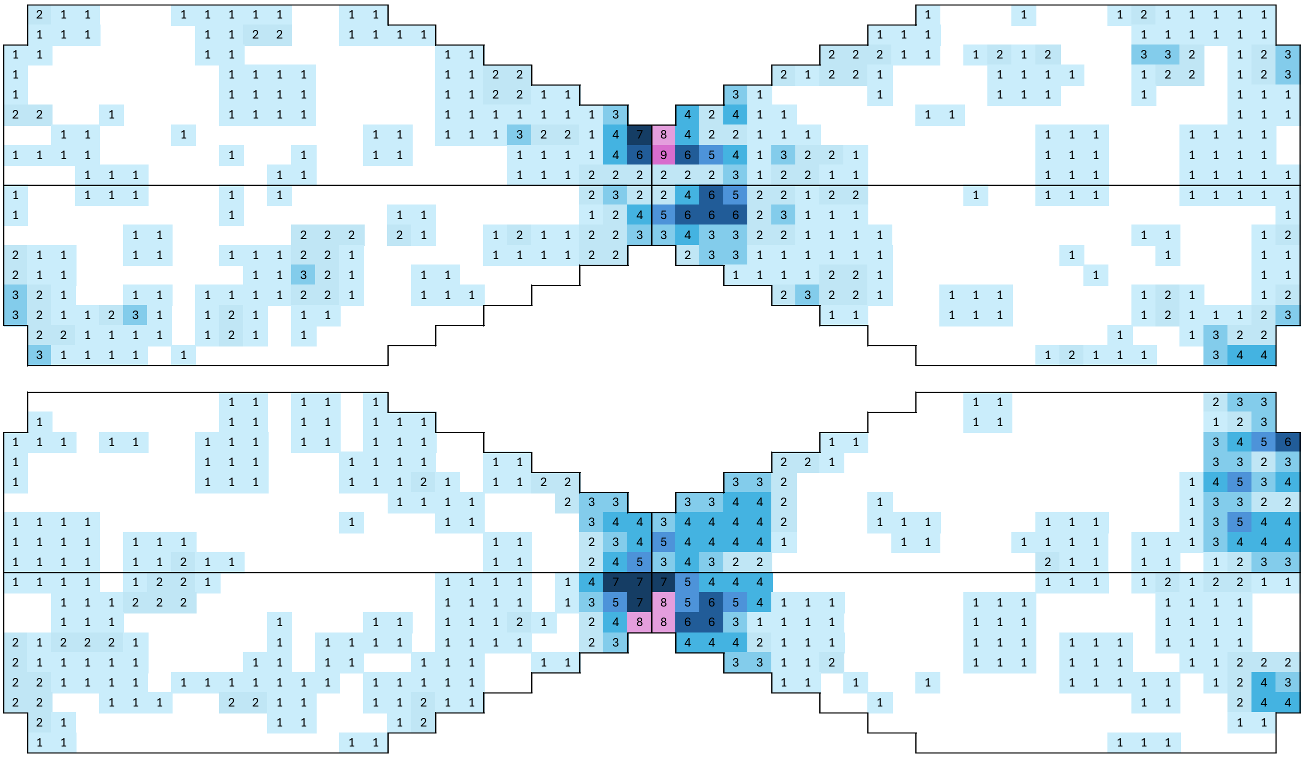

Figure 12. Hit maps of the geometry Hourglass at 25 Hz. Top: Front view; Bottom: Bottom view. The numbers shown are the hit counts within three seconds of milling and footage time. Each number is equally represented by its own color.

Long & Thin

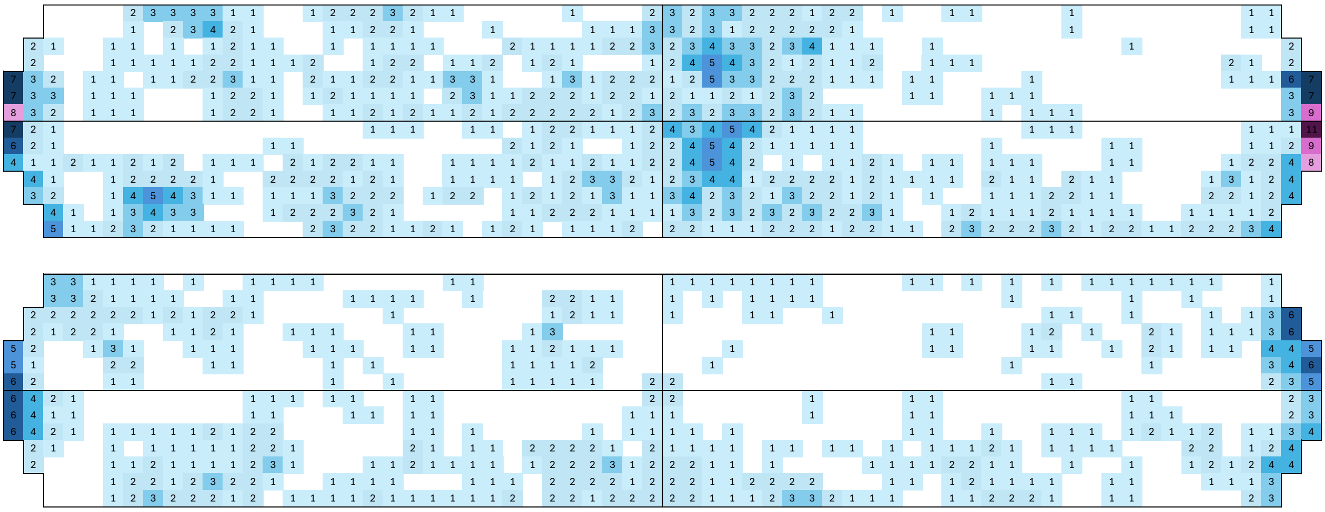

Figure 13. Hit maps of the geometry Long & Thin at 25 Hz. Top: Front view; Bottom: Bottom view. The numbers shown are the hit counts within three seconds of milling and footage time. Each number is equally represented by its own color.

WILEY-VCH

Octahedron

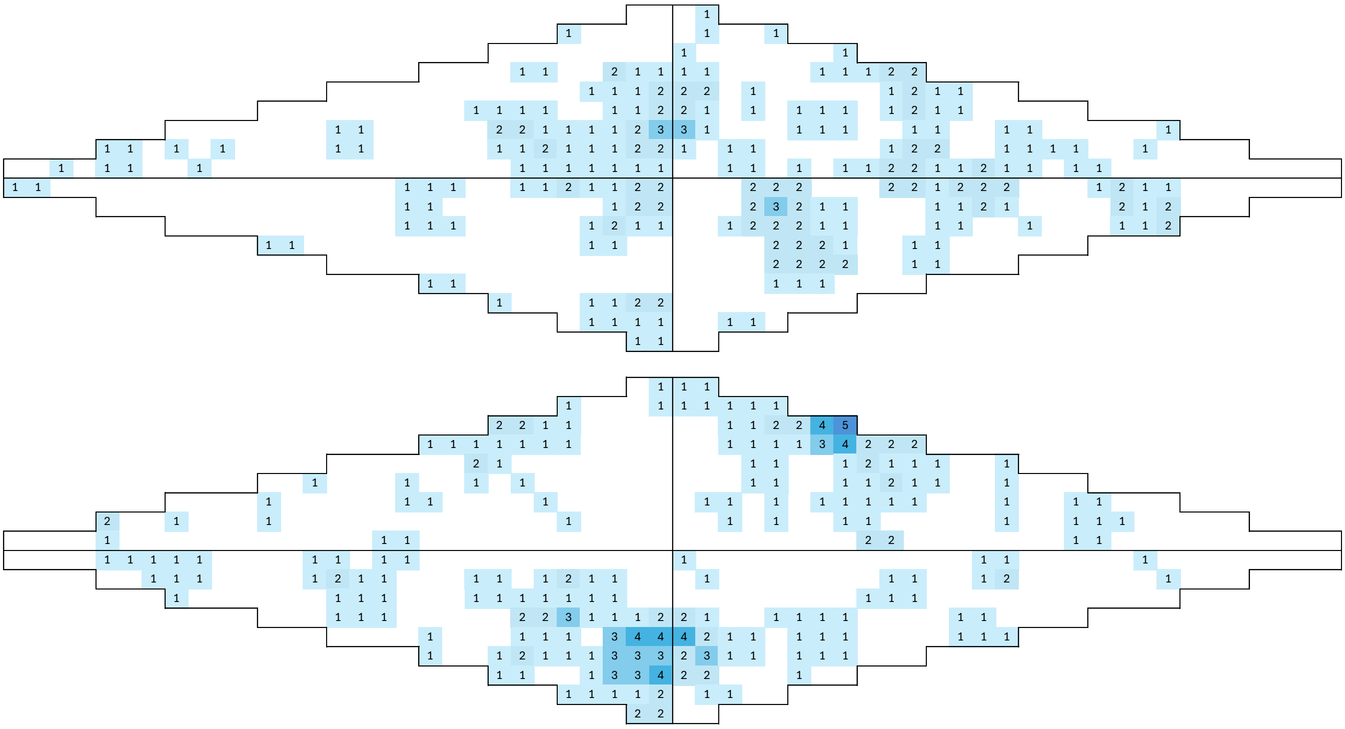

Figure 14. Hit maps of the geometry Octahedron at 25 Hz. Top: Front view; Bottom: Bottom view. The numbers shown are the hit counts within three seconds of milling and footage time. Each number is equally represented by its own color.

Reference

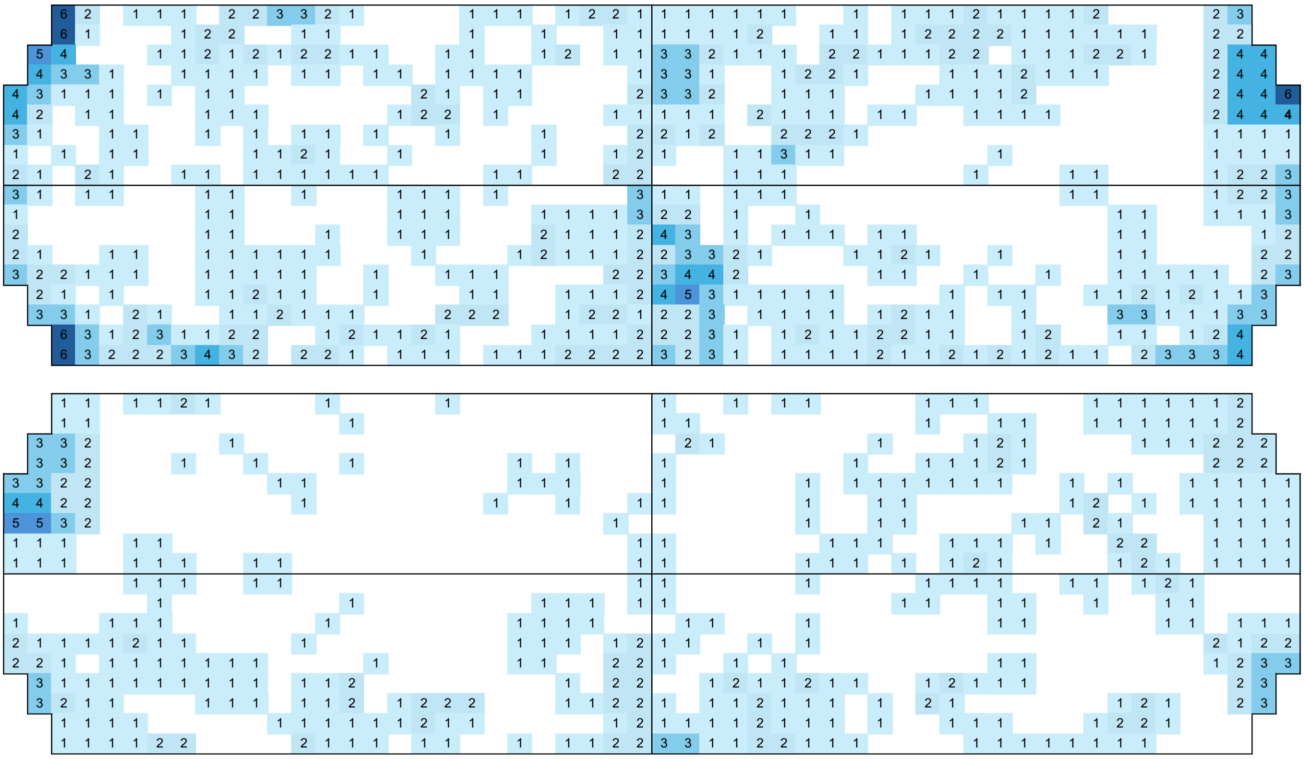

Figure 15. Hit maps of the geometry Reference at 25 Hz. Top: Front view; Bottom: Bottom view. The numbers shown are the hit counts within three seconds of milling and footage time. Each number is equally represented by its own color.

Short & Thick

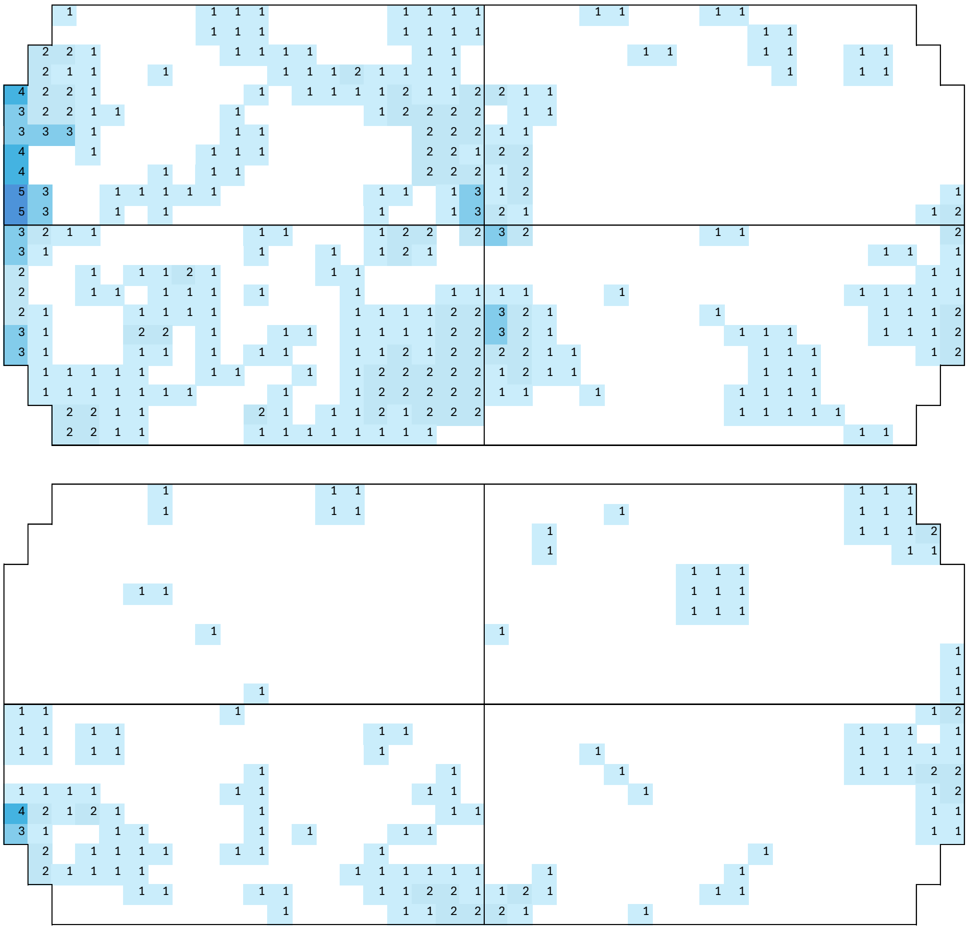

Figure 16. Hit maps of the geometry Short & Thick at 25 Hz. Top: Front view; Bottom: Bottom view. The numbers shown are the hit counts within three seconds of milling and footage time. Each number is equally represented by its own color.

Speedbump

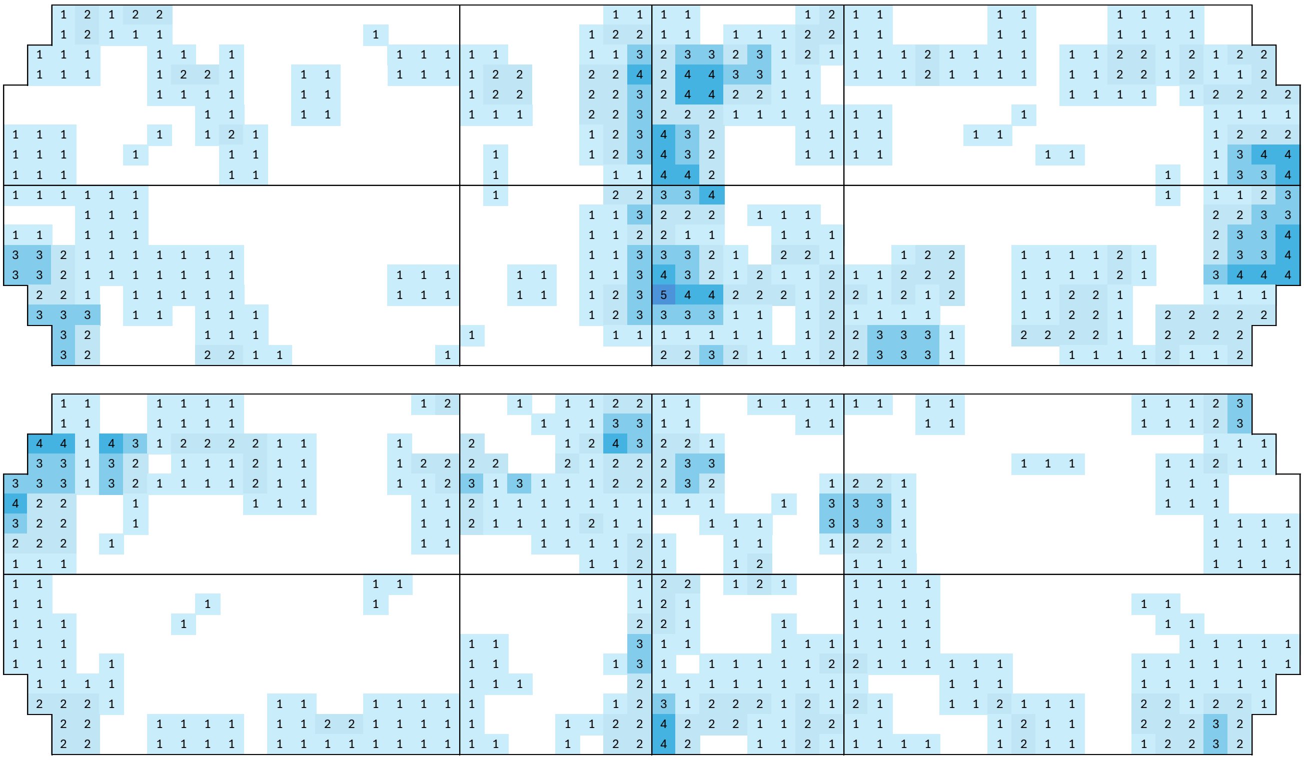

Figure 17. Hit maps of the geometry Speedbump at 25 Hz. Top: Front view; Bottom: Bottom view. The numbers shown are the hit counts within three seconds of milling and footage time. Each number is equally represented by its own color.

Spherical:

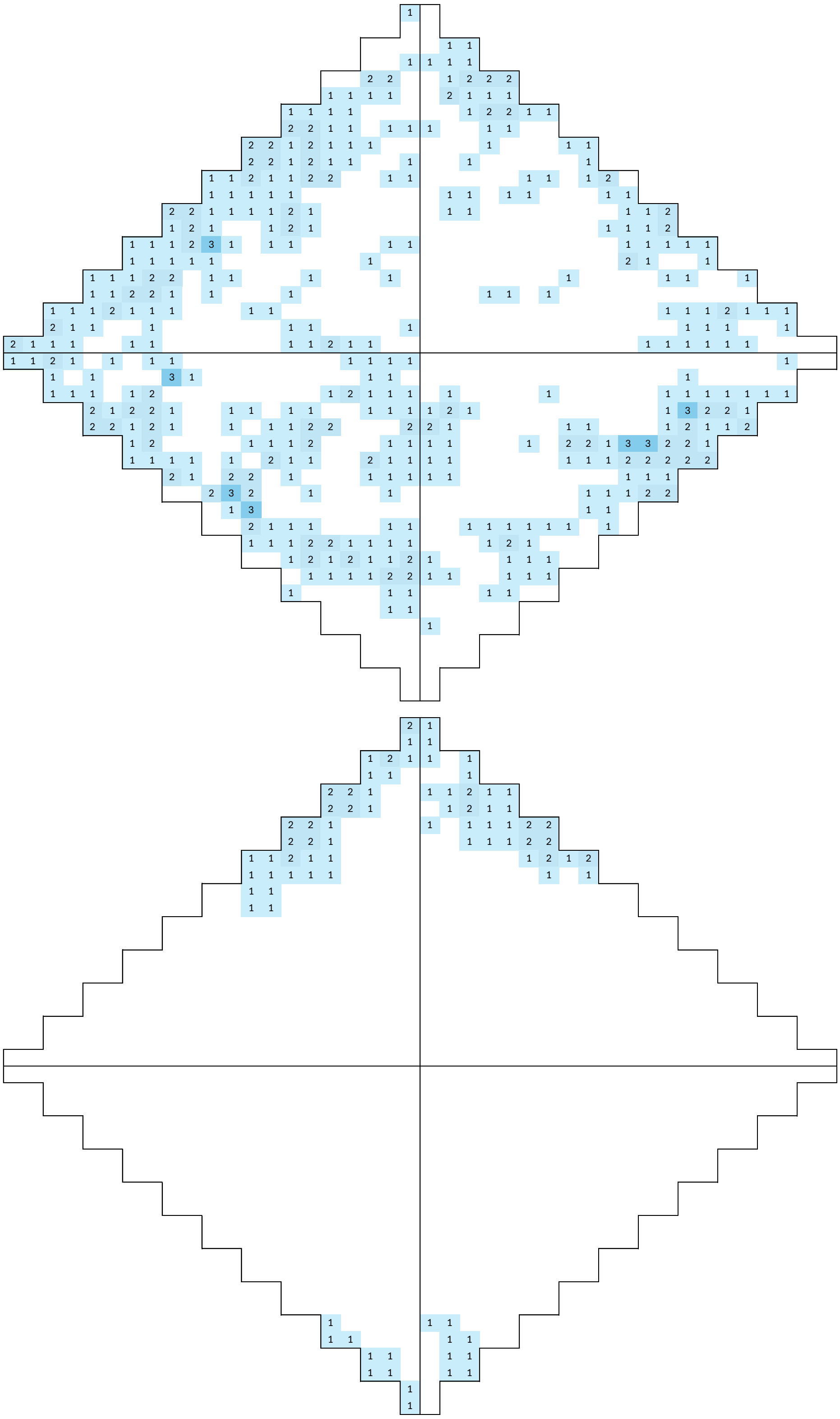

Figure 18. Hit maps of the geometry Spherical at 25 Hz. Top: Front view; Bottom: Bottom view. The numbers shown are the hit counts within three seconds of milling and footage time. Each number is equally represented by its own color.

Triangle

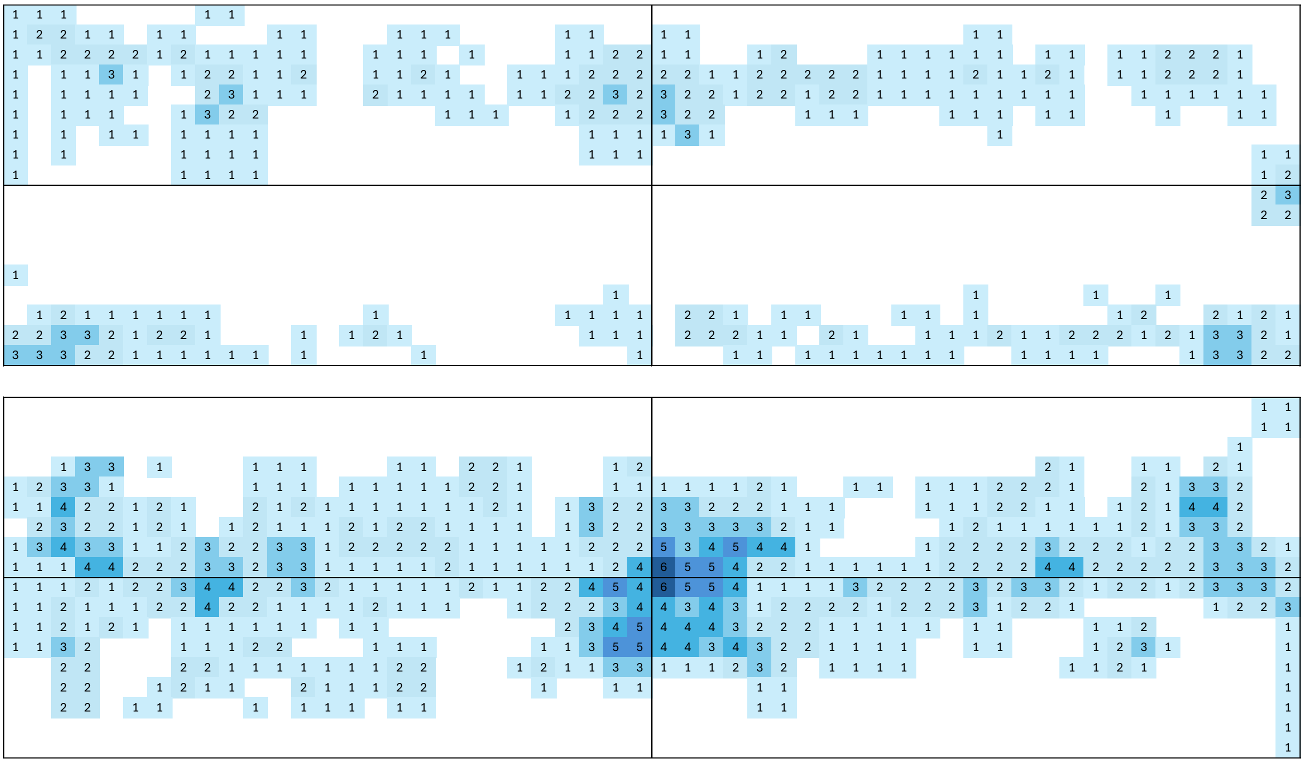

Figure 19. Hit maps of the geometry Triangle at 25 Hz. Top: Front view; Bottom: Bottom view. The numbers shown are the hit counts within three seconds of milling and footage time. Each number is equally represented by its own color.

Turned Hexagon

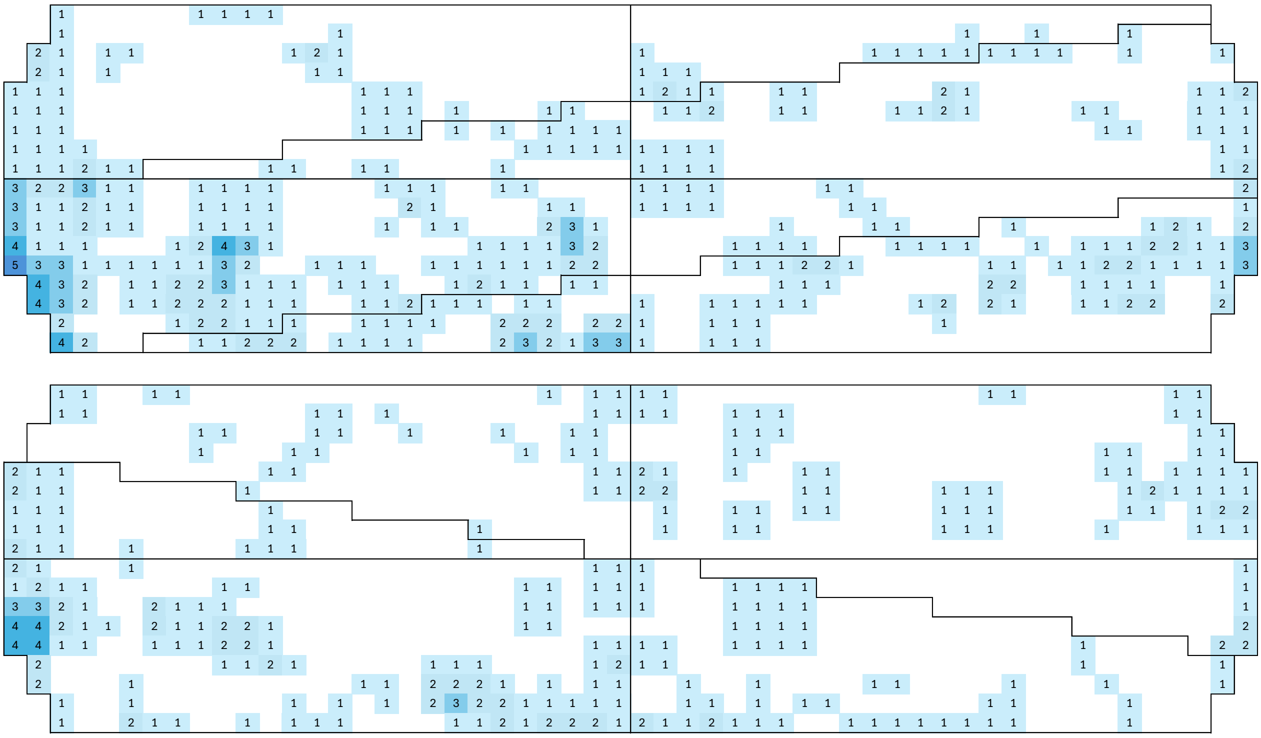

Figure 20. Hit maps of the geometry Turned Hexagon at 25 Hz. Top: Front view; Bottom: Bottom view. The numbers shown are the hit counts within three seconds of milling and footage time. Each number is equally represented by its own color.

Waves:

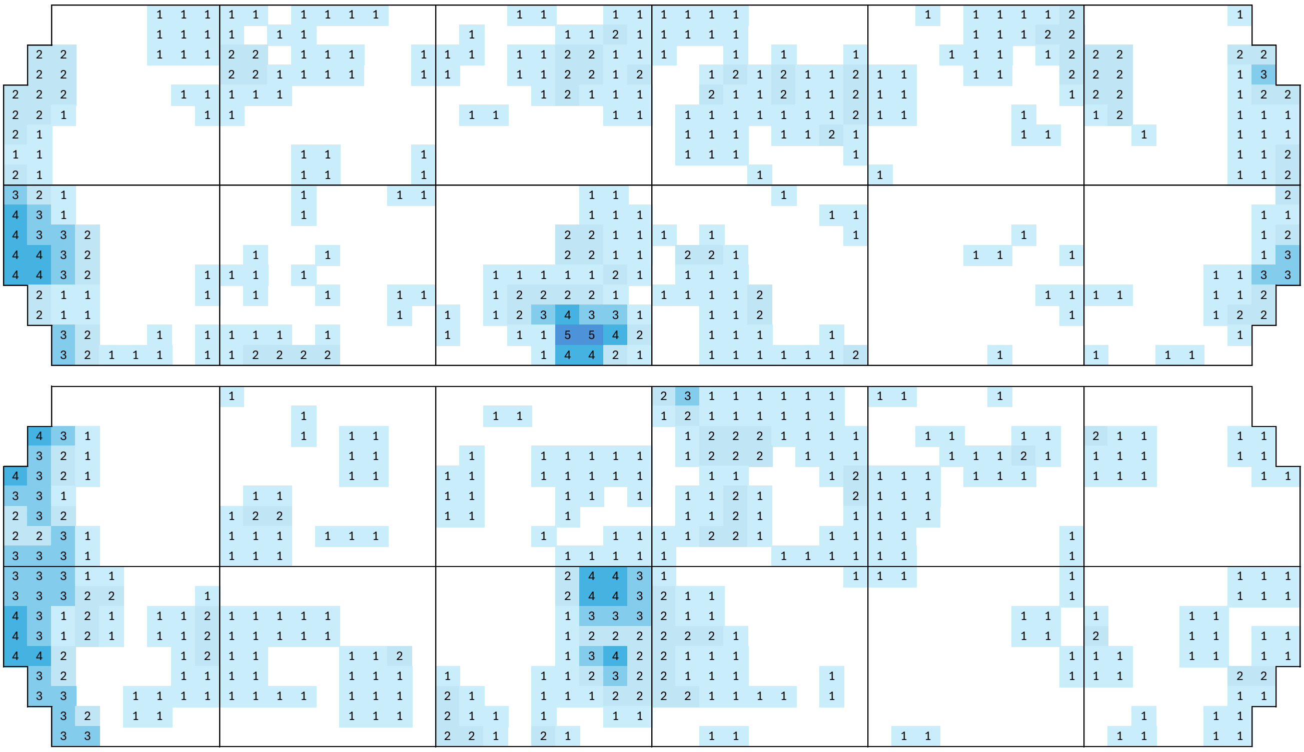

Figure 21. Hit maps of the geometry Waves at 25 Hz. Top: Front view; Bottom: Bottom view. The numbers shown are the hit counts within three seconds of milling and footage time. Each number is equally represented by its own color.

Table 2. Hit counts of the geometries at 25 Hz ordered alphabetically. The reference geometry is marked in light gray.

	Front View	Bottom View	Overall
Banana Orientation 1	1'354	737	2'091
Banana Orientation 2	471	689	1'160
Double Speedbump	978	853	1'831
Double Wave	937	777	1'714
Doughnut	411	228	639
Ellipsoid	1'646	1'449	3'095
Ellipsoid big	741	356	1'097
Elliptical Orientation 1	483	963	1'446
Elliptical Orientation 2	648	88	736
Hexagon	667	323	990
Hollow Ring	410	310	720
Hourglass	653	725	1'378
Long & Thin	1'086	674	1'760
Octahedron	271	275	546
Reference	867	511	1'378
Short & Thick	450	167	617
Speedbump	785	635	1'420
Spherical	495	100	595
Triangle	435	903	1'338
Turned Hexagon	485	353	838
Waves	515	535	1'050

6 Frequency variation and effect on hit maps and hit counts

In the following presentations, the hit maps of the geometries Banana Orientation 1, Double Speedbump, Double Wave, Ellipsoid, Long and Thin, Octahedron, and Reference at milling frequencies of 20 and 30 Hz are depicted. Listed in alphabetical order, they represent the five best, the worst and the reference geometry. Table 3 shows all hit counts of the frequency variation.

Banana Orientation 1, 20 Hz:

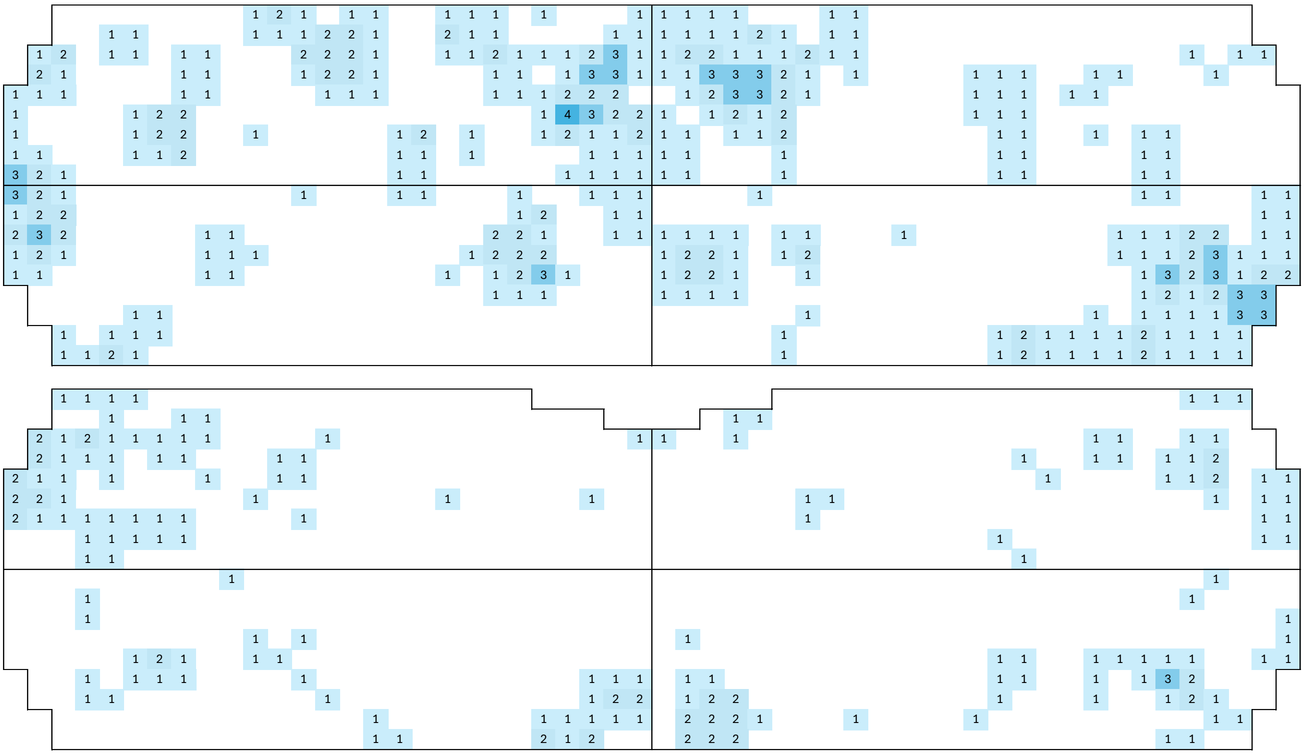

Figure 22. Hit maps of the geometry Banana Orientation 1 at 20 Hz. Top: Front view; Bottom: Bottom view. The numbers shown are the hit counts within three seconds of milling and footage time. Each number is equally represented by its own color.

Banana Orientation 1, 30 Hz

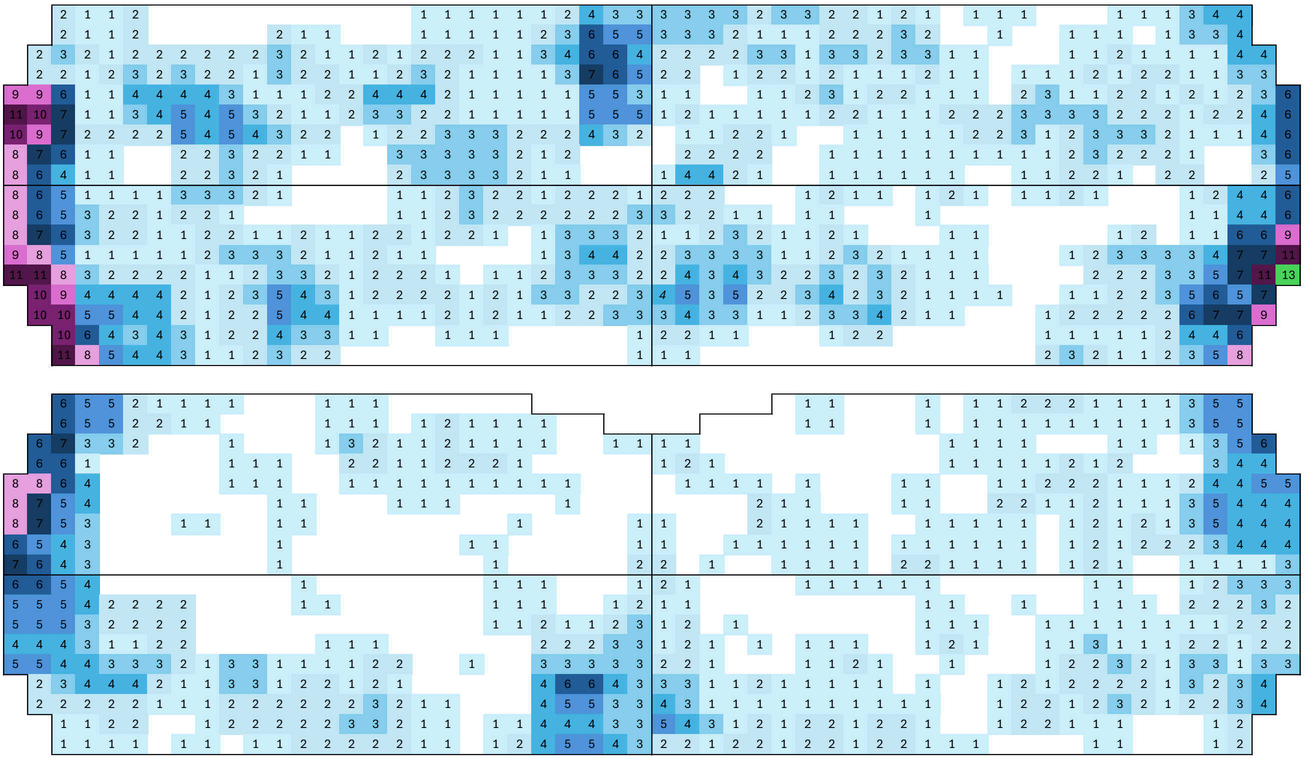

Figure 23. Hit maps of the geometry Banana Orientation 1 at 30 Hz. Top: Front view; Bottom: Bottom view. The numbers shown are the hit counts within three seconds of milling and footage time. Each number is equally represented by its own color.

Double Speedbump 20 Hz

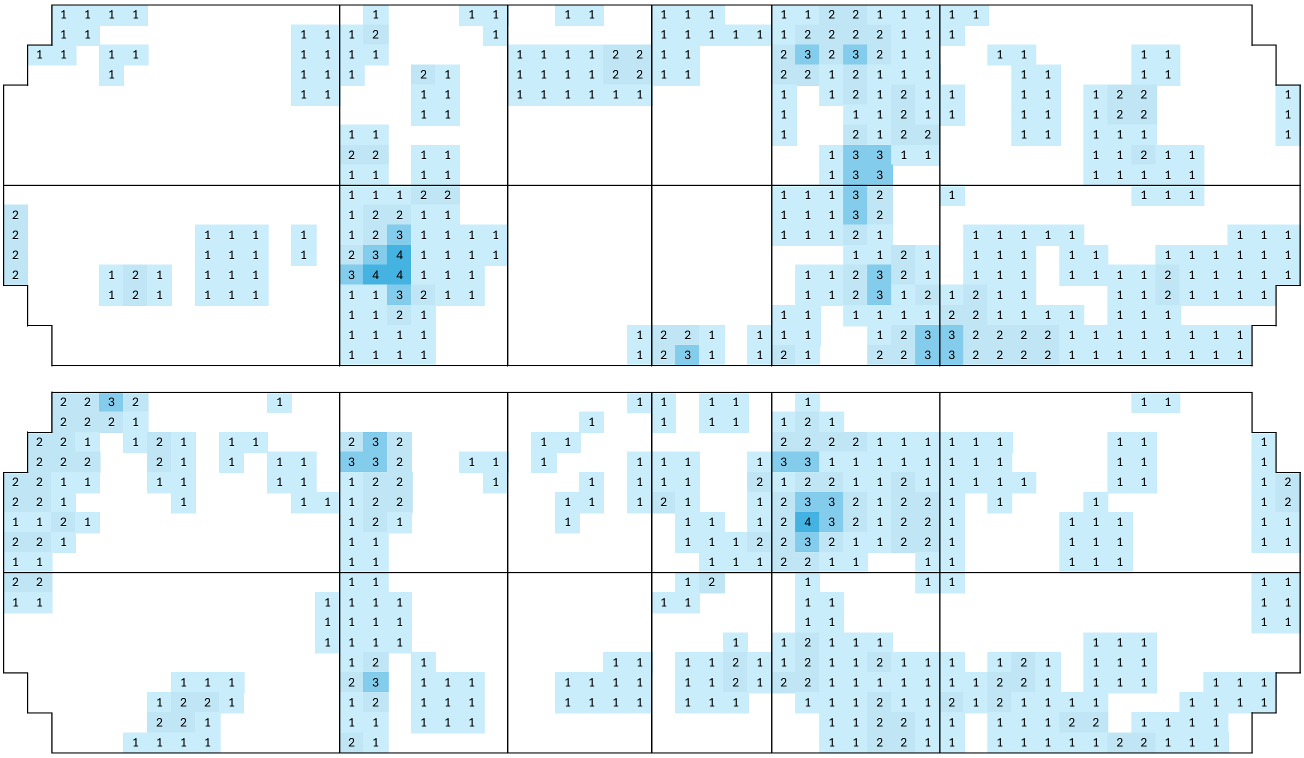

Figure 24. Hit maps of the geometry Double Speedbump at 20 Hz. Top: Front view; Bottom: Bottom view. The numbers shown are the hit counts within three seconds of milling and footage time. Each number is equally represented by its own color.

Double Speedbump 30 Hz:

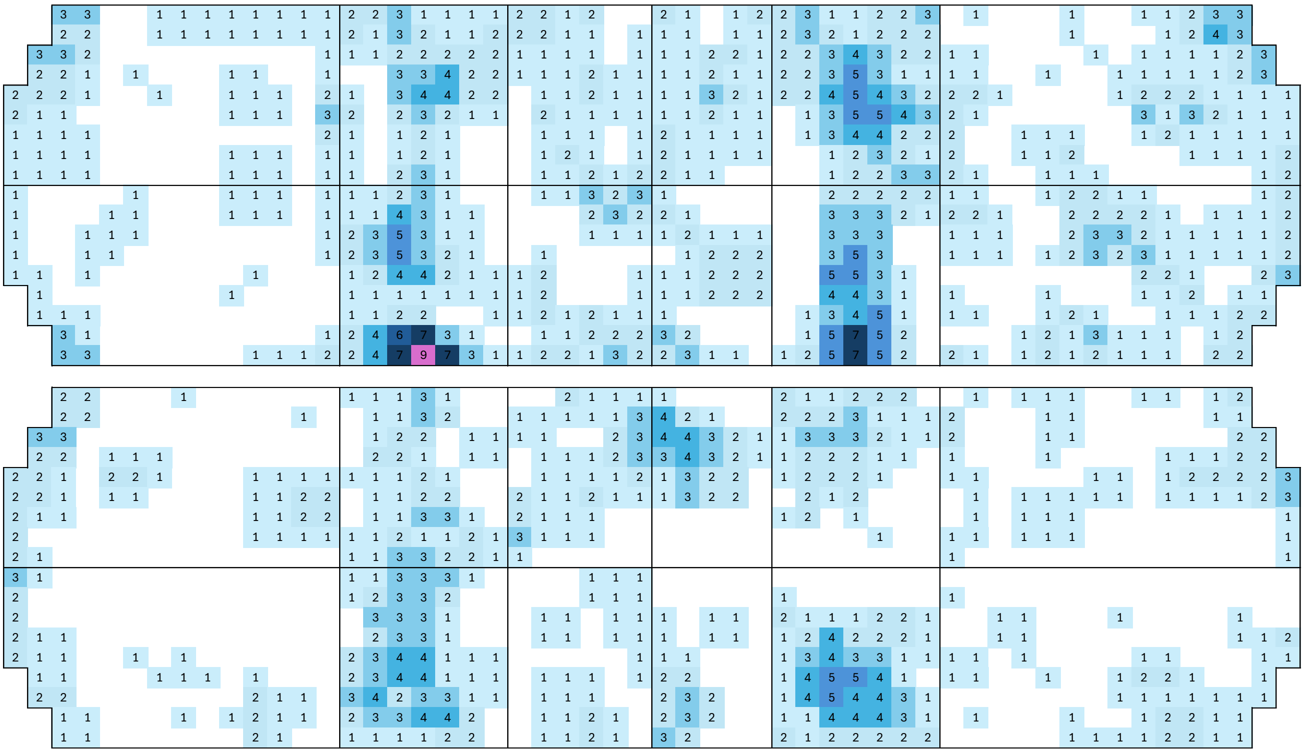

Figure 25. Hit maps of the geometry Double Speedbump at 30 Hz. Top: Front view; Bottom: Bottom view. The numbers shown are the hit counts within three seconds of milling and footage time. Each number is equally represented by its own color.

Double Wave 20 Hz:

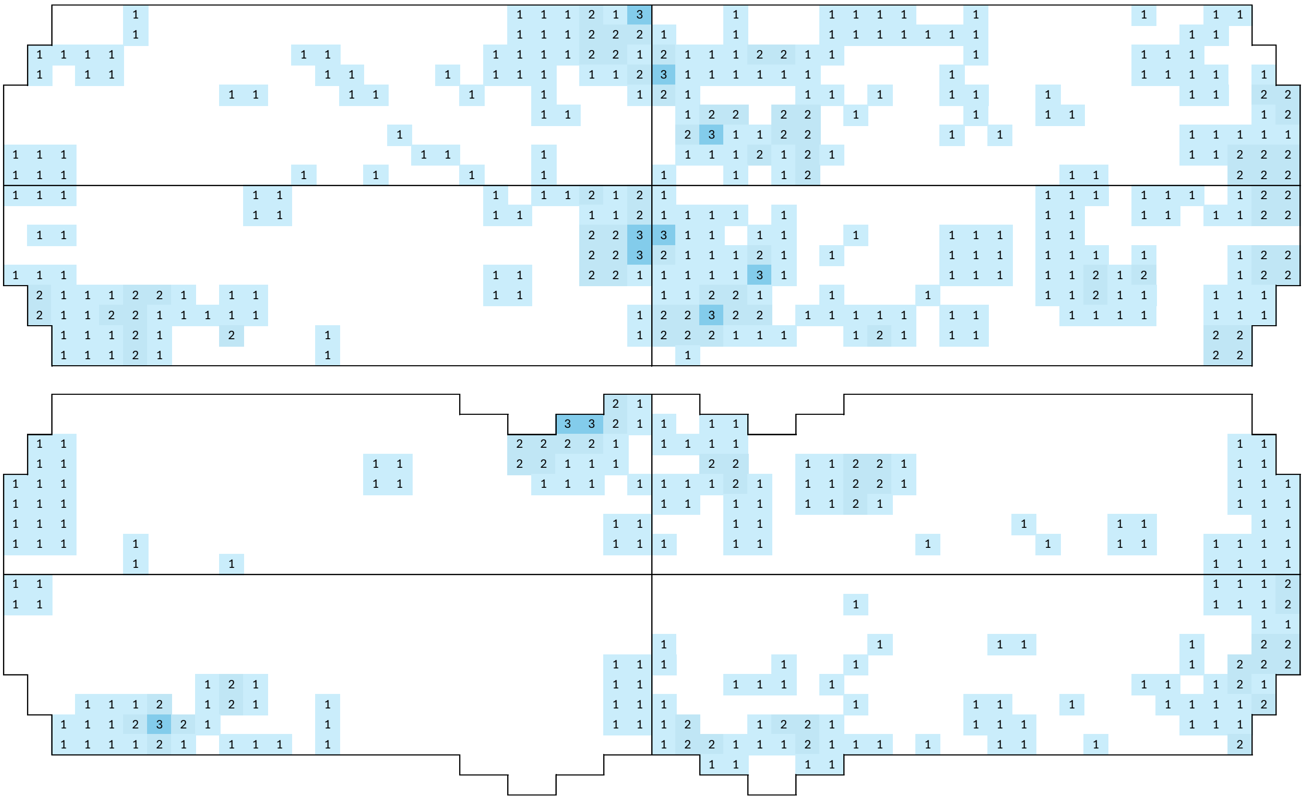

Figure 26. Hit maps of the geometry Double Wave at 20 Hz. Top: Front view; Bottom: Bottom view. The numbers shown are the hit counts within three seconds of milling and footage time. Each number is equally represented by its own color.

Double Wave 30 Hz

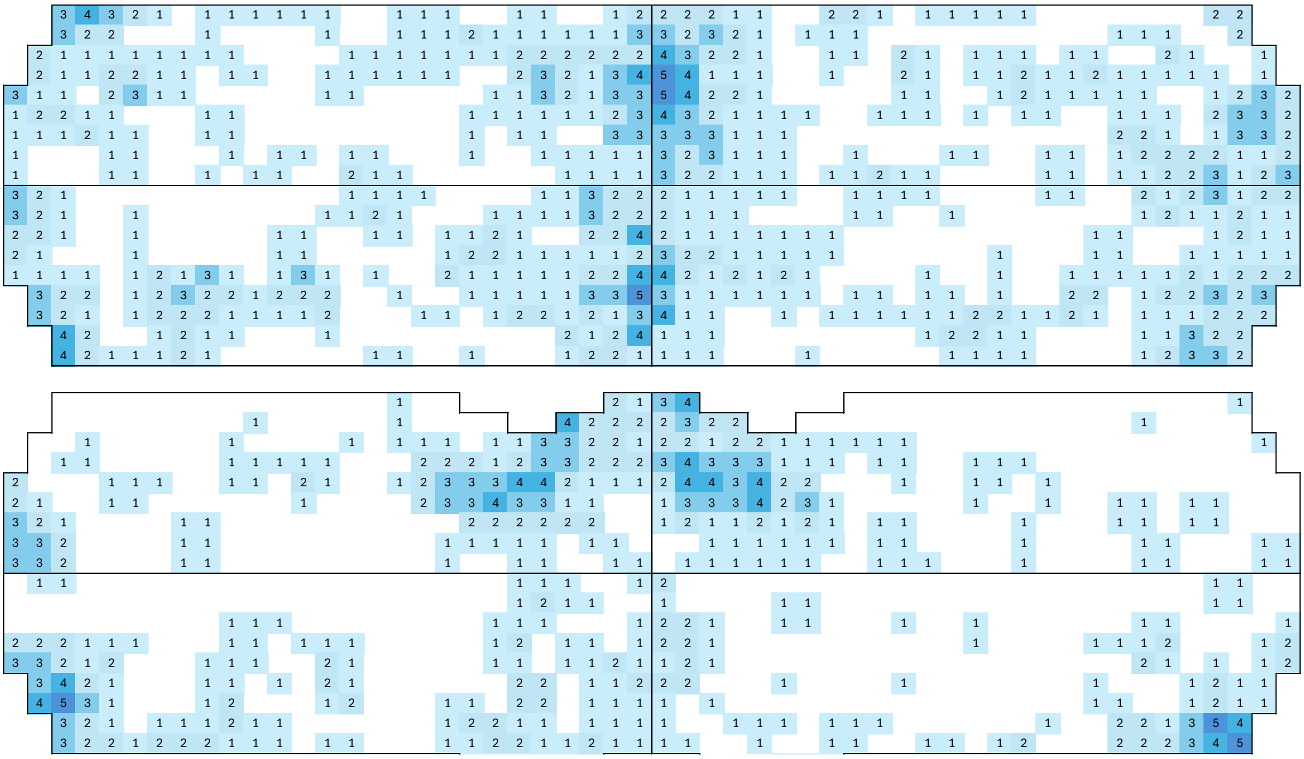

Figure 27. Hit maps of the geometry Double Wave at 30 Hz. Top: Front view; Bottom: Bottom view. The numbers shown are the hit counts within three seconds of milling and footage time. Each number is equally represented by its own color.

Ellipsoid 20 Hz:

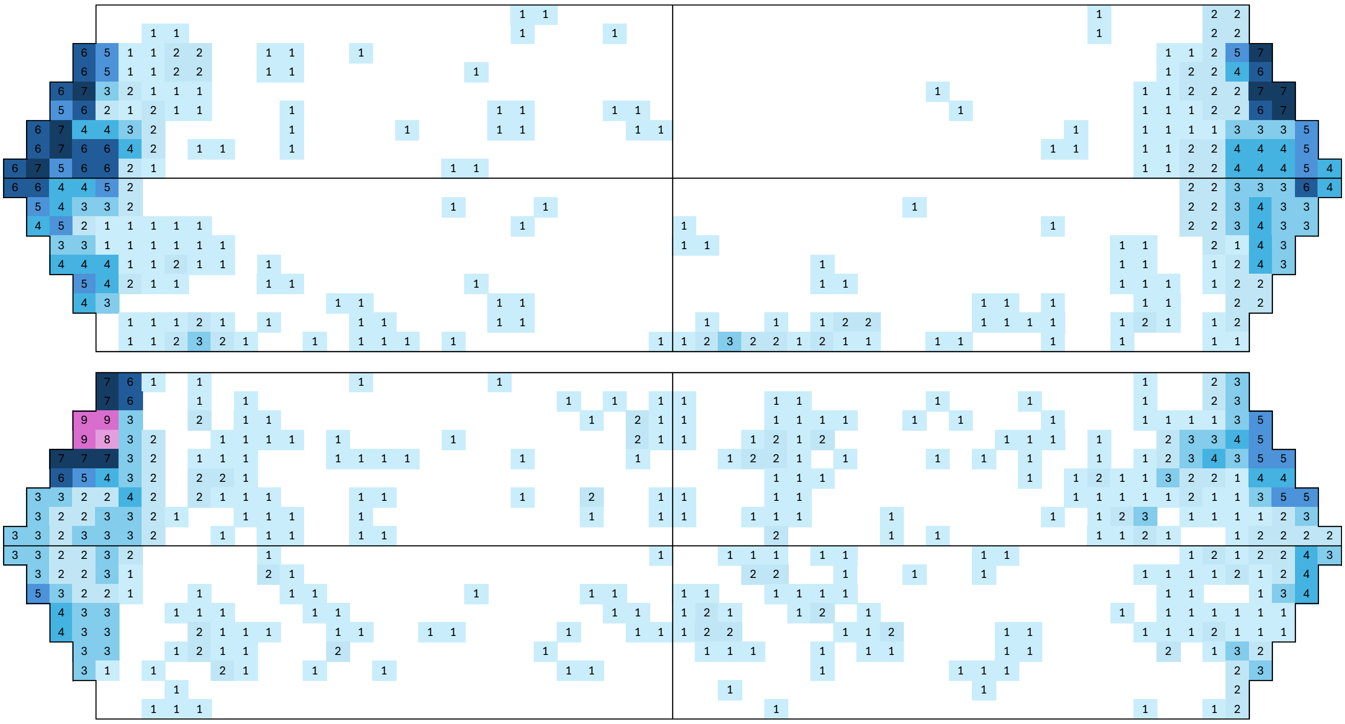

Figure 28. Hit maps of the geometry Ellipsoid at 20 Hz. Top: Front view; Bottom: Bottom view. The numbers shown are the hit counts within three seconds of milling and footage time. Each number is equally represented by its own color.

Ellipsoid 30 Hz:

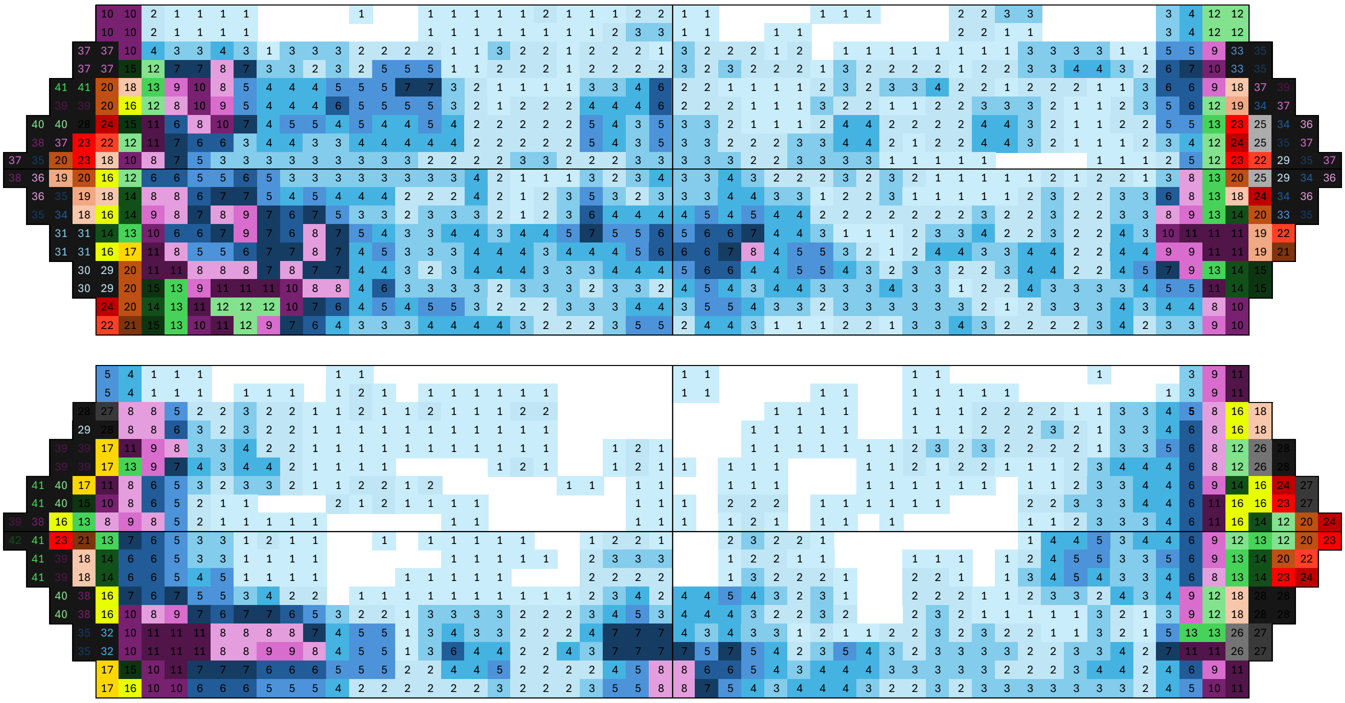

Figure 29. Hit maps of the geometry Ellipsoid at 30 Hz. Top: Front view; Bottom: Bottom view. The numbers shown are the hit counts within three seconds of milling and footage time. Each number is equally represented by its own color.

Long and Thin 20 Hz:

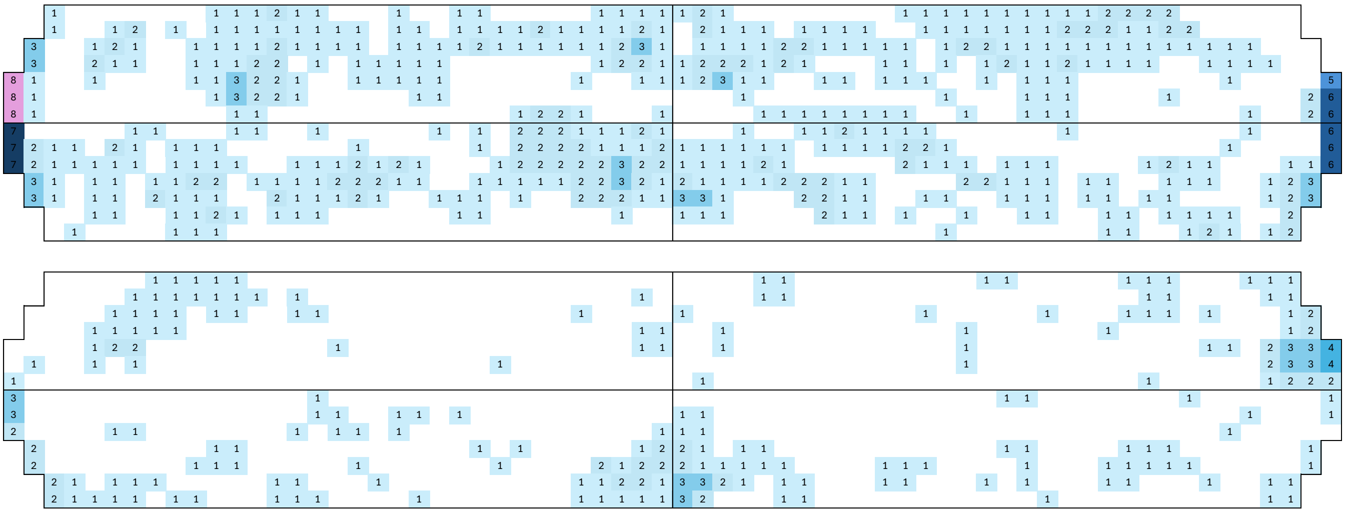

Figure 30. Hit maps of the geometry Long and Thin at 20 Hz. Top: Front view; Bottom: Bottom view. The numbers shown are the hit counts within three seconds of milling and footage time. Each number is equally represented by its own color.

Long and Thin 30 Hz:

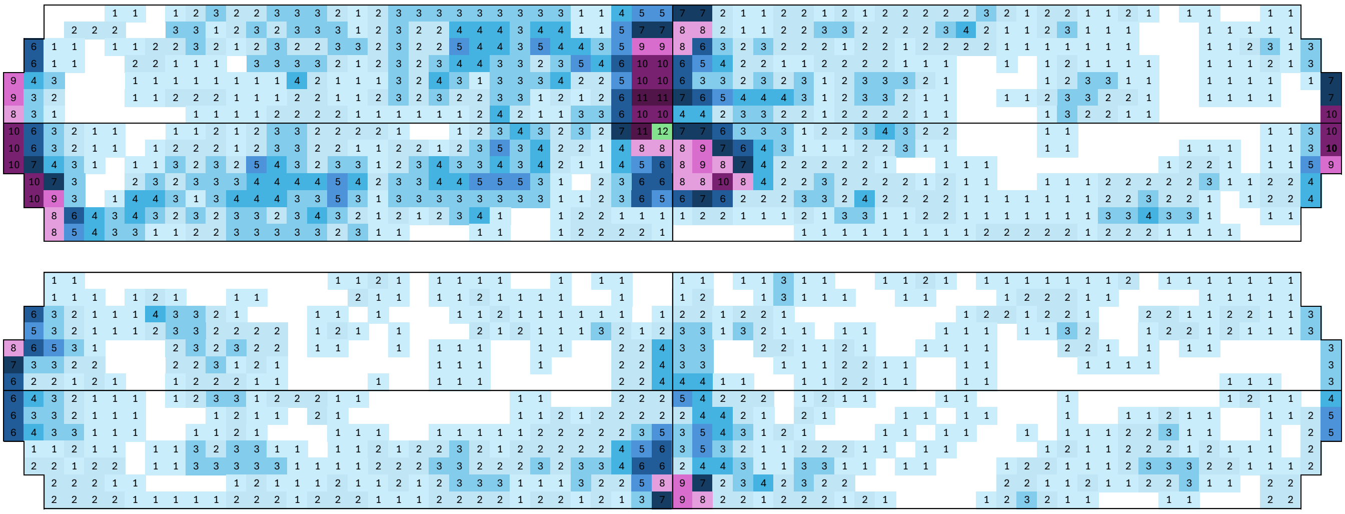

Figure 31. Hit maps of the geometry Long and Thin at 30 Hz. Top: Front view; Bottom: Bottom view. The numbers shown are the hit counts within three seconds of milling and footage time. Each number is equally represented by its own color.

Octahedron 20 Hz:

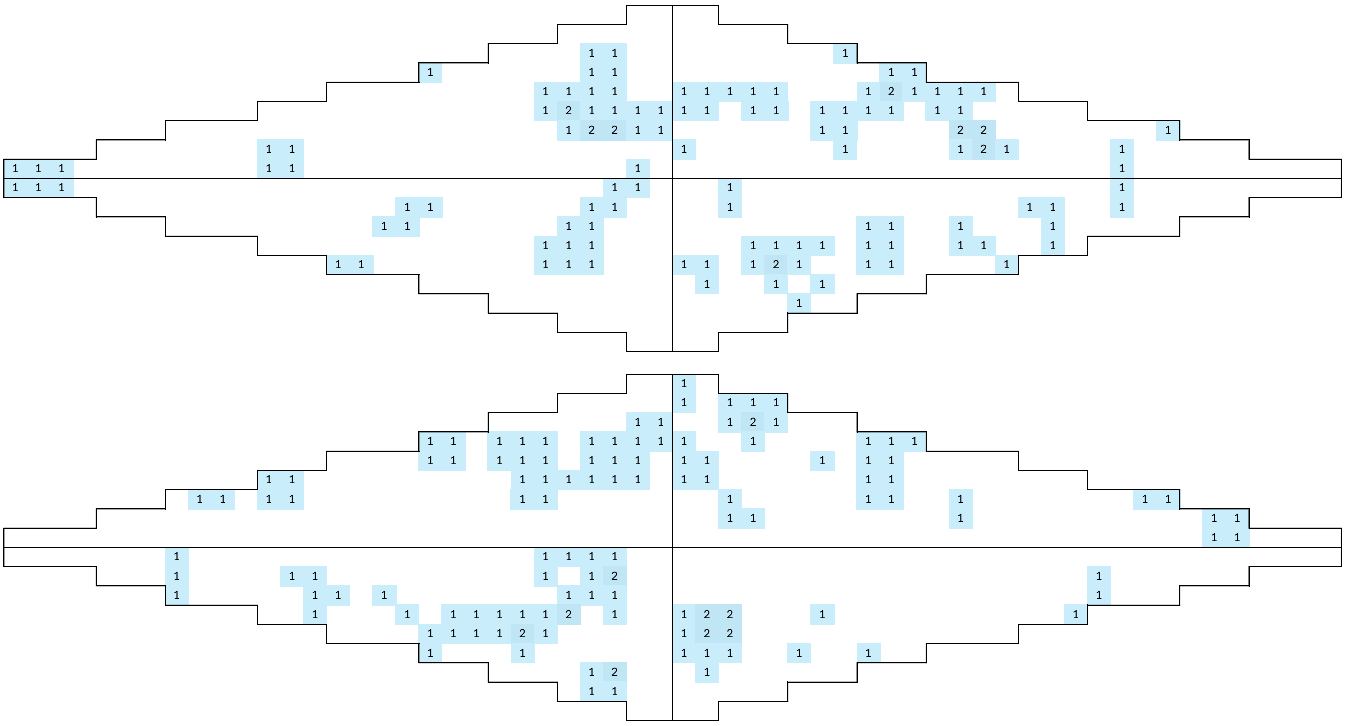

Figure 32. Hit maps of the geometry Octahedron at 20 Hz. Top: Front view; Bottom: Bottom view. The numbers shown are the hit counts within three seconds of milling and footage time. Each number is equally represented by its own color.

Octahedron 30Hz

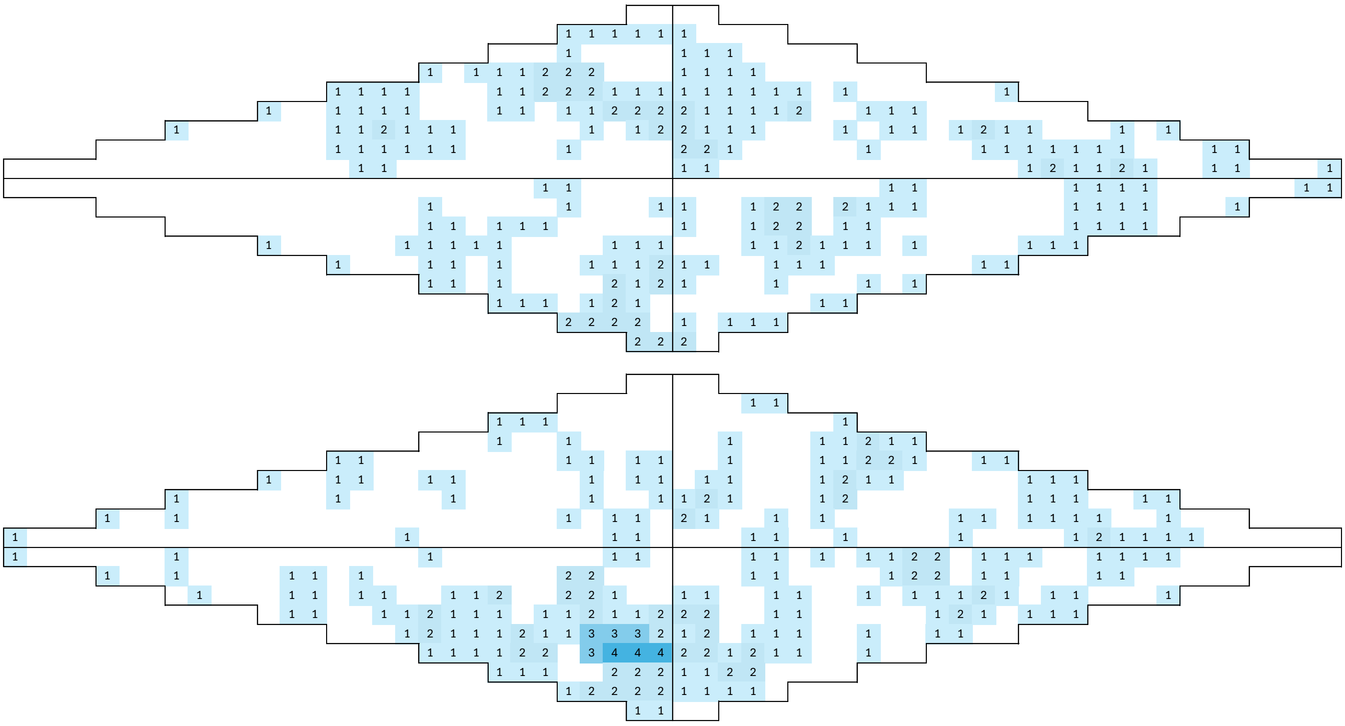

Figure 33. Hit maps of the geometry Octahedron at 30 Hz. Top: Front view; Bottom: Bottom view. The numbers shown are the hit counts within three seconds of milling and footage time. Each number is equally represented by its own color.

Reference 20 Hz:

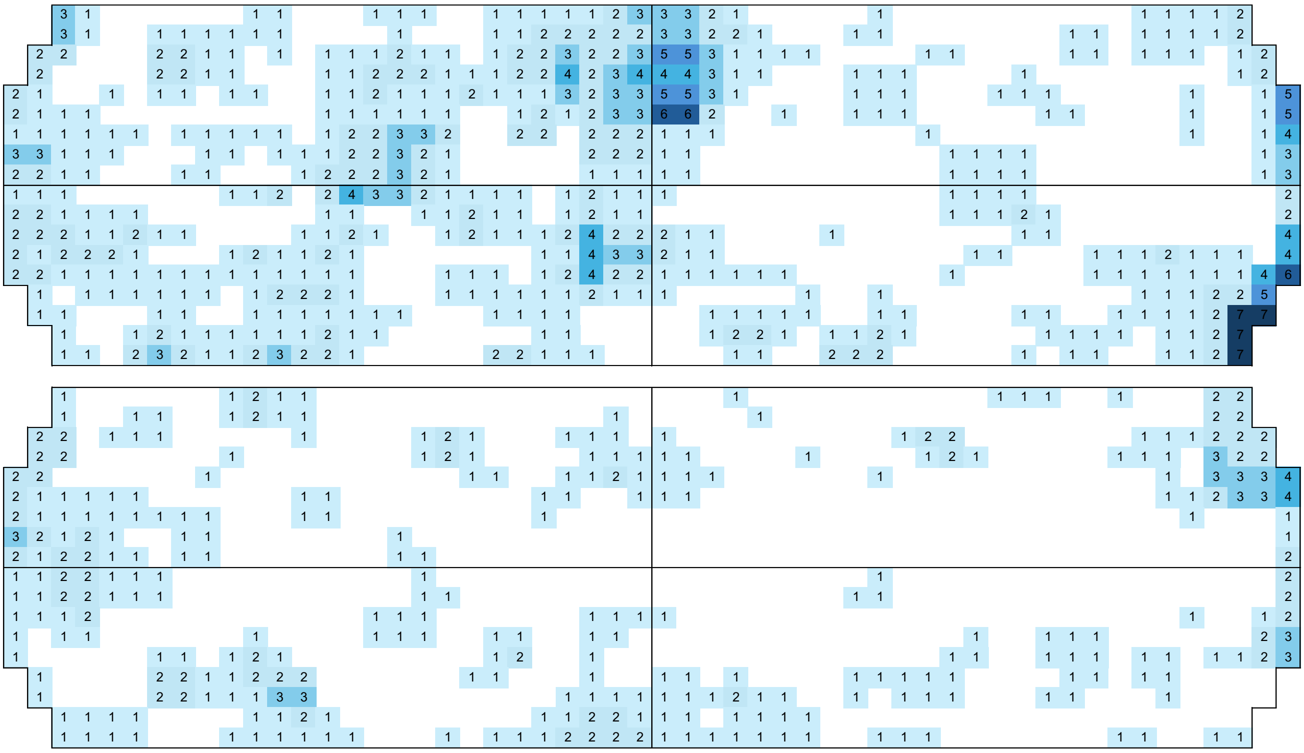

Figure 34. Hit maps of the geometry Reference at 20 Hz. Top: Front view; Bottom: Bottom view. The numbers shown are the hit counts within three seconds of milling and footage time. Each number is equally represented by its own color.

Reference 30 Hz:

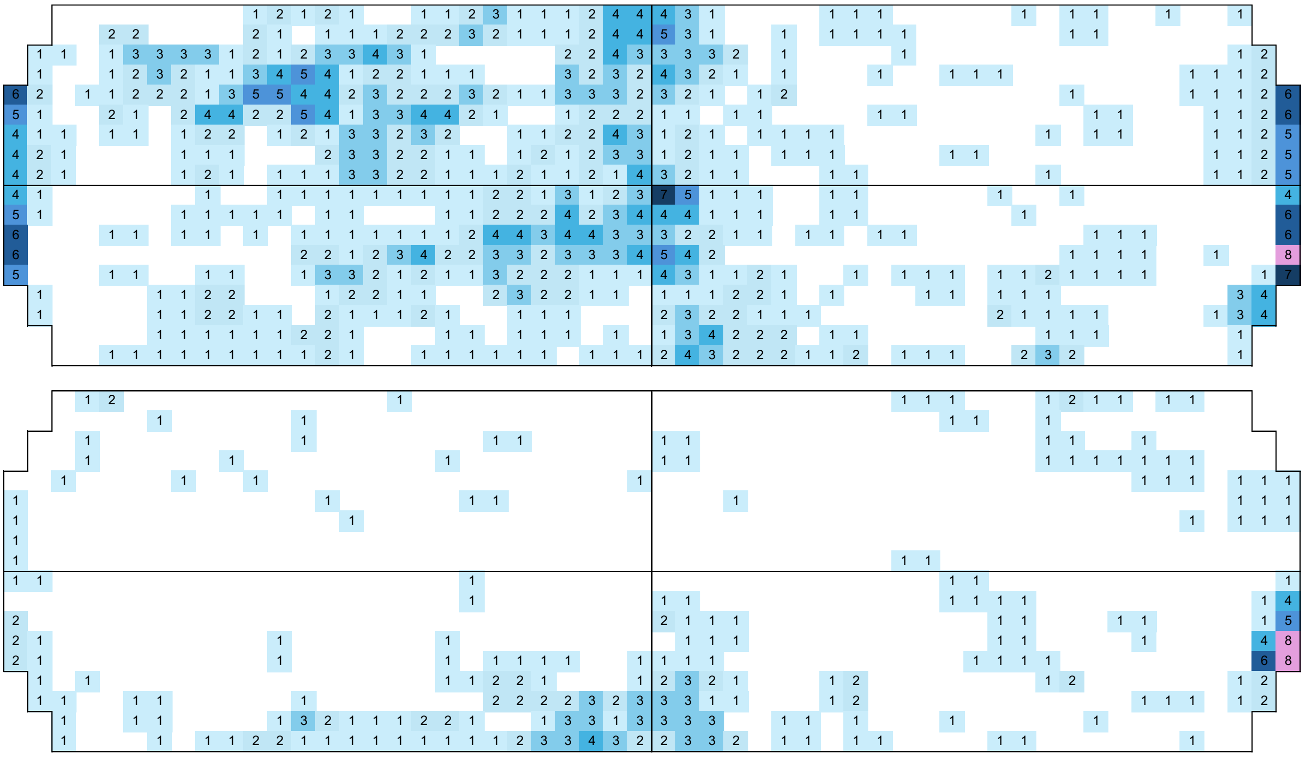

Figure 35. Hit maps of the geometry Reference at 30 Hz. Top: Front view; Bottom: Bottom view. The numbers shown are the hit counts within three seconds of milling and footage time. Each number is equally represented by its own color.

Table 3. Hit counts of the geometries at frequencies of 20 Hz and 30 Hz. The reference geometry is marked in grey.

	20 Hz			30 Hz		
	Front View	Bottom View	Overall	Front View	Bottom View	Overall
Banana Orientation 1	454	193	647	1'961	1'217	3'178
Double Speedbump	500	492	992	1'084	777	1'861
Double Wave	443	271	714	895	630	1'525
Ellipsoid	640	661	1'301	5'989	4'540	10'529
Long and Thin	684	259	943	2'091	1'160	3'251
Octahedron	124	132	256	258	290	548
Reference	810	396	1'206	1'047	315	1'362

7 Time measurements of the light decay

The measurements of the light decay were conducted on a Retsch MM400 mixer mill at a frequency of 25 Hz. To get the right conditions, the mill was placed inside a completely darkened laboratory so that no external light could disturb the time measurement by over-glowing the flashes of the triboluminescent crystals. The time was measured with the stopwatch function of a Samsung Galaxy S20FE. For the measurements, every geometry was loaded with 50 mg of the triboluminescent crystals, and the time was measured between the starting of the milling and the compete decay of the flashes. These experiments were conducted twice, and the times were averaged. The results are shown in Table 4.

Table 4. Time measurements until complete light decay of 50 mg of triboluminescent crystals.

Geometrie	time 1 [s]	time 2 [s]	Average [s]
Banana Orientation 1	34.73	36.62	35.68
Banana Orientation 2	47.63	65.72	56.68
Double Speedbump	47.73	52.40	50.07
Double Wave	43.71	49.08	46.40
Doughnut	76.65	103.20	89.93
Ellipsoid	34.74	35.80	35.27
Ellipsoid big	49.41	42.01	45.71
Elliptical Orientation 1	49.51	77.36	63.44
Elliptical Orientation 2	74.89	100.71	87.80
Hexagon	56.31	54.00	55.16
Hollow Ring	43.75	79.97	61.86
Hourglass	42.64	67.69	55.17
Long & Thin	39.99	48.60	44.30
Octahedron	86.08	110.41	98.25
Reference	57.71	67.94	62.83
Short & Thick	54.93	55.92	55.43
Speedbump	41.30	55.32	48.31
Spherical	65.83	86.93	76.38
Triangle	49.24	52.08	50.66
Turned Hexagon	54.21	58.69	56.45
Waves	42.78	59.94	51.36

8 References

[S1] F. Marchetti, C. Di Nicola, R. Pettinari, I. Timokhin, C. Pettinari, *J. Chem. Educ.* **2012**, *89*, 652-655.
